# Transcriptome Analysis Reveals Immunomodulatory Effect of Spore-Displayed p75 on Human Intestinal Epithelial Caco-2 Cells

**DOI:** 10.3390/ijms232314519

**Published:** 2022-11-22

**Authors:** Soo-Ji Kang, Ji-Su Jun, Kwang-Won Hong

**Affiliations:** Department of Food Science and Biotechnology, College of Life Science and Biotechnology, Dongguk University, Goyang-si 10326, Republic of Korea

**Keywords:** spore surface display, p75 protein, immunomodulation, Caco-2 cells, RNA-sequencing

## Abstract

*Lacticaseibacillus rhamnosus* GG (LGG) can promote intestinal health by modulating the immune responses of the gastrointestinal tract. However, knowledge about the immunomodulatory action of LGG-derived soluble factors is limited. In our previous study, we have displayed LGG-derived p75 protein on the spore surface of *Bacillus subtilis*. The objective of this study was to determine the effect of spore-displayed p75 (CotG-p75) on immune system by investigating transcriptional response of Caco-2 cells stimulated by CotG-p75 through RNA-sequencing (RNA-seq). RNA-seq results showed that CotG-p75 mainly stimulated genes involved in biological processes, such as response to stimulus, immune regulation, and chemotaxis. KEGG pathway analysis suggested that many genes activated by CotG-p75 were involved in NF-ĸB signaling and chemokine signaling pathways. CotG-p75 increased cytokines and chemokines such as *CXCL1*, *CXCL2*, *CXCL3*, *CXCL8*, *CXCL10*, *CCL20*, *CCL22*, and *IL1B* essential for the immune system. In particular, CotG-p75 increased the expression levels of NF-ĸB-related genes such as *NFKBIA*, *TNFAIP3*, *BIRC3*, *NFKB2*, and *RELB* involved in immune and inflammatory responses. This study provides genes and pathways involved in immune responses influenced by CotG-p75. These comprehensive transcriptome profiling could be used to elucidate the immunomodulatory action of CotG-p75.

## 1. Introduction

Probiotics are known to have beneficial effects on health by maintaining immune homeostasis and improving gut barrier function [1,2]. They can balance anti-inflammatory and pro-inflammatory responses through various mechanisms [3]. *Lacticaseibacillus rhamnosus* GG (LGG) is a beneficial Gram-positive bacterium isolated from human gut [4]. It is one of the most researched probiotic bacteria in clinical studies for treating and preventing many gastrointestinal (GI) disorders [5]. One of the mechanisms involved in the probiotic action of LGG is its high immune activity [6]. Many studies have reported that LGG has an immunostimulatory effect on both innate and adaptive immunity components [7,8,9]. Immunomodulatory effects of cell wall components and soluble factors derived from LGG have been demonstrated [7,10,11,12]. Specifically, p40 protein derived from LGG can upregulate the expression of a proliferation-inducing ligand (APRIL) expression and promote immunoglobulin A (IgA) production in intestinal epithelial cells [13]. In addition, p40 participates in the regulation of innate immunity and Th1 immune response [14]. However, immunomodulatory effects of LGG-derived soluble proteins remain unclear.

As one of the soluble proteins derived from LGG, p75 can prevent cytokine-induced apoptosis and promote intestinal epithelial cell homeostasis [15,16]. This protein can protect intestinal epithelial tight junctions and barrier functions from disruption induced by hydrogen peroxide [17]. It is suggested that p75 might have immunomodulatory effects. Several studies have reported that LGG soluble factors exhibit immunomodulatory effects similar to those of LGG by increasing Th1-type cytokines or heat shock proteins [10,12]. Although the specific immunomodulatory action of p40 has been reported, the action of p75 has not been reported yet. In a previous study, we have displayed p75 on the spore surface of *Bacillus subtilis* using spore surface display technology [18]. We have demonstrated stable expression of spore-displayed p75 (CotG-p75) and its antimicrobial activity against *Listeria monocytogenes* [18].

Human intestinal epithelial cell lines HT-29 and Caco-2 isolated from colon adenocarcinomas are widely used in in vitro attachment and mechanistic studies [19,20]. The HT-29 cell line is essentially undifferentiated and comprises columnar absorptive cells. It has been used to study the adhesion or invasion of microorganisms due to its mucus secreting properties [21]. The Caco-2 cell line forms a polarized monolayer with a brush border [22]. It can spontaneously differentiate, exhibiting high homology with enterocytes in the intestinal epithelium [23]. It has been mainly used to elucidate the action of pathways or mechanisms of food components [24,25]. However, these different characteristics of Caco-2 and HT-29 cells can lead to different patterns in gene expression profiling results [26]. We have demonstrated that CotG-p75 can affect cell development, cell differentiation, and mucin production in HT-29 cells in our recent study [27]. Therefore, we evaluated the transcriptional response of Caco-2 cell line to the response of CotG-p75.

In this study, we aimed to identify the effect of CotG-p75 on human intestinal epithelial Caco-2 cells by transcriptional analysis based on RNA-seq. We characterized the genes, biological processes, and pathways involved in immune response through functional analyses of differentially expressed genes (DEGs), gene ontology (GO) enrichment, and KEGG pathway. Genome-wide transcriptome analysis is useful for understanding the immunomodulatory effect of CotG-p75 on human intestinal epithelial Caco-2 cells.

## 2. Results

### 2.1. Summary of Transcriptome Sequencing Data

Transcriptome changes in Caco-2 cells treated with wild-type spores or CotG-p75 for 3 h were investigated by RNA-seq. Sequencing data from three biological triplicates of each sample, control cells (CON), wild-type spore-treated cells (WT), and CotG-p75-treated cells, were generated. Quality control results are shown in Table S1. Raw data generated by sequencing ranged from 68.3 to 89.3 million reads per sample. After trimming low-quality reads, clean reads ranged from 58.4 to 88.5 million reads per sample and accounted for more than 95% of the raw reads. In addition, more than 98% of clean reads were mapped to the human reference genome, with 95% of them having a quality score of more than 30 in all samples. GC contents of all samples were also close to the ideal GC distribution. These results confirmed that the sequencing data had sufficient quality for further functional analysis.

### 2.2. Analysis of Differentially Expressed Genes in Caco-2 Cells Stimulated with CotG-p75

**Figure 1:**
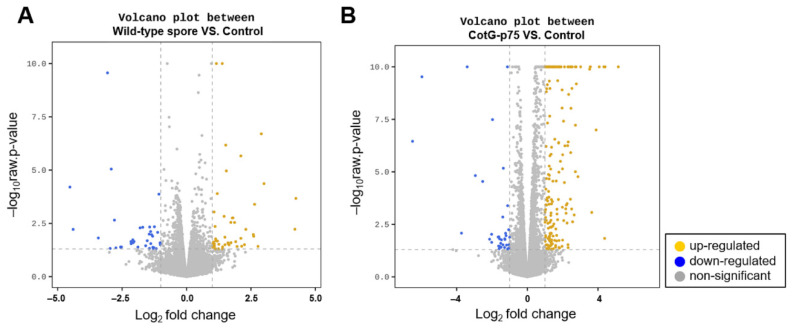
Volcano plot of differentially expressed genes (DEGs) in wild-type spores-stimulated cells versus control cells (**A**) and CotG-p75-stimulated cells versus control cells (**B**). Scattered yellow and blue dots indicate up-regulated and down-regulated DEGs with significant differences corresponding to an adjusted *p*-value < 0.05, respectively. Gray dots indicate non-differentially expressed genes.

**Figure 2:**
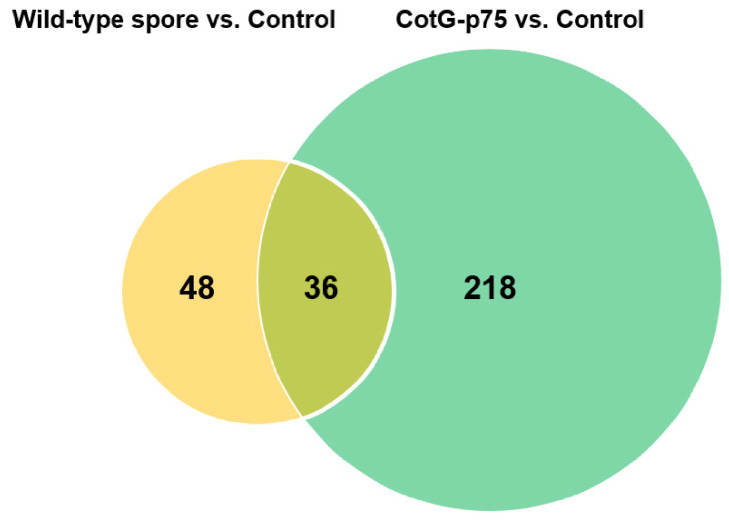
Numbers of common and unique differentially expressed genes (DEGs) in Caco-2 cells treated with wild-type spores and CotG-p75 compared to control. The overlapping region indicates the number of DEGs expressed commonly in two groups. The non-overlapping regions indicate the number of DEGs expressed only in comparison groups.

**Figure 3:**
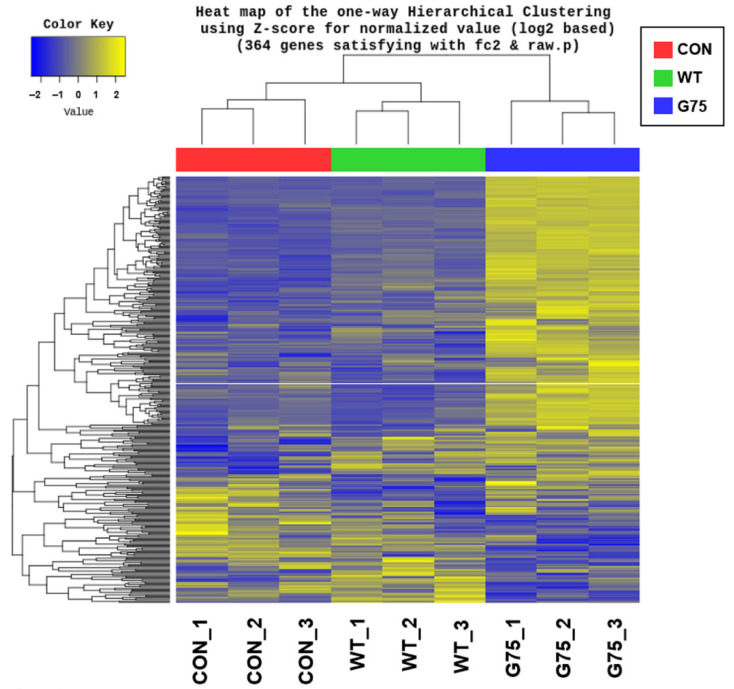
Hierarchical clustering of differentially expressed genes (DEGs) in Caco-2 cells unstimulated (CON) and stimulated by wild-type spores (WT) or CotG-p75 (G75). Each row represents one of the common genes. Each column represents each sample. The color scale shows the gene expression standard deviations from the mean represented as Z-score, with yellow indicating up-regulation and blue indicating down-regulation.

To determine transcriptional responses of human intestinal epithelial cells to stimulation by CotG-p75, gene expression levels were monitored by RNA-seq. Caco-2 cells were stimulated with wild-type spores or CotG-p75 for 3 h. Gene expression levels were then compared with those of the control group. Volcano plots in Figure 1 visualize DEGs in cells stimulated with wild-type spores (Figure 1A) or CotG-p75 (Figure 1B) compared to controls. A total of 84 (48 up-regulated and 36 down-regulated) and 254 (218 up-regulated and 36 down-regulated) DEGs were identified in Caco-2 cells treated with wild-type spores and CotG-p75, respectively. As illustrated in Figure 2, the Venn diagram depicts the shared 36 overlapping DEGs in wild-type spore-treated and CotG-p75-treated groups. Hierarchical clustering showed that the gene expression patterns in the CotG-p75 treated group was significantly different from that in the control or the wild-type spore-treated group (Figure 3). These results suggested that CotG-p75 induced transcriptome changes. CotG-p75 appeared to have a greater influence on Caco-2 cells than wild-type spores. DEG analysis results for comparisons of wild-type spore-stimulated cells vs. control, CotG-p75-stimulated cells vs. control, and CotG-p75-stimulated cells vs. wild-type spore-stimulated cells are provided in Tables S2–S4, respectively.

### 2.3. Gene Ontology Enrichment Analysis

**Figure 4:**
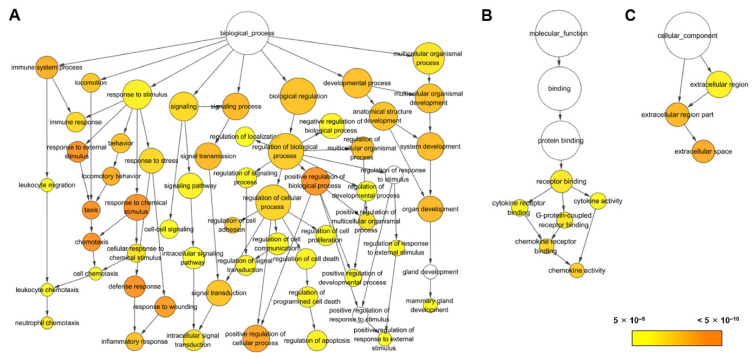
Gene ontology (GO) term networks of differentially expressed genes between CotG-p75 treated Caco-2 and control. Networks were generated by Cytoscape BiNGO. All significantly enriched GO terms in biological process (**A**), molecular function (**B**), and cellular component (**C**) are presented. Circle size represents GO hierarchy. Yellow shade represents enrichment level. Threshold of hypergeometric distribution of functional annotation was set at corrected *p*-value < 0.00005.

To identify biological functions of genes in Caco-2 cells stimulated by wild-type spores and CotG-p75 in Caco-2 cells, GO enrichment analysis was conducted. A total of 84 genes in wild-type spore-treated cells and 254 genes in CotG-p75-treated cells were classified into biological process (BP), molecular function (MF), and cellular component (CC) categories. In wild-type spore-treated cells, most of DEGs were enriched in BP terms, including response to stimulation (GO:0042221) and locomotion (GO:0040011) (Figure S1). The 254 DEGs found in CotG-p75-treated cells were significantly enriched in 469 BP, 24 MP, and 15 CC terms. Table 1 lists the top of 20 enriched GO terms. Figure 4 depicts their interaction network. Most genes activated by CotG-p75 were enriched in response to stimulation (GO:0042221), immune system process (GO:0002376), locomotion (GO:0040011), signaling (GO:0023052), biological regulation (GO:0065007), developmental process (GO:0032502), and multicellular organismal process (GO:0032501) in BP. Some genes in MF and CC were enriched in receptor binding (GO:0005102) and extracellular region (GO:0005576), respectively. The two overlapping BP subcategories, response to chemical (GO:0042221) and locomotion (GO:0040011), between the wild-type spore- and CotG-p75-treated cells suggested that these BP were influenced not only by CotG-p75, but also by the p75 carrier itself, wild-type spores. These results showed that CotG-p75 mainly stimulated BPs in Caco-2 cells, especially response to stimulus, immune processes, and cytokine/chemokine activity. GO enrichment analysis results for comparisons of wild-type spore-stimulated cells vs. control, CotG-p75-stimulated cells vs. control, and CotG-p75-stimulated cells vs. wild-type spore-stimulated cells are provided in Tables S5–S7, respectively.

### 2.4. KEGG Pathway Enrichment Analysis

**Figure 5:**
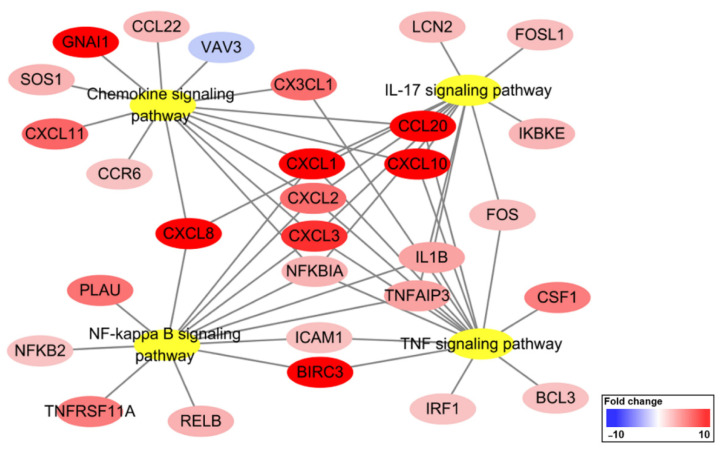
Network visualization of top four KEGG pathways and genes enriched in CotG-p75 stimulated Caco-2 cells. Yellow circles represent KEGG pathways. Red and blue circles represent the enriched genes colored according to their fold change values. Fold change value is expressed based on the color key.

**Figure 6:**
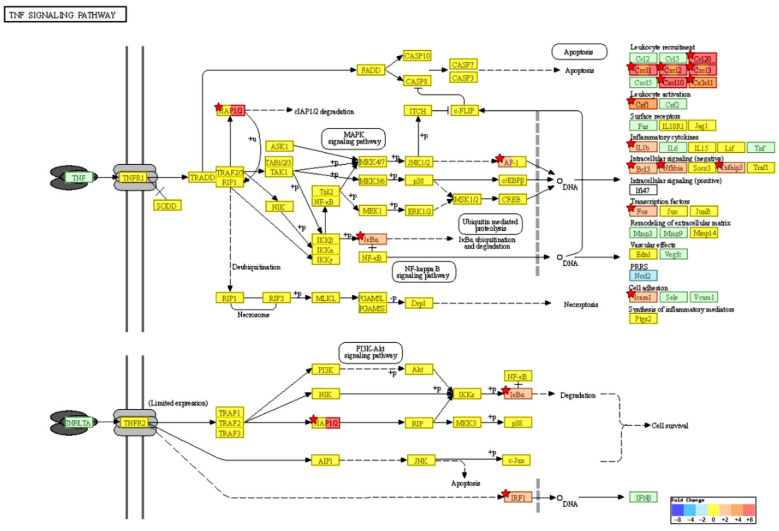
TNF signaling pathway (KEGG map, has04668) of differentially expressed genes from CotG-p75 stimulated Caco-2 cells. Fold change value is expressed based on the color key. Modules with significantly selected DEGs are marked with a red star symbol. Blue indicates modules to which down-regulated genes are mapped. Red indicates modules to which up-regulated genes are mapped. Green indicates modules made up of genes present in a species whose expression cannot be determined.

**Figure 7:**
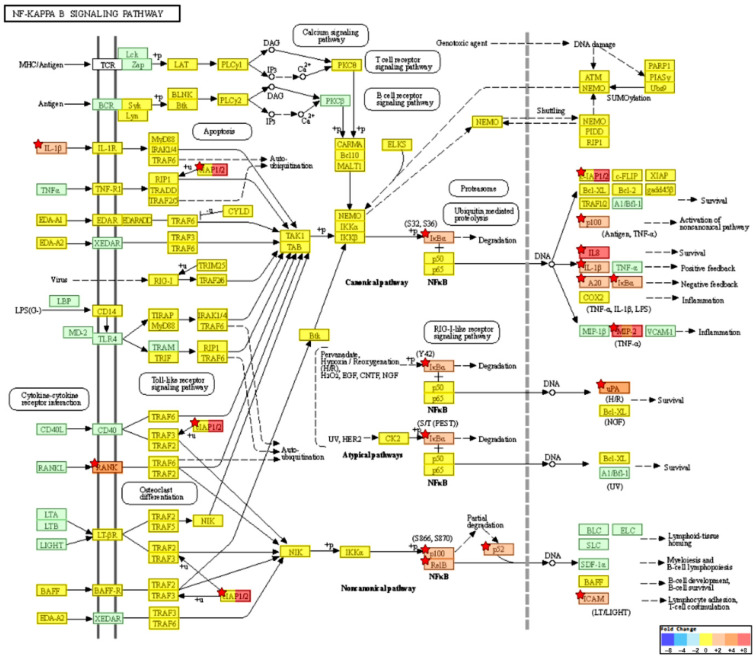
NF-κB signaling pathway (KEGG map, has04064) of differentially expressed genes from CotG-p75 stimulated Caco-2 cells. Fold change value is expressed based on the color key. Modules with significantly selected DEGs are marked with a red star symbol. Blue indicates modules in which down-regulated genes are mapped. Red indicates modules in which up-regulated genes are mapped. Green indicates modules made up of genes present in a species whose expression cannot be determined.

To investigate pathways affected by CotG-p75 treatment of Caco-2 cells, KEGG pathway analysis was performed. The 254 DEGs found in CotG-p75-treated Caco-2 cells were enriched in 79 pathways. Information on the top 10 pathways is shown in Table 2. Many genes belonged to multiple pathways, mainly the TNF signaling pathway (has04668), the IL-17 signaling pathway (has04657), the NF-κB signaling pathway (has04064), and the chemokine signaling pathway (has04062). Relationships between the top four KEGG pathways and the genes enriched in each pathway are shown in Figure 5. Some genes, such as *CXCL1*, *CXCL2*, *CXCL3*, *NFKBIA*, *CXCL8*, *CXCL10*, *CCL20*, *IL1B*, and *TNFAIP3*, were involved in all pathways. The KEGG map of the TNF signaling pathway is visualized in Figure 6. Cytokines and chemokines such as *CXCL1*, *CXCL2*, *CXCL3*, *CXCL10*, *CX3CL1*, *IL1B*, *CCL20*, and *CSF1* were up-regulated by CotG-p75. As shown in Figure 7, genes related to NF-κB signaling pathway, such as *NFKBIA*, *NFKB2*, *RELB*, and *ICAM1*, were up-regulated. Cytokines and chemokines induced by the NF-κB signaling pathway, such as *CXCL1*, *CXCL2*, *CXCL3*, *CXCL8*, and *IL1B*, were up-regulated. FC values of genes included in these pathways are listed in Table 3. These results suggested that CotG-p75 markedly influenced immune related genes. KEGG pathway analysis results for comparisons of wild-type spore-stimulated cells vs. control, CotG-p75-stimulated cells vs. control, and CotG-p75-stimulated cells vs. wild-type spore-stimulated cells, are provided in Tables S8–S10, respectively. The top 10 KEGG pathways enriched in DEGs between wild-type spore treated Caco-2 and control was presented in Figure S2.

### 2.5. RNA-Seq Data Validation by RT-qPCR

**Figure 8:**
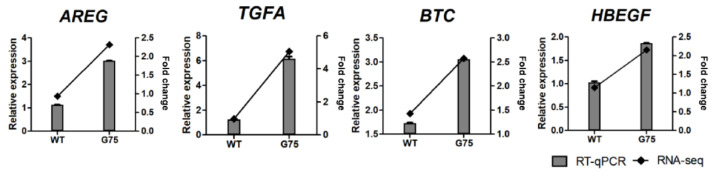
Real-time quantitative PCR validation of RNA-seq data for selected genes associated with EGFR signaling from Caco-2 cells stimulated by CotG-p75. Right and left *y*-axis represent fold change of RNA-seq data and relative expression of RT-qPCR data, respectively. Relative mRNA expression was compared with spore-untreated control. RT-qPCR analysis for mRNA expression of selected genes was normalized against *GAPDH*. RT-qPCR data are presented as mean ± standard deviation (*n* = 3).

**Figure 9:**
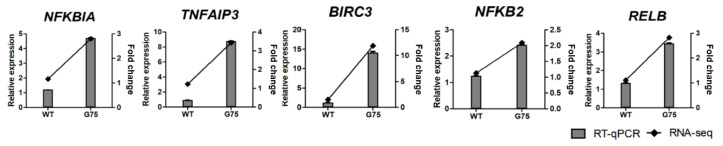
Real-time quantitative PCR validation of RNA-seq data for selected genes associated with NF-κB signaling from Caco-2 cell. Right and left *y*-axis represent fold change of RNA-seq data and relative expression of RT-qPCR data, respectively. Relative mRNA expression was compared with spore-untreated control. RT-qPCR analysis for mRNA expression of selected genes was normalized against *GAPDH*. RT-qPCR data are presented as mean ± standard deviation (*n* = 3).

**Figure 10:**
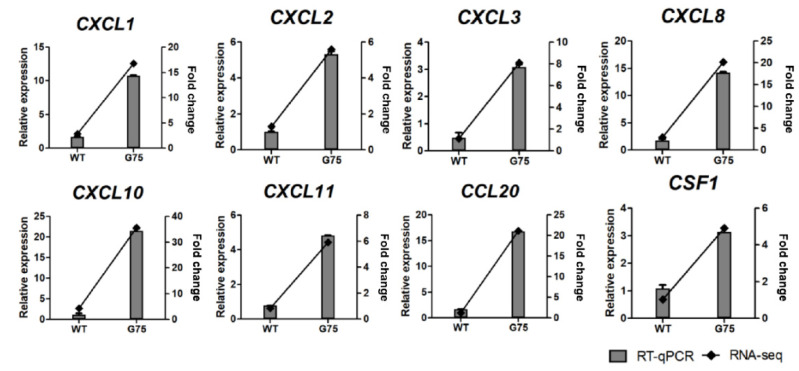
Real-time quantitative PCR validation of RNA-seq data for selected genes associated with immune response from Caco-2 cells stimulated by CotG-p75. Right and left *y*-axis represent fold change of RNA-seq data and relative expression of RT-qPCR data, respectively. Relative mRNA expression was compared with spore-untreated control. RT-qPCR analysis for mRNA expression of selected genes was normalized against *GAPDH*. RT-qPCR data are presented as mean ± standard deviation (*n* = 3).

To verify DEGs identified by RNA-seq results, RT-qPCR analysis was conducted. GO and the KEGG pathway enrichment analysis revealed that CotG-p75-activated genes were related to immune processes, chemotaxis, TNF signaling, and chemokine signaling pathways. Therefore, four genes associated with EGFR signaling (*AREG*, *TGFA*, *BTC*, and *HBEGF*), five genes associated with NF-κB signaling (*BIRC3*, *NFKBIA*, *TNFAIP3*, *NFKB2*, and *RELB*), and eight genes associated with immune system (*CXCL1*, *CXCL2*, *CXCL3*, *CXCL8*, *CXCL10*, *CXCL11*, *CCL20*, and *CSF1*) were selected for validation by RT-qPCR. RT-qPCR confirmed that the DEG expression trend was consistent with RNA-seq results (Figure 8, Figure 9 and Figure 10). Additionally, the mRNA levels of selected genes were increased dose-dependently by CotG-p75 (Figures S3–S5). These results confirmed that the regulation of suggested genes affected those signaling pathways of Caco-2 cells.

### 2.6. Effects of CotG-p75 on HBEGF and CCL20 Protein Expression

**Figure 11:**
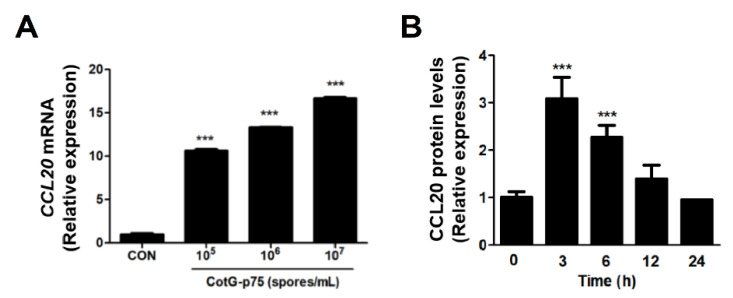
Effects of Cotable 75. on *HBEGF* mRNA expression (**A**) and HBEGF protein production (**B**) in Caco-2 cells. In panel (A), cells were treated with various concentrations (10^5^, 10^6^, and 10^7^ spores/mL) of CotG-p75 for 3 h. In panel (**B**), cells were treated with CotG-p75 (10^7^ spores/mL) for various time periods (0, 3, 6, 12, and 24 h). Both mRNA expression levels and protein production levels in treated groups were compared with those of the control group. All data are expressed as mean ± standard deviation (*n* = 3). Asterisks (***) indicate a significance difference from the control (***, *p* < 0.001).

**Figure 12:**
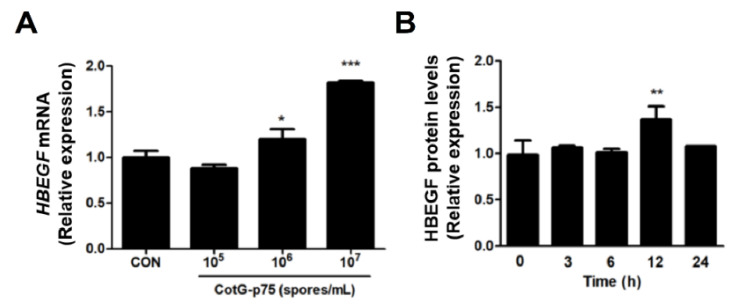
Effects of CotG-p75 on *CCL20* mRNA expression (**A**) and CCL20 protein production (**B**) in Caco-2 cells. In panel (**A**), cells were treated with various concentrations (10^5^, 10^6^, and 10^7^ spores/mL) of CotG-p75 for 3 h. In panel (**B**), cells were treated with CotG-p75 (10^7^ spores/mL) for various time periods (0, 3, 6, 12, and 24 h). Both mRNA expression levels and protein production levels in treated groups were compared with those of the control group. All data are expressed as mean ± standard deviation (*n* = 3). Asterisks (*) indicate a significance difference from the control (*, *p* < 0.05; **, *p* < 0.01; ***, *p* < 0.001).

*HBEGF* and *CCL20* genes associated with the EGFR signaling pathway and immune system were selected. CotG-p75 significantly increased mRNA expression level of *HBEGF* in a dose-dependent manner (Figure 11A). Protein expression of HBEGF was significantly increased by CotG-p75, reaching the maximum level at 12 h (Figure 11B). A dose-dependent increase in mRNA expression of *CCL20* was observed in Caco-2 cells treated with CotG-p75 (Figure 12A). Protein expression of CCL20 was also increased in Caco-2 cells treated with CotG-p75 (Figure 12B). Protein level of CCL20 peaked at 3 h, but returned to baseline after 12 h. These data indicated that CotG-p75 affected HBEGF release and the immune system.

### 2.7. Comparison of Transcriptional Responses of HT-29 and Caco-2 Cells Stimulated by CotG-p75

**Figure 13:**
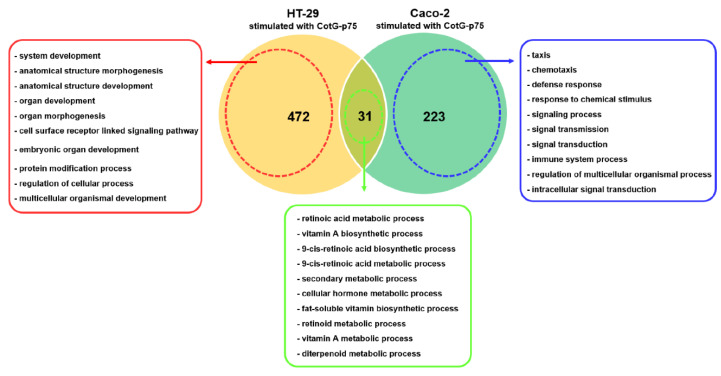
Numbers of common and unique DEGs in HT-29 and Caco-2 cells stimulated with CotG-p75. Top 10 gene ontology terms of common and unique DEGs in HT-29 and Caco-2 are presented in the green, red, and blue boxes.

To compare responses of HT-29 and Caco-2 cells stimulated by CotG-p75, GO enrichment analyses of common and unique DEGs of two cells were performed. Numbers of common and unique DEGs in HT-29 and Caco-2 are shown in the Venn diagram (Figure 13). Top 10 GO terms from GO enrichment analysis results of these DEGs are presented. A total of 31 genes were commonly differentially expressed in both cells. They were mainly involved in vitamin A biosynthetic process. A total of 472 genes were differentially expressed only in HT-29 cells. They were associated with developmental processes, such as system development, anatomical structure development, and organ development [27]. Meanwhile, a total of 233 genes were activated only in Caco-2 cells. They were involved in the immune system and chemotaxis, such as taxis, chemotaxis, and response to chemical stimulus in the present study. These results indicated that different transcriptional responses were induced by CotG-p75, according to cell type, even with the same treatment.

## 3. Discussion

Transcriptome analysis was conducted for human intestinal epithelial Caco-2 cells treated by CotG-p75. Due to different characteristics between HT-29 and Caco-2 cell lines, gene expression profiling results also showed different patterns [26]. We have previously investigated the effect of CotG-p75 on HT-29 cell line [27]. In the present study, the effect of CotG-p75 on Caco-2 cell line was evaluated. Different transcriptional responses were observed between HT-29 and Caco-2 cells treated with CotG-p75. The reason why these two cell lines responded to CotG-p75 differently was that each cell line had a unique gene expression profile. Bourgine et al. (2012) have found that gene expression profiles differed significantly between various intestinal epithelial cell lines [26]. Moreover, previous studies have shown that gene expression profiles can be altered depending on cell line origin, cell culture environment, and cell differentiation [28,29,30]. Similarly to these results, in the present study, gene expression profiles were significantly different when expression profiles of genes of signaling pathways related to cell survival, development, and immune response were compared between HT-29 and Caco-2 cells.

GO enrichment analysis showed that CotG-p75 affected biological processes such as response to stimulus, immune processes, and cytokine/chemokine activity in Caco-2 cells. KEGG pathway analysis showed that many genes involved in the TNF signaling pathway, IL-17 signaling pathway, NF-κB signaling pathway, and chemokine signaling pathway were regulated by CotG-p75. Genes mainly involved in those GO terms and pathways were extracted through GO and KEGG pathway analysis. These genes were mainly cytokines, chemokines, and NF-κB target genes, such as *CXCL1*, *CXCL2*, *CXCL3*, *CXCL8*, *CXCL10*, *CXCL11*, *CCL20*, *CCL22*, *CX3CL1*, *IL1B*, *CSF1*, *ICAM1*, *BIRC3*, *TNFAIP3*, *RELB*, *NFKB2*, and *NFKBIA*.

Similarly to transcriptional responses of HT-29 cells to CotG-p75, expression levels of genes encoding EGFR ligands (*AREG*, *TGFA*, *BTC*, and *HBEGF*) were increased. The EGFR signaling pathway plays an important role in maintaining epithelial homeostasis by promoting cell proliferation, differentiation, migration, and survival [31,32]. Moreover, many studies have revealed that LGG and p40 transactivated EGFR through HBEGF release in intestinal epithelial cell lines [33,34,35,36].

One of the major findings of the KEGG pathway analysis was that CotG-p75 increased transcription levels of NF-κB-related factors such as *NFKB2*, *RELB*, *BIRC3*, *NFKBIA*, and *TNFAIP3* in the NF-κB signaling pathway. *BIRC3*, *NFKBIA*, and *TNFAIP3* genes are negative regulators of inflammatory signaling pathways [37]. NF-κB is a member of the family of transcription factors that can regulate genes involved in inflammation, immune response, cell proliferation, differentiation, and survival [38]. Activation of NF-κB is involved in the regulation of intestinal inflammation and maintenance of epithelial barrier function [39,40,41].

Specifically, tumor necrosis factor α-induced protein 3, TNFAIP3 (or A20), is induced by TNF-mediated NF-κB activation [42]. It can inhibit NF-κB activation [42]. Severe intestinal inflammation has been shown in TNFAIP3-deficient mice [37,42]. Reduced expression of TNFAIP3 in the intestinal mucosa has been observed in patients with Crohn’s disease [43]. TNFAIP3 can enhance intestinal epithelial barrier integrity and maintain intestinal homeostasis through regulation of tight junction proteins [44]. NFKBIA is one of the most important negative regulators of NF-κB in that it can inhibit the activity of dimeric NF-κB/REL complexes [45]. *BIRC3* (cIAP2) is an important anti-apoptotic and pro-proliferation gene of the IAP (inhibitor of apoptosis) family [46]. It is known to be up-regulated by NF-κB [46]. Therefore, up-regulation of these negative regulators of NF-κB in response to CotG-p75 is relevant to the inhibition of the NF-κB signaling pathway.

Another interesting finding was that expression levels of various cytokines and chemokines, such as *CXCL1*, *CXCL2*, *CXCL3*, *CXCL8*, *CXCL10*, *CXCL11*, *CCL22*, *CX3CL1*, *IL1B*, and *CSF1*, were increased by CotG-p75. Correspondingly, KEGG pathway analysis revealed that CotG-p75 affected NF-κB regulation in Caco-2 cells. Cytokines and chemokines are involved in immune regulation and inflammatory responses under the control of NF-κB [47]. Chemokines can modulate immune responses by regulating the migration and activation of lymphocytes, neutrophils, and basophils [48]. In addition, expression levels of CCL20-encoding gene and CCL20 protein were increased in CotG-p75-treated cells temporarily. A previous study has suggested that CCL20 might be involved in intestinal epithelial repair, restitution, and migration in Caco-2 cells [49]. In addition, chemokines have similar structures to antibacterial peptides with antibacterial effects against various bacteria [50,51]. Thus, increased levels of cytokines and chemokines are expected to help prevent bacterial infection and promote the immune responses.

In addition to cytokines and chemokines, transcriptional induction of negative regulators of NF-κB was observed in Caco-2 cells stimulated with CotG-p75. These results paralleled findings of in vivo and in vitro studies of other probiotic strains. Probiotics strains such as *L. salivarius* UCC118 and *L. acidophilus* LAFTI-L10 can induce the expression of negative regulatory genes of NF-κB (such as *BIRC3*, *NFKBIA*, *TNFAIP3*, *RELB*, and *NFKB2*) and chemokine genes (such as *CXCL1*, *CXCL2*, *CXCL3*, *CXCL8*, *CXCL10*, *CXCL11*, *CCL20*, and *CCL22*) in Caco-2 cells and healthy adults [52,53,54].

In terms of intestinal barrier protection, NF-κB-induced inflammatory response is a double-edged sword. NF-κB activation helps establish one of the first lines of defense against bacteria and fungi by inducing pro-inflammatory cytokines and chemokines [55]. Conversely, deregulated inflammatory responses can cause excessive or long-lasting tissue damage, contributing to the development of acute or chronic inflammatory diseases [38]. In the current study, CotG-p75 induced pro-inflammatory genes and negative regulatory genes of NF-κB. Thus, it is difficult to conclude whether the pro- or anti-inflammatory response is predominant in Caco-2 cells treated with CotG-p75. These results show that CotG-p75 can activate immune responses mediated by the NF-κB signaling pathway in human epithelial Caco-2 cells.

## 4. Materials and Methods

### 4.1. Spore Preparation

*B. subtilis* 168 spores with recombinant pUB19-*cotG-p75* were constructed in accordance with the protocol of a previous study [18]. In summary, sporulation of *B. subtilis* 168 spores and recombinant spores (CotG-p75) were induced with Difco Sporulation Medium (DSM) consisting of 0.8% nutrient broth, 0.025%, MgSO_4_·7H_2_O, 0.1% KCl, 10 M MnCl_2_, 1.0 M FeSO_4_·7H_2_O, and 1.0 mM Ca(NO_3_)_2_. After cultivation at 37 °C with shaking at 150 rpm for 62 h, the spores were collected and resuspended in 50 mM sodium phosphate buffer (pH 7.2). Spore suspensions were treated with lysozyme for 1 h at 4 °C to lyse residual vegetative cells. Spores were then washed five times with 50 mM sodium phosphate buffer (pH 7.2). After centrifugation at 5000 rpm for 10 min, harvested spores were resuspended in 1–2 mL of phosphate-buffered saline (PBS). The number of purified spores was measured on LB agar using the colony counting method. Spores were adjusted to 10^8^ CFU/mL and stored at 4 °C.

### 4.2. Cell Culture and Treatments

Human intestinal epithelial Caco-2 cell line was purchased from the American Type Culture Collection (ATCC). These cells were grown in Dulbecco’s modified Eagle’s medium (DMEM), supplemented with 20% (V/V) fetal bovine serum (FBS) and 1% (V/V) penicillin-streptomycin. Cells were incubated in a humidified atmosphere at a 37 °C with 95% air and 5% CO_2_. The culture medium was replaced every other day. Cells were sub-cultured at 80–90% confluence. Cells with a passage number of 20 to 25 were used in this study. To differentiate Caco-2 cells, cells were seeded into 12-well plates at a density of 5 × 10^4^ cells per well. Experiments were performed after cells were fully differentiated for 21 days, reaching 100% confluence. For the experiments, the medium was replaced with 900 μL of fresh serum-free medium. Differentiated Caco-2 cells were exposed to 100 μL of 1 × 10^8^ CFU/mL of wild-type (treatment WT) or CotG-p75 spores for 3 h (treatment G75). Cells treated with PBS instead of spores were used as controls (treatment CON). These cells were harvested and used for subsequent transcriptome analysis and RT-qPCR.

### 4.3. Cell Viability Assay

CCK assay was used to evaluate viability of Caco-2 cells stimulated by CotG-p75 using a D-Plus CCK Cell Viability Assay Kit (DonginLS, Seoul, Republic of Korea) following the manufacturer’s protocol. Briefly, Caco-2 cells were cultured with 100 μL of cDMEM in 96-well plates at a density of 5 × 10^4^ cells/well. When the cells were fully differentiated for 21 days, cells were treated with various concentrations (10^5^–10^7^ spores/mL) of CotG-p75 for 24 h. After spent medium was removed, 100 μL of fresh medium containing 10 μL of CCK reagent was added to each well. After being incubated for 4 h, optical density was measured at 450 nm using an automated ELISA reader (Thermo Fisher Scientific, Waltham, MA, USA). The percentage of living cells was calculated as previously described [56]. Cell viability results are presented in Figure S6.

### 4.4. Total RNA Isolation, cDNA Library Construction, and RNA-Sequencing

TRIzol reagent (Invitrogen, Waltham, MA, USA) was used to extract total RNA from spore-treated and untreated Caco-2 cells according to the manufacturer’s instructions. The concentration and quality of total RNA were checked using a Nanodrop 2000 spectrophotometer (Thermo Fisher Scientific, Waltham, MA, USA). The concentration and quality of total RNA were verified before it was used for RNA-seq and RT-qPCR. Total RNA was then used for cDNA library preparations using a TruSeq Stranded mRNA LT Sample Prep Kit (part # 15031047, Rev. E, Illumina, San Diego, CA, USA) following the manufacturer’s instruction. RNA-sequencing was carried out by Macrogen (Seoul, Republic of Korea) on an Illumina NovaSeq 6000 system using NovaSeq 6000 S4 reagent kit (Illumina, San Diego, CA, USA). Quality control (QC) test of RNA-seq was evaluated with FastQC v 0.11.7 (http://www.bioinformatics.babraham.ac.uk/projects/fastqc/, accessed on 11 September 2022) [57]. To eliminate 3′ end adaptors and low-quality bases, Trimmomatic 0.38 (http://www.usadellab.org/cms/?page=trimmomatic, accessed on 11 September 2022) was used [58]. After trimming, cleaned reads were aligned to the human reference genome (hg19) obtained from the University of California Santa Cruz (UCSC) genome browser using HISAT2 version 2.1.0 software (https://ccb.jhu.edu/software/hisat2/index.shtml, accessed on 11 September 2022) [59]. Sequence data from this study were deposited into Sequence Read Archive (http://www.ncbi.nlm.nih.gov/sra, accessed on 1 October 2022) under accession number PRJNA763711.

### 4.5. Analysis of Differentially Expressed Genes (DEGs)

StringTie version 1.3.4d (https://ccb.jhu.edu/software/stringtie/, accessed on 14 September 2022) and DESeq2 software were used to estimate transcript abundances and identify DEGs between spore-treated and control groups [60,61]. Transcript abundance was calculated by fragments per kilobase of transcripts per million mapped reads (FPKM). Differentially expressed genes were defined as genes satisfying the absolute value of fold change (FC) ≥ 2 and a raw *p*-value < 0.05 between comparison groups. DEGs were obtained from three comparison groups (wild-type spores vs. control, CotG-p75 vs. control, and CotG-p75 vs. wild-type spores).

### 4.6. Functional Analyses of DEGs

Gene ontology (GO) enrichment analysis was performed to classify DEGs based on specific biological functions using a Cytoscape BiNGO (version 3.2.1) [62]. GO enrichment analysis covers three domains: biological process (BP), molecular function (MF), and cellular component (CC). Kyoto encyclopedia of genes and genomes (KEGG) analysis (https://www.genome.jp/kegg/pathway.html, accessed on 16 September 2022) was used to identify metabolic pathways or signal transduction pathways significantly enriched in differentially expressed genes [63]. GO terms and pathways satisfying adjusted *p*-value < 0.05 were considered enriched significantly.

### 4.7. Reverse Transcription-Quantitative Polymerase Chain Reaction (RT-qPCR) Confirmation

A total of 17 genes associated with EGFR signaling (*AREG*, *TGFA*, *BTC*, and *HBEGF*), NF-κB signaling pathway (*BIRC3*, *NFKBIA*, *TNFAIP3*, *NFKB2*, and *RELB*), chemotaxis, and immune system process (*CXCL1*, *CXCL2*, *CXCL3*, *CXCL8*, *CXCL10*, *CXCL11*, *CCL20*, and CSF1) that were found to be differentially expressed from DEGs analysis were selected and validated by RT-qPCR. Total RNA (2 μg) was used to synthesize cDNA using random hexamer (Roche, Basel, Switzerland) and M-MLV reverse transcriptase (Promega, Madison WI, USA) following the manufacturer’s protocols. RT-qPCR was conducted at 95 °C for 10 s, followed by 40 cycles of 95 °C for 5 s and 60 °C for 30 s using a SYBR-Green PCR Master Mix kit (Takara, Shiga, Japan) on a CFX Connect Real-Time System (Bio-Rad, Hercules, CA, USA). Gene-specific primers were obtained from OriGene technologies (Rockville, MD, USA). They are presented in Table S11. The RT-qPCR analysis was conducted in triplicate independently. Target genes were normalized against GAPDH gene as an internal control. Relative expression levels of the selected genes were calculated with the 2^−ΔΔCt^ method [64].

### 4.8. Heparin Binding-Epidermal Growth Factor (HBEGF) and CC Motif Chemokine Ligand (CCL20) Protein Expression

ELISA assay was performed to measure HBEGF and CCL20 protein levels using a human mucin-5AC (MUC5AC) ELISA Kit (Cusabio Biotech Co., Wuhan, China) and a human MIP-3a (CCL20) ELISA kit (AbFrontier, Seoul, Republic of Korea), respectively, following each manufacturer’s protocol. Caco-2 cells were stimulated by CotG-p75 (10^7^ spores/mL) for 0, 3, 6, 12, and 24 h. Cells not treated with spores were used as controls. Amounts of HBEGF and CCL20 proteins in cell culture supernatants are expressed as picograms of HBEGF or CCL20 per milliliter of supernatant. Results are expressed as relative expression by normalizing protein expression levels for CotG-p75-treated conditions versus control at all time courses.

### 4.9. Statistical Analysis

All experiments were performed independently in biological triplicates. For RNA-seq, data analysis and visualization were performed using R package. Criteria for differential expression were: |FC| > 2 and raw *p*-value < 0.05. The cutoff for GO and KEGG analyses was adjusted *p*-value < 0.05. For RT-qPCR analysis, data are expressed as mean ± standard deviation (SD). Statistical analyses such as one-way ANOVA and Tukey–Kramer post hoc analysis were performed using GraphPad Prism 5.0 software (GraphPad, San Diego, CA, USA).

## 5. Conclusions

In conclusion, transcriptome analysis identified genes, biological processes, and pathways of human epithelial Caco-2 cells in response to CotG-p75. Several genes activated by CotG-p75 were involved in biological processes, such as the response to stimulus, immune response, and chemotaxis. CotG-p75 induced expression of genes associated with EGFR signaling pathway ligands, cytokines, chemokines, and negative regulators of NF-κB. These results are important since they could reveal immune responses of human intestinal epithelial cells affected by CotG-p75. In addition, elucidating the transcriptional response of human intestinal epithelial cells to CotG-p75 can help us draw evidence-based targets for in vivo experiments. This study provided an improved understanding of the immunomodulatory effect of CotG-p75 on human intestinal epithelial Caco-2 cells.

## Figures and Tables

**Table 1:** Gene ontology (GO) enrichment analysis of differentially expressed genes in Caco-2 cells stimulated with CotG-p75.

GO ID	GO Description	Adjusted *p*-Value ^a^	Cluster Frequency ^b^	Total Frequency ^c^
Biological process				
0042221	response to chemical stimulus	3.70 × 10^−15^	54/202 (26.7%)	1466/17,784 (8.2%)
0042330	taxis	5.20 × 10^−15^	20/202 (9.9%)	169/17,784 (0.9%)
0006935	chemotaxis	5.20 × 10^−15^	20/202 (9.9%)	169/17,784 (0.9%)
0006952	defense response	6.97 × 10^−13^	32/202 (15.8%)	621/17,784 (3.4%)
0009605	response to external stimulus	1.03 × 10^−12^	30/202 (14.8%)	552/17,784 (3.1%)
0048518	positive regulation of biological process	1.43 × 10^−12^	63/202 (31.1%)	2210/17,784 (12.4%)
0009611	response to wounding	3.92 × 10^−12^	29/202 (14.3%)	543/17,784 (3.0%)
0048522	positive regulation of cellular process	8.84 × 10^−12^	58/202 (28.7%)	2006/17,784 (11.2%)
0002376	immune system process	3.68 × 10^−11^	37/202 (18.3%)	949/17,784 (5.3%)
0007626	locomotory behavior	4.31 × 10^−11^	20/202 (9.9%)	273/17,784 (1.5%)
0048513	organ development	1.33 × 10^−10^	52/202 (25.7%)	1792/17,784 (10.0%)
0023046	signaling process	1.63 × 10^−10^	58/202 (28.7%)	2157/17,784 (12.1%)
0023060	signal transmission	1.63 × 10^−10^	58/202 (28.7%)	2157/17,784 (12.1%)
0048731	system development	2.26 × 10^−10^	62/202 (30.6%)	2422/17,784 (13.6%)
0051239	regulation of multicellular organismal process	2.71 × 10^−10^	38/202 (18.8%)	1068/17,784 (6.0%)
0032502	developmental process	3.55 × 10^−10^	74/202 (36.6%)	3234/17,784 (18.1%)
0007275	multicellular organismal development	3.84 × 10^−10^	70/202 (34.6%)	2971/17,784 (16.7%)
0048856	anatomical structure development	4.62 × 10^−10^	65/202 (32.1%)	2656/17,784 (14.9%)
0065007	biological regulation	5.60 × 10^−10^	122/202 (60.3%)	6941/17,784 (39.0%)
0006954	inflammatory response	5.80 × 10^−10^	20/202 (9.9%)	316/17,784 (1.7%)
Molecular function				
0008009	chemokine activity	2.47 × 10^−9^	9/202 (4.4%)	47/17,784 (0.2%)
0042379	chemokine receptor binding	6.35 × 10^−9^	9/202 (4.4%)	52/17,784 (0.2%)
0001664	G-protein-coupled receptor binding	4.92 × 10^−8^	12/202 (5.9%)	136/17,784 (0.7%)
0005102	receptor binding	5.53 × 10^−8^	31/202 (15.3%)	922/17,784 (5.1%)
0005126	cytokine receptor binding	2.13 × 10^−7^	13/202 (6.4%)	186/17,784 (1.0%)
0005125	cytokine activity	4.12 × 10^−7^	13/202 (6.4%)	197/17,784 (1.1%)
Cellular component				
0005615	extracellular space	1.91 × 10^−11^	33/202 (16.3%)	747/17,784 (4.2%)
0044421	extracellular region part	1.00 × 10^−10^	37/202 (18.3%)	983/17,784 (5.5%)
0005576	extracellular region	1.91 × 10^−7^	49/202 (24.2%)	2025/17,784 11.3%

^a^*p*-value in hypergeometric test after correction. ^b^ The denominator represents the total number of genes with GO annotation and the numerator represents the number of each GO term genes. ^c^ The denominator represents the number of reference genes with GO annotation and the numerator represents the number of references genes annotated in the listed GO term.

**Table 2:** Top 10 KEGG pathways enrichment analysis of differentially expressed genes in Caco-2 stimulated with CotG-p75.

Pathway ID	KEGG Pathway	Counts	Adjusted *p*-Val. ^a^	Genes
has04668	TNF signaling pathway	15	7.13 × 10^−13^	*CSF1*, *FOS*, *CXCL1*, *CXCL2*, *CXCL3*, *BIRC3*, *ICAM1*, *IL1B*, *CXCL10*, *IRF1*, *NFKBIA*, *BCL3*, *CCL20*, *CX3CL1*, *TNFAIP3*
has04657	IL-17 signaling pathway	13	2.95 × 10^−11^	*FOS*, *CXCL1*, *CXCL2*, *CXCL3*, *IL1B*, *CXCL8*, *CXCL10*, *LCN2*, *NFKBIA*, *CCL20*, *TNFAIP3*, *FOSL1*, *IKBKE*
has04064	NF-κB signaling pathway	13	5.90 × 10^−11^	*CXCL1*, *CXCL2*, *CXCL3*, *BIRC3*, *ICAM1*, *IL1B*, *CXCL8*, *NFKB2*, *NFKBIA*, *PLAU*, *RELB*, *TNFAIP3*, *TNFRSF11A*
has04062	Chemokine signaling pathway	14	2.50 × 10^−9^	*VAV3*, *CCR6*, *GNAI1*, *CXCL1*, *CXCL2*, *CXCL3*, *CXCL8*, *CXCL10*, *NFKBIA*, *CCL20*, *CCL22*, *CXCL11*, *CX3CL1*, *SOS1*
has05323	Rheumatoid arthritis	11	5.90 × 10^−9^	*CSF1*, *FOS*, *245972*, *CXCL1*, *CXCL2*, *CXCL3*, *ICAM1*, *IL1B*, *CXCL8*, *CCL20*, *TNFRSF11A*
has04061	Viral protein interaction with cytokine and cytokine receptor	11	9.49 × 10^−9^	*CCR6*, *CSF1*, *CXCL1*, *CXCL2*, *CXCL3*, *CXCL8*, *CXCL10*, *CCL20*, *CCL22*, *CXCL11*, *CX3CL1*
has04625	C-type lectin receptor signaling pathway	11	1.16 × 10^−8^	*EGR2*, *MRAS*, *IL1B*, *IRF1*, *ITPR1*, *NFKB2*, *NFKBIA*, *RELB*, *BCL3*, *CX3CL1*, *IKBKE*
has05417	Lipid and atherosclerosis	13	5.84 × 10^−8^	*10451*, *CYP1A1*, *FOS*, *CXCL1*, *CXCL2*, *CXCL3*, *ICAM1*, *IL1B*, *CXCL8*, *LAMA4*, *NFKBIA*, *OLR1*, *IKBKE*
has05200	Pathways in cancer	18	7.05 × 10^−8^	*ETS1*, *FGF9*, *FOS*, *RASGRP3*, *GNAI1*, *BIRC3*, *CXCL8*, *LAMA4*, *NFKB2*, *NFKBIA*, *NOS2*, *WNT4*, *SOS1*, *STAT5A*, *TGFA*, *WNT5A*, *WNT6*, *WNT7B*
has04010	MAPK signaling pathway	14	1.33 × 10^−7^	*CSF1*, *DUSP4*, *DUSP6*, *FGF9*, *MRAS*, *FOS*, *RASGRP3*, *IL1B*, *AREG*, *MEF2C*, *NFKB2*, *RELB*, *SOS1*, *TGFA*

^a^*p*-value in hypergeometric test after correction.

**Table 3:** List of genes involved in immune response in Caco-2 cells stimulated with CotG-p75.

Gene Symbol	Gene Name	Fold Change	Raw *p*-Value
*CXCL10*	C-X-C motif chemokine ligand 10	35.58	5.23 × 10^−34^
*CCL20*	C-C motif chemokine ligand 20	21.17	0.00
*CXCL8*	C-X-C motif chemokine ligand 8	20.17	4.15 × 10^−38^
*CXCL1*	C-X-C motif chemokine ligand 1	16.81	9.56 × 10^−41^
*BIRC3*	baculoviral IAP repeat containing 3	11.90	3.76 × 10^−218^
*CXCL3*	C-X-C motif chemokine ligand 3	8.10	3.93 × 10^−52^
*CXCL11*	C-X-C motif chemokine ligand 11	5.91	2.38 × 10^−3^
*CXCL2*	C-X-C motif chemokine ligand 2	5.59	6.66 × 10^−41^
*CX3CL1*	C-X3-C motif chemokine ligand 1	5.54	9.45 × 10^−9^
*PLAU*	plasminogen activator, urokinase	5.36	8.88 × 10^−22^
*TNFRSF11A*	TNF receptor superfamily member 11a	4.98	3.77 × 10^−7^
*CSF1*	colony stimulating factor 1	4.92	1.23 × 10^−25^
*TNFAIP3*	TNF α induced protein 3	3.44	7.71 × 10^−119^
*IL1B*	interleukin 1 β	3.38	2.07 × 10^−4^
*RELB*	RELB proto-oncogene, NF-κB subunit	2.83	1.63 × 10^−35^
*NFKBIA*	NF-κB inhibitor α	2.80	1.67 × 10^−75^
*SOS1*	SOS Ras/Rac guanine nucleotide exchange factor 1	2.75	7.76 × 10^−57^
*IKBKE*	inhibitor of nuclear factor κB kinase subunit epsilon	2.67	5.34 × 10^−33^
*CCL22*	C-C motif chemokine ligand 22	2.57	4.78 × 10^−2^
*LCN2*	lipocalin 2	2.50	2.14 × 10^−2^
*FOS*	Fos proto-oncogene, AP-1 transcription factor subunit	2.36	6.24 × 10^−23^
*FOSL1*	FOS like 1, AP-1 transcription factor subunit	2.34	4.92 × 10^−11^
*ICAM1*	intercellular adhesion molecule 1	2.24	9.38 × 10^−43^
*BCL3*	B-cell CLL/lymphoma 3	2.19	6.45 × 10^−35^
*CCR6*	C-C motif chemokine receptor 6	2.13	3.81 × 10^−4^
*IRF1*	interferon regulatory factor 1	2.11	7.13 × 10^−26^

## Data Availability

The datasets supporting the conclusions of this article are included within the article and its additional files. The raw sequence data have been submitted to the Sequence Read Archive (SRA) under Bioproject accession number PRJNA763711 (https://www.ncbi.nlm.nih.gov/sra/PRJNA763711 (accessed on 1 October 2022)) and reference BioSample accession number SAMN21447882–4 (https://www.ncbi.nlm.nih.gov/biosample/21447882–4 (accessed on 1 October 2022)).

## Supplementary Materials

The following supporting information can be downloaded at: https://www.mdpi.com/article/10.3390/ijms232314519/s1.
