# Supplementary material for: Transcriptome Analysis Reveals Immunomodulatory Effect of Spore-Displayed p75 on Human Intestinal Epithelial Caco-2 Cells
Source: Int J Mol Sci. 2022 Nov 22;23(23):14519. doi: 10.3390/ijms232314519 (PMC9739243; doi:10.3390/ijms232314519)
Supplement: Supplementary file 1 [file ijms-23-14519-s001.zip › ijms-1997195-supplementary.pdf]

Journal name:

International Journal of Molecular Sciences

Manuscript title:

Transcriptome analysis reveals immunomodulatory effect of spore-displayed p75 on human intestinal epithelial Caco-2 cells

The names of the authors:

Soo Ji Kang[1], Ji Su Jun[1], and Kwang Won Hong[1,*]

The affiliation and address of the authors:

[1] Department of Food Science and Biotechnology, College of Life Science and Biotechnology, Dongguk University, Goyang-si 10326, Republic of Korea

*** Corresponding author:** Kwang Won Hong (E-mail address: hkwon@dongguk.edu, Tel: +82-31-961-5140, Fax: +82-31-961-5108)

Table S1. Throughput and quality of RNA-seq data.

Sample name[a]	Raw reads	Clean reads	GC content (%)	Q30 (%)[b]	Mapped reads[c]
CON_1	89,326,720	88,475,266	49.54	96.46	87,260,249 (98.63%)
CON_2	76,543,046	67,876,956	46	95.35	73,615,078 (98.62%)
CON_3	66,918,198	78,407,308	46.53	95.72	65,050,378 (98.78%)
WT_1	84,230,456	83,453,836	49.42	96.35	82,452,280 (98.8%)
WT_2	73,224,820	62,388,808	46.68	95.71	71,082,357 (98.67%)
WT_3	68,284,754	58,381,418	46.58	95.28	65,983,072 (98.54%)
G75_1	80,141,736	79,393,794	49.32	96.46	78,472,316 (98.84%)
G75_2	81,607,548	77,436,816	46.46	95.82	76,295,758 (98.53%)
G75_3	87,526,726	74,204,110	47	95.8	73,086,916 (98.49%)

[a] Each sample represents Caco-2 cells unstimulated (CON) or stimulated with wild-type spore (WT) and CotG-p75 (G75), respectively

[b] Q30 indicates the percentage of the sequencing data with an error rate less than 0.1%

[c] Mapped reads represent the reads of clean bases mapped to the reference genome

Table S2. List of differentially expressed genes between wild-type spores stimulated Caco-2 cells and control.

Gene ID	Transcript ID	Gene Symbol	Description	Fold change	Raw *p*-val
84218	NM_032258,NM_032258_dup1,NM_032258_dup2,NM_032258_dup3	*TBC1D3F*	TBC1 domain family member 3F	18.961402	0.000214469
170688	NR_104005	*NUDT4P2*	nudix hydrolase 4 pseudogene 2	18.452825	0.005955287
100534612	NR_037951	*C1QTNF3-AMACR*	C1QTNF3-AMACR readthrough (NMD candidate)	8.032839	4.31517E-05
827	NM_014289	*CAPN6*	calpain 6	7.473258	1.98259E-07
102465252	NR_106761	*MIR6506*	microRNA 6506	6.840195	0.038148197
100271849	NM_001145785	*MEF2B*	myocyte enhancer factor 2B	6.238428	0.000403539
56112	NM_018916,NM_032011	*PCDHGA3*	protocadherin gamma subfamily A, 3	6.093560	0.012781763
11277	NM_007248,NM_016381,NM_033629	*TREX1*	three prime repair exonuclease 1	6.034128	0.010662644
5239	NM_021965	*PGM5*	phosphoglucomutase 5	4.928959	0.006035839
6887	NM_005421	*TAL2*	TAL bHLH transcription factor 2	4.756164	0.031704327
26134	NR_104269	*RALGAPA1P1*	Ral GTPase activating protein catalytic alpha subunit 1 pseudogene 1	4.406933	0.037462804
105377623	NR_136203	*LOC105377623*	uncharacterized LOC105377623	4.318840	0.015946482
3627	NM_001565	*CXCL10*	C-X-C motif chemokine ligand 10	4.314596	2.15252E-06

55351	NM_001306082,NM_001345969,NM_018401	*STK32B*	serine/threonine kinase 32B	3.891382	0.038669644
1558	NM_000770,NM_001198853,NM_001198854,NM_001198855	*CYP2C8*	cytochrome P450 family 2 subfamily C member 8	3.748785	0.022754473
121643	NM_213596	*FOXN4*	forkhead box N4	3.640509	0.002875798
56674	NM_001286094,NM_001286095,NM_020644	*TMEM9B*	TMEM9 domain family member B	3.485800	0.001745819
100532724	NR_037804	*NPHP3-ACAD11*	NPHP3-ACAD11 readthrough (NMD candidate)	3.425432	0.001761894
29075	NR_026883,NR_026884	*LINC00652*	long intergenic non-protein coding RNA 652	3.304828	0.025566796
6844	NM_001330125,NM_014232	*VAMP2*	vesicle associated membrane protein 2	3.292158	0.002847442
340547	NM_001170553,NM_182607	*VSIG1*	V-set and immunoglobulin domain containing 1	3.141374	0.032233996
56963	NM_001166283,NM_001166286,NM_001166287,NM_001166288,NM_001166289,NM_020211	*RGMA*	repulsive guidance molecule family member a	3.060160	0.028147048
9154	NM_001287761,NM_001287762,NM_001321721,NM_001321722,NM_004213,NM_201651	*SLC28A1*	solute carrier family 28 member 1	3.055652	0.026024006
3576	NM_000584	*CXCL8*	C-X-C motif chemokine ligand 8	2.916596	1.09178E-05
2919	NM_001511,NR_046035	*CXCL1*	C-X-C motif chemokine ligand 1	2.871927	6.71872E-07
387804	NM_001144871	*VSTM5*	V-set and transmembrane domain containing 5	2.817476	0.031889685
158158	NM_152573	*RASEF*	RAS and EF-hand domain containing	2.812715	0.014095148

6288	NM_000331,NM_001178006,NM_199161	*SAA1*	serum amyloid A1	2.749324	0.001482969
23166	NM_015136	*STAB1*	stabilin 1	2.638764	0.04040277
2200	NM_000138	*FBN1*	fibrillin 1	2.627290	0.049224873
6364	NM_001130046,NM_004591	*CCL20*	C-C motif chemokine ligand 20	2.614781	1.89372E-40
503569	NR_033932	*RGMB-AS1*	RGMB antisense RNA 1	2.386338	0.030308704
10186	NM_005780	*LHFP*	lipoma HMGIC fusion partner	2.362741	0.04903843
8676	NM_003764	*STX11*	syntaxin 11	2.295532	0.021297684
6289	NM_001127380,NM_030754	*SAA2*	serum amyloid A2	2.286038	0.00012663
1316	NM_001160124,NM_001160125,NM_001300,NR_027653	*KLF6*	Kruppel like factor 6	2.235175	7.46084E-46
64221	NM_022370	*ROBO3*	roundabout guidance receptor 3	2.196472	0.020696525
100507053	NR_037884	*LOC100507053*	uncharacterized LOC100507053	2.184420	0.01692473
6016	NM_001256820,NM_001256821,NM_006912	*RIT1*	Ras like without CAAX 1	2.179966	0.049585676
25804	NM_001252129,NM_012321	*LSM4*	LSM4 homolog, U6 small nuclear RNA and mRNA degradation associated	2.154356	0.004411372
100303453	NR_028393,NR_028394,NR_028395,NR_028396,NR_028397,NR_028398,NR_028399,NR_028400	*TSNAX-DISC1*	TSNAX-DISC1 readthrough (NMD candidate)	2.119292	0.036895458

222663	NM_001303136,NM_152753	*SCUBE3*	signal peptide, CUB domain and EGF like domain containing 3	2.116989	0.029152277
10228	NM_001286210,NM_005819	*STX6*	syntaxin 6	2.106314	0.030699016
9308	NM_001040280,NM_001251901,NM_004233	*CD83*	CD83 molecule	2.098569	0.000919424
6585	NM_003061	*SLIT1*	slit guidance ligand 1	2.074743	0.040270358
146754	NM_001303270,NM_020877	*DNAH2*	dynein axonemal heavy chain 2	2.066093	0.024045255
6752	NM_001050	*SSTR2*	somatostatin receptor 2	2.032396	0.028383156
27071	NM_001306151,NM_014395	*DAPP1*	dual adaptor of phosphotyrosine and 3-phosphoinositides 1	2.025425	0.041354917
643596	NM_001190228	*RNF224*	ring finger protein 224	-2.027034	0.031640547
1727	NM_000398,NM_001129819,NM_001171660,NM_001171661,NM_007326	*CYB5R3*	cytochrome b5 reductase 3	-2.039841	0.026078049
100422558	NR_135312	*SMG1P6*	SMG1P6, nonsense mediated mRNA decay associated PI3K related kinasepseudogene 6	-2.096502	0.000135337
375387	NM_198565	*NRROS*	negative regulator of reactive oxygen species	-2.175426	0.008351408
107080638	NM_001318809,NR_134872	*TBC1D7-LOC100130357*	TBC1D7-LOC100130357 readthrough	-2.252059	0.048645776
3705	NM_001142593,NM_001142594,NM_014216	*ITPK1*	inositol-tetrakisphosphate 1-kinase	-2.368665	0.004564373

107303344	NM_001320727,NR_135324	*SETDB2-PHF11*	SETDB2-PHF11 readthrough	-2.401257	0.043898727
728689	NM_001099661,NM_001099661_dup1,NM_001317856,NM_001317856_dup1,NM_001317857,NM_001317857_dup1	*EIF3CL*	eukaryotic translation initiation factor 3 subunit C like	-2.443269	0.047359397
56961	NM_020209	*SHD*	Src homology 2 domain containing transforming protein D	-2.474844	0.041905817
642402	NR_111962	*GOLGA6L17P*	golgin A6 family-like 17, pseudogene	-2.487276	0.012754051
23491	NM_001185176,NM_001185177,NM_024922	*CES3*	carboxylesterase 3	-2.549066	0.010843784
101929567	NR_110257	*LINC01814*	long intergenic non-protein coding RNA 1814	-2.617591	0.028115297
1656	NM_001257191,NM_004397	*DDX6*	DEAD-box helicase 6	-2.626878	0.007615058
653333	NM_001137610	*FAM86B2*	family with sequence similarity 86 member B2	-2.696774	0.004693836
6495	NM_005982	*SIX1*	SIX homeobox 1	-2.711301	0.046926214
134429	NM_001308056,NM_001308057,NM_001308058,NM_001308059,NM_001308060,NM_001308061,NM_139164,NR_131752,NR_131753,NR_131754	*STARD4*	StAR related lipid transfer domain containing 4	-2.728938	0.010090297
201501	NM_001039360,NM_001318841	*ZBTB7C*	zinc finger and BTB domain containing 7C	-2.879693	0.02065907

7781	NM_001318949,NM_001318950,NM_001318951,NM_003459	*SLC30A3*	solute carrier family 30 member 3	-3.000219	0.035474973
407975	NR_027349,NR_027350	*MIR17HG*	miR-17-92a-1 cluster host gene	-3.247756	0.004824816
55073	NR_002940	*LRRC37A4P*	leucine rich repeat containing 37 member A4, pseudogene	-3.489345	0.005117573
100131561	NR_024013	*FKSG29*	FKSG29	-3.698458	0.041046714
197196	NR_038859	*LINC00311*	long intergenic non-protein coding RNA 311	-4.103307	0.02243765
347694	NR_028501	*ECEL1P2*	endothelin converting enzyme like 1 pseudogene 2	-4.197553	0.025469768
100861412	NM_001256141	*FSBP*	fibrinogen silencer binding protein	-4.239391	0.01869087
5555	NM_001110213	*PRH2*	proline rich protein HaeIII subfamily 2	-4.438711	0.028546589
257044	NM_001130957,NM_001242340,NM_173807	*CATSPERE*	catsper channel auxiliary subunit epsilon	-4.460617	0.022646154
9073	NM_199328	*CLDN8*	claudin 8	-5.748759	0.040771417
729873	NM_001123391,NM_001123391_dup1	*TBC1D3*	TBC1 domain family member 3	-5.889728	0.040851376
100137049	NM_001114633	*PLA2G4B*	phospholipase A2 group IVB	-6.684008	0.044529946
100616394	NR_039646_chr2,NR_039646_chr3	*MIR4444-1*	microRNA 4444-1	-6.951703	0.002232175
100533179	NR_037904	*UBE2F-SCLY*	UBE2F-SCLY readthrough (NMD candidate)	-7.585978	8.94368E-06

100631383	NM_001242939	*FAM47E-STBD1*	FAM47E-STBD1 readthrough	-7.799596	0.047410174
140564	NM_152426	*APOBEC3D*	apolipoprotein B mRNA editing enzyme catalytic subunit 3D	-8.372159	2.7395E-10
54065	NM_058182	*SMIM11A*	small integral membrane protein 11A	-10.725595	0.015284053
474384	NM_001007524,NM_001007524_dup1,NM_001007524_dup2	*F8A3*	coagulation factor VIII-associated 3	-21.111247	0.006049599
100846999	NR_049831_chr2,NR_049831_chr3	*MIR4444-2*	microRNA 4444-2	-23.016168	6.25565E-05

Table S3. List of differentially expressed genes between CotG-p75 stimulated Caco-2 cells and control.

Gene ID	Transcript ID	Gene Symbol	Description	Fold change	Raw *p*-val
3627	NM_001565	*CXCL10*	C-X-C motif chemokine ligand 10	35.584875	5.23266E-34
6364	NM_001130046,NM_004591	*CCL20*	C-C motif chemokine ligand 20	21.176225	0
101060321	NM_001291462,NM_001291462_dup1,NM_001291462_dup2, NM_001291462_dup3	*TBC1D3G*	TBC1 domain family member 3G	20.731774	0.014788059
3576	NM_000584	*CXCL8*	C-X-C motif chemokine ligand 8	20.169180	4.15023E-38
2919	NM_001511,NR_046035	*CXCL1*	C-X-C motif chemokine ligand 1	16.814684	9.55903E-41
100534612	NR_037951	*C1QTNF3-AMACR*	C1QTNF3-AMACR readthrough (NMD candidate)	14.844345	1.01011E-07
84218	NM_032258,NM_032258_dup1,NM_032258_dup2,NM_032258_dup3	*TBC1D3F*	TBC1 domain family member 3F	12.517674	0.000857422
330	NM_001165,NM_182962	*BIRC3*	baculoviral IAP repeat containing 3	11.904057	3.7596E-218
1543	NM_000499,NM_001319216,NM_001319217	*CYP1A1*	cytochrome P450 family 1 subfamily A member 1	11.796821	0
2770	NM_001256414,NM_002069	*GNAI1*	G protein subunit alpha i1	11.539755	1.29568E-10
2921	NM_002090	*CXCL3*	C-X-C motif chemokine ligand 3	8.098634	3.92626E-52
157739	NR_001578	*TDH*	L-threonine dehydrogenase (pseudogene)	7.274196	1.69932E-05
6288	NM_000331,NM_001178006,NM_199161	*SAA1*	serum amyloid A1	6.837343	6.48104E-10

9308	NM_001040280,NM_001251901,NM_004233	*CD83*	CD83 molecule	6.746527	5.46459E-19
8676	NM_003764	*STX11*	syntaxin 11	6.591671	5.99685E-08
121643	NM_213596	*FOXN4*	forkhead box N4	6.589470	9.81955E-06
1545	NM_000104	*CYP1B1*	cytochrome P450 family 1 subfamily B member 1	6.556214	1.76862E-64
100533970	NR_037928	*P2RX5-TAX1BP3*	P2RX5-TAX1BP3 readthrough (NMD candidate)	6.293272	0.001128609
6373	NM_001302123,NM_005409	*CXCL11*	C-X-C motif chemokine ligand 11	5.913225	0.002376425
8862	NM_017413	*APLN*	apelin	5.830446	7.18733E-23
25976	NM_001184717,NM_001184718,NM_015508	*TIPARP*	TCDD inducible poly(ADP-ribose) polymerase	5.819714	4.2338E-198
3604	NM_001561	*TNFRSF9*	TNF receptor superfamily member 9	5.761583	1.07026E-09
89884	NM_033343	*LHX4*	LIM homeobox 4	5.679677	0.000588542
2920	NM_002089	*CXCL2*	C-X-C motif chemokine ligand 2	5.592997	6.66292E-41
6376	NM_001304392,NM_002996	*CX3CL1*	C-X3-C motif chemokine ligand 1	5.539204	9.44953E-09
100533179	NR_037904	*UBE2F-SCLY*	UBE2F-SCLY readthrough (NMD candidate)	5.483671	1.21629E-06
64386	NM_022468	*MMP25*	matrix metallopeptidase 25	5.431275	0.000200914
5328	NM_001145031,NM_001319191,NM_002658	*PLAU*	plasminogen activator, urokinase	5.361074	8.87672E-22
7039	NM_001099691,NM_00130815	*TGFA*	transforming growth factor alpha	5.055470	2.06805E-09

	8,NM_001308159,NM_003236				
56892	NM_020130	*C8orf4*	chromosome 8 open reading frame 4	5.042263	3.56535E-12
8792	NM_001270949,NM_001270950,NM_001270951,NM_001278268,NM_003839	*TNFRSF11A*	TNF receptor superfamily member 11a	4.977152	3.77375E-07
4153	NM_000242	*MBL2*	mannose binding lectin 2	4.976853	1.34404E-27
374378	NM_198516	*GALNT18*	polypeptide N-acetylgalactosaminyltransferase 18	4.964477	0.040497936
56112	NM_018916,NM_032011	*PCDHGA3*	protocadherin gamma subfamily A, 3	4.933845	0.029519018
6289	NM_001127380,NM_030754	*SAA2*	serum amyloid A2	4.931657	3.35841E-14
130367	NM_001320833,NM_001320834,NM_152386	*SGPP2*	sphingosine-1-phosphate phosphatase 2	4.923502	8.32138E-11
1435	NM_000757,NM_172210,NM_172211,NM_172212	*CSF1*	colony stimulating factor 1	4.922399	1.23174E-25
6752	NM_001050	*SSTR2*	somatostatin receptor 2	4.881280	5.64005E-07
30817	NM_001271052,NM_013447	*ADGRE2*	adhesion G protein-coupled receptor E2	4.764381	0.00032501
100271849	NM_001145785	*MEF2B*	myocyte enhancer factor 2B	4.762315	0.002738664
10186	NM_005780	*LHFP*	lipoma HMGIC fusion partner	4.609391	0.000381915
6091	NM_001145845,NM_002941,NM_133631	*ROBO1*	roundabout guidance receptor 1	4.573408	0.000934938
8444	NM_001004023,NM_003582	*DYRK3*	dual specificity tyrosine phosphorylation regulated kinase 3	4.516067	0.000579584

7477	NM_058238	*WNT7B*	Wnt family member 7B	4.372204	5.76985E-07
125488	NM_001135993,NM_001243425,NM_001292030,NM_153211	*TTC39C*	tetratricopeptide repeat domain 39C	4.295301	1.28414E-23
100129129	NR_147705	*LOC100129129*	uncharacterized LOC100129129	4.281870	3.23768E-05
9751	NM_001318234,NM_014723	*SNPH*	syntaphilin	4.168258	0.002091957
57214	NM_001293298,NM_001293304,NM_018689	*CEMIP*	cell migration inducing hyaluronan binding protein	4.142986	3.85051E-08
3754	NM_002236	*KCNF1*	potassium voltage-gated channel modifier subfamily F member 1	3.995637	1.53752E-06
5239	NM_021965	*PGM5*	phosphoglucomutase 5	3.987819	0.017809806
115811	NM_001330452,NM_138451	*IQCD*	IQ motif containing D	3.971746	7.20386E-06
827	NM_014289	*CAPN6*	calpain 6	3.954691	0.000483719
10170	NM_001142270,NM_001142271,NM_001289763,NM_199204	*DHRS9*	dehydrogenase/reductase 9	3.912342	9.16896E-09
5054	NM_000602	*SERPINE1*	serpin family E member 1	3.911712	1.2573E-09
203190	NM_139278	*LGI3*	leucine rich repeat LGI family member 3	3.897524	0.035218552
135112	NM_001122842,NM_001199619,NM_001199620,NM_001199621,NM_001199622,NM_181782	*NCOA7*	nuclear receptor coactivator 7	3.757528	5.69906E-96
222611	NM_153839	*ADGRF2*	adhesion G protein-coupled receptor F2	3.664996	4.1548E-52
146	NM_000678	*ADRA1D*	adrenoceptor alpha 1D	3.656734	3.14772E-06

27289	NM_014470	*RND1*	Rho family GTPase 1	3.613103	1.13986E-26
9283	NM_004767	*GPR37L1*	G protein-coupled receptor 37 like 1	3.605473	8.0249E-11
135398	NM_001145652,NR_146853,NR_146854,NR_146855,NR_146856,NR_146857,NR_146858,NR_146859,NR_146860,NR_146861,NR_146862,NR_146863,NR_146864	*C6orf141*	chromosome 6 open reading frame 141	3.583108	4.27101E-16
12	NM_001085	*SERPINA3*	serpin family A member 3	3.543268	2.79391E-11
100240734	NR_026657	*LOC100240734*	uncharacterized LOC100240734	3.541523	0.04201445
55289	NM_001142807	*ACOXL*	acyl-CoA oxidase like	3.463005	0.018587251
100532726	NM_001203260,NM_001203261,NM_001203262	*NDUFC2-KCTD14*	NDUFC2-KCTD14 readthrough	3.455603	0.039983705
7474	NM_001256105,NM_003392	*WNT5A*	Wnt family member 5A	3.451416	1.03966E-05
54658	NM_000463	*UGT1A1*	UDP glucuronosyltransferase family 1 member A1	3.440544	2.12265E-20
7128	NM_001270507,NM_001270508,NM_006290	*TNFAIP3*	TNF alpha induced protein 3	3.438507	7.7077E-119
145837	NR_026979	*DRAIC*	downregulated RNA in cancer, inhibitor of cell invasion and migration	3.417678	4.04033E-07
100132234	NR_135254	*LINC00543*	long intergenic non-protein coding RNA 543	3.411526	4.13294E-50
574029	NR_002834	*DUSP5P1*	dual specificity phosphatase 5 pseudogene 1	3.409071	4.24515E-07
3553	NM_000576	*IL1B*	interleukin 1 beta	3.382881	0.000206638

3656	NM_001570	*IRAK2*	interleukin 1 receptor associated kinase 2	3.363667	1.77017E-63
5629	NM_001270616,NM_002763	*PROX1*	prospero homeobox 1	3.331543	6.28211E-81
1958	NM_001964	*EGR1*	early growth response 1	3.288240	4.55934E-10
57822	NM_001195010,NM_021180,NM_198173,NM_198174	*GRHL3*	grainyhead like transcription factor 3	3.280235	8.91267E-17
23025	NM_001080421	*UNC13A*	unc-13 homolog A	3.274603	0.001889476
100303453	NR_028393,NR_028394,NR_028395,NR_028396,NR_028397,NR_028398,NR_028399,NR_028400	*TSNAX-DISC1*	TSNAX-DISC1 readthrough (NMD candidate)	3.208131	0.001016712
135644	NM_001286633,NM_138700	*TRIM40*	tripartite motif containing 40	3.203136	0.00060256
7475	NM_006522	*WNT6*	Wnt family member 6	3.161065	0.01061564
339122	NM_001204883,NM_001204884,NM_001204885,NM_001204886,NM_001204887,NM_001204888,NM_198490	*RAB43*	RAB43, member RAS oncogene family	3.141010	0.019466655
10537	NM_006398	*UBD*	ubiquitin D	3.115467	2.42059E-28
3484	NM_000596	*IGFBP1*	insulin like growth factor binding protein 1	3.106487	2.72499E-05
23764	NM_001161572,NM_001161573,NM_001161574,NM_012323	*MAFF*	MAF bZIP transcription factor F	3.070828	5.1732E-43
6844	NM_001330125,NM_014232	*VAMP2*	vesicle associated membrane protein 2	3.056283	0.005234499
4973	NM_001172632,NM_001172633,NM_002543	*OLR1*	oxidized low density lipoprotein receptor 1	3.048117	2.01571E-44

72	NM_001199893,NM_001615	*ACTG2*	actin, gamma 2, smooth muscle, enteric	3.031186	0.031869792
9982	NM_005130	*FGFBP1*	fibroblast growth factor binding protein 1	3.029162	3.00845E-27
340547	NM_001170553,NM_182607	*VSIG1*	V-set and immunoglobulin domain containing 1	3.022906	0.038373413
1846	NM_001394,NM_057158	*DUSP4*	dual specificity phosphatase 4	2.978551	4.36635E-51
4884	NM_002522	*NPTX1*	neuronal pentraxin 1	2.938715	2.7988E-07
440823	NR_003491,NR_033319,NR_03 3320,NR_033321	*MIAT*	myocardial infarction associated transcript (non-protein coding)	2.915714	0.018188129
2571	NM_000817,NM_013445	*GAD1*	glutamate decarboxylase 1	2.865318	1.03802E-30
340152	NM_207360	*ZC3H12D*	zinc finger CCCH-type containing 12D	2.848948	1.64121E-13
6585	NM_003061	*SLIT1*	slit guidance ligand 1	2.826775	0.003011963
5971	NM_006509	*RELB*	RELB proto-oncogene, NF-kB subunit	2.825588	1.63064E-35
389384	NM_001010903	*C6orf222*	chromosome 6 open reading frame 222	2.823306	1.62083E-43
55691	NM_001318336,NM_00131833 7,NM_001318338,NM_018027, NR_134578	*FRMD4A*	FERM domain containing 4A	2.817286	8.55342E-51
93082	NM_001285485,NM_00128548 6,NR_104346	*NEURL3*	neuralized E3 ubiquitin protein ligase 3	2.815877	1.28475E-16
4792	NM_020529	*NFKBIA*	NFKB inhibitor alpha	2.799798	1.67293E-75
92610	NM_052864	*TIFA*	TRAF interacting protein with forkhead associated domain	2.769515	6.44013E-26
56963	NM_001166283,NM_00116628	*RGMA*	repulsive guidance molecule family member a	2.765340	0.046018308

	6,NM_001166287,NM_001166288,NM_001166289,NM_020211				
6654	NM_005633	*SOS1*	SOS Ras/Rac guanine nucleotide exchange factor 1	2.747714	7.75898E-57
161742	NM_152594	*SPRED1*	sprouty related EVH1 domain containing 1	2.746786	3.61852E-30
120939	NM_001079815,NM_153022	*TMEM52B*	transmembrane protein 52B	2.746062	1.15258E-20
8843	NM_006018	*HCAR3*	hydroxycarboxylic acid receptor 3	2.744520	0.01136265
8870	NM_003897	*IER3*	immediate early response 3	2.742145	1.56987E-33
4879	NM_002521	*NPPB*	natriuretic peptide B	2.728006	2.77574E-05
27071	NM_001306151,NM_014395	*DAPP1*	dual adaptor of phosphotyrosine and 3-phosphoinositides 1	2.715616	0.003840461
25804	NM_001252129,NM_012321	*LSM4*	LSM4 homolog, U6 small nuclear RNA and mRNA degradation associated	2.681507	0.000259124
142680	NM_001177316,NM_001177317,NM_080877	*SLC34A3*	solute carrier family 34 member 3	2.675429	0.02645684
9641	NM_001193321,NM_001193322,NM_014002	*IKBKE*	inhibitor of nuclear factor kappa B kinase subunit epsilon	2.673090	5.33582E-33
3872	NM_000422	*KRT17*	keratin 17	2.668686	2.94103E-12
85462	NM_033393	*FHDC1*	FH2 domain containing 1	2.646834	4.80859E-15
220	NM_000693,NM_001293815	*ALDH1A3*	aldehyde dehydrogenase 1 family member A3	2.591297	5.60411E-11
56674	NM_001286094,NM_001286095,NM_020644	*TMEM9B*	TMEM9 domain family member B	2.587722	0.017354399

22808	NM_001085049,NM_001252090,NM_001252091,NM_001252092,NM_001252093,NM_012219	*MRAS*	muscle RAS oncogene homolog	2.583297	6.73807E-07
102724084	NR_120307	*LOC102724084*	uncharacterized LOC102724084	2.576796	0.000334353
6367	NM_002990	*CCL22*	C-C motif chemokine ligand 22	2.572942	0.047783245
685	NM_001316963,NM_001729	*BTC*	betacellulin	2.572600	0.000207781
338442	NM_177551	*HCAR2*	hydroxycarboxylic acid receptor 2	2.562473	0.020853273
51284	NM_016562	*TLR7*	toll like receptor 7	2.555552	0.004359017
65989	NM_001286655,NM_001286656,NM_023932,NM_206539	*DLK2*	delta like non-canonical Notch ligand 2	2.553783	0.008280256
84847	NR_038407	*LINC00525*	long intergenic non-protein coding RNA 525	2.552726	0.029880052
7127	NM_006291	*TNFAIP2*	TNF alpha induced protein 2	2.551546	7.04546E-13
405753	NM_207581	*DUOXA2*	dual oxidase maturation factor 2	2.540025	1.44822E-06
29947	NM_013369,NM_175867	*DNMT3L*	DNA methyltransferase 3 like	2.539188	0.008752878
138065	NM_145051	*RNF183*	ring finger protein 183	2.531477	0.000909853
158158	NM_152573	*RASEF*	RAS and EF-hand domain containing	2.524432	0.029070882
8538	NM_003658	*BARX2*	BARX homeobox 2	2.523899	0.002024079
54873	NM_017734	*PALMD*	palmdelphin	2.512094	0.001187407
100133205	NR_026775	*LINC00240*	long intergenic non-protein coding RNA 240	2.511926	0.032061195

80149	NM_001323550,NM_001323551,NM_025079	*ZC3H12A*	zinc finger CCCH-type containing 12A	2.509189	2.18643E-25
647033	NR_003284	*PA2G4P4*	proliferation-associated 2G4 pseudogene 4	2.503145	7.32513E-06
3934	NM_005564	*LCN2*	lipocalin 2	2.499087	0.021412644
23034	NM_001161576,NM_001161577,NM_015589	*SAMD4A*	sterile alpha motif domain containing 4A	2.470770	0.004310303
25780	NM_001139488,NM_001349975,NM_001349976,NM_001349977,NM_001349978,NM_001349979,NM_001349980,NM_001349981,NM_015376,NM_170672	*RASGRP3*	RAS guanyl releasing protein 3	2.466611	5.89717E-13
9953	NM_006041,NR_130138	*HS3ST3B1*	heparan sulfate-glucosamine 3-sulfotransferase 3B1	2.440158	0.008551736
100499467	NR_036488,NR_137280,NR_137281	*LINC00673*	long intergenic non-protein coding RNA 673	2.437728	2.24695E-34
3708	NM_001099952,NM_001168272,NM_002222	*ITPR1*	inositol 1,4,5-trisphosphate receptor type 1	2.435095	1.0744E-09
5055	NM_001143818,NM_002575	*SERPINB2*	serpin family B member 2	2.432125	0.019790527
64150	NR_002770	*DIO3OS*	DIO3 opposite strand/antisense RNA (head to head)	2.431530	1.70251E-06
401022	NR_033979,NR_110458,NR_110459,NR_110460,NR_110461,NR_110462,NR_110463,NR_110464,NR_110465,NR_110466,N	*HAGLR*	HOXD antisense growth-associated long non-coding RNA	2.430127	0.002512305

	R_110467,NR_110468				
23120	NM_025153	*ATP10B*	ATPase phospholipid transporting 10B (putative)	2.422210	5.81828E-21
6776	NM_001288718,NM_001288719,NM_001288720,NM_003152	*STAT5A*	signal transducer and activator of transcription 5A	2.420622	2.46602E-13
84807	NM_001321831,NM_139239	*NFKBID*	NFKB inhibitor delta	2.409030	2.23266E-08
85027	NM_032947	*SMIM3*	small integral membrane protein 3	2.407382	5.64489E-36
1959	NM_000399,NM_001136177,NM_001136178,NM_001136179,NM_001321037	*EGR2*	early growth response 2	2.406854	0.011301735
100529063	NM_001199864	*BCL2L2-PABPN1*	BCL2L2-PABPN1 readthrough	2.404173	0.035095724
131601	NM_001136053,NM_001142646,NR_073377	*TPRA1*	transmembrane protein adipocyte associated 1	2.396376	3.13055E-46
9429	NM_001257386,NM_001348985,NM_001348986,NM_001348987,NM_001348988,NM_001348989,NM_004827	*ABCG2*	ATP binding cassette subfamily G member 2 (Junior blood group)	2.393271	6.76119E-30
389170	NM_001004316,NM_001193283	*LEKR1*	leucine, glutamate and lysine rich 1	2.383712	0.014398694
6274	NM_002960	*S100A3*	S100 calcium binding protein A3	2.380431	0.035823746
4843	NM_000625	*NOS2*	nitric oxide synthase 2	2.379397	3.99473E-30
149465	NM_001167965,NM_001195831,NM_152498	*CFAP57*	cilia and flagella associated protein 57	2.377490	0.018565826
105372833	NR_135514	*LOC105372833*	uncharacterized LOC105372833	2.376260	1.44974E-05

245972	NM_152565	*ATP6V0D2*	ATPase H+ transporting V0 subunit d2	2.373291	4.75498E-10
2353	NM_005252	*FOS*	Fos proto-oncogene, AP-1 transcription factor subunit	2.360830	6.23805E-23
54997	NM_001168325,NM_017899,NR_031766	*TESC*	tescalcin	2.360748	5.31983E-06
1009	NM_001308392,NM_001330576,NM_001797	*CDH11*	cadherin 11	2.358787	2.17383E-05
6004	NM_002928	*RGS16*	regulator of G protein signaling 16	2.345610	0.000347529
8061	NM_001300844,NM_001300855,NM_001300856,NM_001300857,NM_005438,NR_125339	*FOSL1*	FOS like 1, AP-1 transcription factor subunit	2.343730	4.92036E-11
1827	NM_001285389,NM_001285391,NM_001285392,NM_001285393,NM_001331016,NM_004414,NM_203417,NM_203418	*RCAN1*	regulator of calcineurin 1	2.342425	6.82493E-49
64221	NM_022370	*ROBO3*	roundabout guidance receptor 3	2.333417	0.012529228
104472715	NR_146177	*SNHG14*	small nucleolar RNA host gene 14	2.332958	0.000161021
319	NM_001638	*APOF*	apolipoprotein F	2.330319	0.047162013
8140	NM_003486	*SLC7A5*	solute carrier family 7 member 5	2.322804	7.18676E-84
374	NM_001657,NM_001657_dup1	*AREG*	amphiregulin	2.314708	4.37814E-39
54757	NM_001243746,NM_017565,NR_027751	*FAM20A*	FAM20A, golgi associated secretory pathway pseudokinase	2.305631	9.86224E-17
4698	NM_001282419,NM_00128242	*NDUFA5*	NADH:ubiquinone oxidoreductase subunit A5	2.301338	0.034929114

	0,NM_001282421,NM_001282422,NM_001291304,NM_005000,NR_104168,NR_104169,NR_111925,NR_111926				
57538	NM_020778	*ALPK3*	alpha kinase 3	2.300113	8.51431E-05
57491	NM_001242412,NM_020731	*AHRR*	aryl-hydrocarbon receptor repressor	2.290064	1.3321E-05
80117	NM_025047	*ARL14*	ADP ribosylation factor like GTPase 14	2.286307	2.81805E-15
84239	NM_032279	*ATP13A4*	ATPase 13A4	2.282577	0.004833661
2172	NM_001040442,NM_001130958,NM_001445	*FABP6*	fatty acid binding protein 6	2.255570	0.014950897
22806	NM_001257408,NM_001257409,NM_001257410,NM_001257411,NM_001257412,NM_001257413,NM_001257414,NM_001284514,NM_001284515,NM_001284516,NM_012481,NM_183228,NM_183229,NM_183230,NM_183231,NM_183232	*IKZF3*	IKAROS family zinc finger 3	2.244544	0.002804071
3383	NM_000201	*ICAM1*	intercellular adhesion molecule 1	2.240086	9.38044E-43
83697	NM_001258426,NM_001258427,NM_001258428,NM_031467	*SLC4A9*	solute carrier family 4 member 9	2.228717	0.03009497
6036	NM_002934	*RNASE2*	ribonuclease A family member 2	2.222510	0.00024295
5581	NM_005400	*PRKCE*	protein kinase C epsilon	2.212503	4.73436E-08
29995	NM_001278233,NM_00127823	*LMCD1*	LIM and cysteine rich domains 1	2.212136	8.6856E-16

	4,NM_001278235,NM_014583				
25841	NM_145804	*ABTB2*	ankyrin repeat and BTB domain containing 2	2.203196	1.03409E-30
767	NM_001321837,NM_001321838,NM_001321839,NM_004056,NR_135821	*CA8*	carbonic anhydrase 8	2.198790	7.11158E-10
54361	NM_030761	*WNT4*	Wnt family member 4	2.194233	0.000172976
602	NM_005178	*BCL3*	B-cell CLL/lymphoma 3	2.190791	6.44962E-35
23308	NM_001283050,NM_001283051,NM_001283052,NM_015259	*ICOSLG*	inducible T-cell costimulator ligand	2.187852	1.49475E-29
80153	NM_001142443,NM_001142444,NM_001351378,NM_001351379,NM_025083	*EDC3*	enhancer of mRNA decapping 3	2.176841	6.68105E-61
1848	NM_001946,NM_022652	*DUSP6*	dual specificity phosphatase 6	2.168106	3.27558E-44
1839	NM_001945	*HBEGF*	heparin binding EGF like growth factor	2.157453	5.42064E-14
55022	NM_001100818,NM_001330156,NM_001330157,NM_001330158,NM_017933	*PID1*	phosphotyrosine interaction domain containing 1	2.147579	0.044422635
6330	NM_001142348,NM_001142349,NM_174934,NR_024527	*SCN4B*	sodium voltage-gated channel beta subunit 4	2.143185	0.000213696
2113	NM_001143820,NM_001162422,NM_001330451,NM_005238	*ETS1*	ETS proto-oncogene 1, transcription factor	2.136900	1.10592E-09
91768	NM_001100619,NM_001256438,NM_138375,NR_023359	*CABLES1*	Cdk5 and Abl enzyme substrate 1	2.132428	1.30246E-39

1235	NM_004367,NM_031409	*CCR6*	C-C motif chemokine receptor 6	2.127072	0.000380748
22822	NM_007350	*PHLDA1*	pleckstrin homology like domain family A member 1	2.124608	1.45448E-27
9506	NM_001318877,NM_007003	*PAGE4*	PAGE family member 4	2.122817	2.09136E-05
10228	NM_001286210,NM_005819	*STX6*	syntaxin 6	2.117213	0.029830883
3659	NM_002198	*IRF1*	interferon regulatory factor 1	2.111544	7.13336E-26
30811	NM_014586	*HUNK*	hormonally up-regulated Neu-associated kinase	2.111345	1.53856E-09
1410	NM_001289807,NM_001289808,NM_001330379,NM_001885	*CRYAB*	crystallin alpha B	2.107352	0.002054252
4791	NM_001077494,NM_001261403,NM_001288724,NM_001322934,NM_001322935,NM_002502	*NFKB2*	nuclear factor kappa B subunit 2	2.090694	3.58852E-37
2706	NM_004004	*GJB2*	gap junction protein beta 2	2.088922	6.18675E-07
10263	NM_001271849,NM_005851,NR_073484	*CDK2AP2*	cyclin dependent kinase 2 associated protein 2	2.075197	2.99635E-49
5021	NM_000916	*OXTR*	oxytocin receptor	2.074052	6.65368E-05
84959	NM_032873	*UBASH3B*	ubiquitin associated and SH3 domain containing B	2.071488	9.09189E-18
79827	NM_024769	*CLMP*	CXADR like membrane protein	2.064487	0.030992872
57210	NM_001080431,NM_001286646,NM_001286648	*SLC45A4*	solute carrier family 45 member 4	2.055459	0.000618996

11182	NM_001145099,NM_017585	*SLC2A6*	solute carrier family 2 member 6	2.053969	1.76872E-14
768239	NM_001085382	*PSAPL1*	prosaposin-like 1 (gene/pseudogene)	2.053785	2.51527E-23
218	NM_000691,NM_001135167,NM_001135168,NM_001330150	*ALDH3A1*	aldehyde dehydrogenase 3 family member A1	2.051472	0.000375005
55521	NM_001017397,NM_001017398,NM_001300752,NM_001300759,NM_018700	*TRIM36*	tripartite motif containing 36	2.051419	0.010848952
6898	NM_000353	*TAT*	tyrosine aminotransferase	2.050106	0.012625027
2254	NM_002010	*FGF9*	fibroblast growth factor 9	2.048731	0.001481173
116496	NM_052966	*FAM129A*	family with sequence similarity 129 member A	2.023770	0.020192992
653464	NM_001271872,NM_001329984	*SRGAP2C*	SLIT-ROBO Rho GTPase activating protein 2C	2.015556	0.002032153
220108	NM_001242312,NM_001330522,NM_145019	*FAM124A*	family with sequence similarity 124 member A	2.006403	0.000835411
79616	NM_001308173,NM_024565,NR_131769	*CCNJL*	cyclin J like	2.005526	2.20895E-39
4208	NM_001131005,NM_001193347,NM_001193348,NM_001193349,NM_001193350,NM_001308002,NM_002397	*MEF2C*	myocyte enhancer factor 2C	2.004788	0.002499408
284161	NM_001165993,NM_001165994,NM_182569	*GDPD1*	glycerophosphodiester phosphodiesterase domain containing 1	-2.005585	0.01303733
6856	NM_006754,NM_182715	*SYPL1*	synaptophysin like 1	-2.056184	0.042184811

375616	NM_001135914,NM_199349	*KCP*	kielin/chordin-like protein	-2.068429	0.029764903
6638	NM_001349454,NM_001349455,NM_001349456,NM_001349457,NM_001349458,NM_001349459,NM_001349460,NM_001349461,NM_001349462,NM_001349463,NM_001349464,NM_001349465,NM_003097,NM_022805,NM_022806,NM_022807,NM_022808	*SNRPN*	small nuclear ribonucleoprotein polypeptide N	-2.078321	0.005664251
81031	NM_030777	*SLC2A10*	solute carrier family 2 member 10	-2.138168	0.046967119
101928865	NR_120564	*LOC101928865*	uncharacterized LOC101928865	-2.168415	0.046498353
11248	NM_007225	*NXPH3*	neurexophilin 3	-2.173053	0.000406926
10451	NM_001079874,NM_006113	*VAV3*	vav guanine nucleotide exchange factor 3	-2.190234	2.46399E-12
107985781	NR_144453,NR_144454	*FSIP2-AS1*	FSIP2 antisense RNA 1	-2.261266	0.030835302
644714	NR_033947	*LIMD1-AS1*	LIMD1 antisense RNA 1	-2.301933	0.010878452
1903	NM_005226	*S1PR3*	sphingosine-1-phosphate receptor 3	-2.351274	0.008415378
25903	NM_001297713,NM_001347700,NM_015441	*OLFML2B*	olfactomedin like 2B	-2.410259	0.016652944
7277	NM_001278552,NM_006000	*TUBA4A*	tubulin alpha 4a	-2.429044	0.015382476
148418	NM_001010971,NM_001134663,NM_001134664	*SAMD13*	sterile alpha motif domain containing 13	-2.447818	0.039957097
169841	NM_001301275,NM_003448,N	*ZNF169*	zinc finger protein 169	-2.565838	6.76084E-06

	M_194320				
29993	NM_001199583,NM_020804	*PACSIN1*	protein kinase C and casein kinase substrate in neurons 1	-2.605199	0.029939416
727896	NR_026659	*LOC727896*	cysteine and histidine rich domain containing 1 pseudogene	-2.625021	0.00141677
107080638	NM_001318809,NR_134872	*TBC1D7-LOC100130357*	TBC1D7-LOC100130357 readthrough	-2.753368	0.017207475
100526832	NM_001199290,NR_144936	*PHOSPHO2-KLHL23*	PHOSPHO2-KLHL23 readthrough	-2.806763	0.038904849
101927157	NR_125879	*LOC101927157*	uncharacterized LOC101927157	-2.845245	0.025244643
56256	NM_019605	*SERTAD4*	SERTA domain containing 4	-2.965962	0.01552691
1795	NM_004947	*DOCK3*	dedicator of cytokinesis 3	-2.967732	0.035192958
101929140	NR_120423	*LOC101929140*	uncharacterized LOC101929140	-3.034141	0.046480615
90586	NR_002773	*AOC4P*	amine oxidase, copper containing 4, pseudogene	-3.055845	0.012367669
4622	NM_017533	*MYH4*	myosin heavy chain 4	-3.088172	0.034090663
4345	NM_001004196,NM_001318826,NM_001318828,NM_001318830,NM_005944	*CD200*	CD200 molecule	-3.152908	0.012684179
100506084	NM_001039083,NM_001103154,NM_001330240,NM_001330240_dup1,NM_001330240_dup2	*ARL17B*	ADP ribosylation factor like GTPase 17B	-3.913194	3.25137E-08
100861412	NM_001256141	*FSBP*	fibrinogen silencer binding protein	-4.049103	0.022774495

3910	NM_001105206,NM_001105207,NM_001105208,NM_001105209,NM_002290	*LAMA4*	laminin subunit alpha 4	-4.063789	0.009381453
84694	NM_032602	*GJA10*	gap junction protein alpha 10	-4.404354	0.016114064
407975	NR_027349,NR_027350	*MIR17HG*	miR-17-92a-1 cluster host gene	-5.776073	2.86063E-05
100526767	NM_001198954	*RNF103-CHMP3*	RNF103-CHMP3 readthrough	-7.685068	1.50443E-05
140564	NM_152426	*APOBEC3D*	apolipoprotein B mRNA editing enzyme catalytic subunit 3D	-10.607301	7.10005E-12
54065	NM_058182	*SMIM11A*	small integral membrane protein 11A	-13.233948	0.008295283
100616394	NR_039646_chr2,NR_039646_chr3	*MIR4444-1*	microRNA 4444-1	-62.447699	2.97884E-10
100846999	NR_049831_chr2,NR_049831_chr3	*MIR4444-2*	microRNA 4444-2	-90.594034	3.49878E-07

Table S4. List of differentially expressed genes between CotG-p75-stimulated and wild-type spore-stimulated Caco-2 cells.

Gene ID	Transcript ID	Gene Symbol	Description	Fold change	Raw *p*-val
100533179	NR_037904	*UBE2F-SCLY*	UBE2F-SCLY readthrough (NMD candidate)	41.599007	2.23091E-17
1543	NM_000499,NM_001319216,NM_001319217	*CYP1A1*	cytochrome P450 family 1 subfamily A member 1	8.592991	0
3627	NM_001565	*CXCL10*	C-X-C motif chemokine ligand 10	8.247557	1.73586E-25
6364	NM_001130046,NM_004591	*CCL20*	C-C motif chemokine ligand 20	8.098661	1.3334E-269
8444	NM_001004023,NM_003582	*DYRK3*	dual specificity tyrosine phosphorylation regulated kinase 3	6.953617	2.50561E-06
3576	NM_000584	*CXCL8*	C-X-C motif chemokine ligand 8	6.915316	3.94676E-26
1545	NM_000104	*CYP1B1*	cytochrome P450 family 1 subfamily B member 1	6.511592	6.0171E-79
100137049	NM_001114633	*PLA2G4B*	phospholipase A2 group IVB	6.120328	0.031872324
330	NM_001165,NM_182962	*BIRC3*	baculoviral IAP repeat containing 3	6.093163	7.4151E-151
2919	NM_001511,NR_046035	*CXCL1*	C-X-C motif chemokine ligand 1	5.854843	2.64117E-21
2770	NM_001256414,NM_002069	*GNAI1*	G protein subunit alpha i1	5.726312	2.30508E-07
25976	NM_001184717,NM_001184718,NM_015508	*TIPARP*	TCDD inducible poly(ADP-ribose) polymerase	5.684022	6.8335E-238
100129129	NR_147705	*LOC100129129*	uncharacterized LOC100129129	5.631024	5.96922E-08
146	NM_000678	*ADRA1D*	adrenoceptor alpha 1D	5.380459	6.78846E-11
2921	NM_002090	*CXCL3*	C-X-C motif chemokine ligand 3	5.145310	3.67022E-42

9751	NM_001318234,NM_014723	*SNPH*	syntaphilin	5.044871	0.000121779
8862	NM_017413	*APLN*	apelin	4.968855	2.56267E-24
157739	NR_001578	*TDH*	L-threonine dehydrogenase (pseudogene)	4.847489	8.61668E-05
4153	NM_000242	*MBL2*	mannose binding lectin 2	4.577801	1.03006E-31
57214	NM_001293298,NM_001293304,NM_018689	*CEMIP*	cell migration inducing hyaluronan binding protein	4.437332	1.28742E-10
222611	NM_153839	*ADGRF2*	adhesion G protein-coupled receptor F2	4.409306	9.89236E-81
6376	NM_001304392,NM_002996	*CX3CL1*	C-X3-C motif chemokine ligand 1	4.301910	1.69932E-08
7039	NM_001099691,NM_001308158,NM_001308159,NM_003236	*TGFA*	transforming growth factor alpha	4.242098	2.21449E-09
1767	NM_001369	*DNAH5*	dynein axonemal heavy chain 5	4.109003	0.002552756
5328	NM_001145031,NM_001319191,NM_002658	*PLAU*	plasminogen activator, urokinase	4.099523	4.56426E-20
30817	NM_001271052,NM_013447	*ADGRE2*	adhesion G protein-coupled receptor E2	4.072615	0.000224211
56892	NM_020130	*C8orf4*	chromosome 8 open reading frame 4	3.930674	2.83578E-11
5055	NM_001143818,NM_002575	*SERPINB2*	serpin family B member 2	3.864791	0.000200576
4884	NM_002522	*NPTX1*	neuronal pentraxin 1	3.717525	5.16782E-12
2920	NM_002089	*CXCL2*	C-X-C motif chemokine ligand 2	3.691396	4.72124E-31
3754	NM_002236	*KCNF1*	potassium voltage-gated channel modifier subfamily F member 1	3.657794	4.51532E-07
203190	NM_139278	*LGI3*	leucine rich repeat LGI family member 3	3.603554	0.02580597
64386	NM_022468	*MMP25*	matrix metallopeptidase 25	3.539046	0.001484963

107303344	NM_001320727,NR_135324	*SETDB2-PHF11*	SETDB2-PHF11 readthrough	3.504963	0.001045233
10170	NM_001142270,NM_001142271,NM_001289763,NM_199204	*DHRS9*	dehydrogenase/reductase 9	3.493099	6.31444E-09
56961	NM_020209	*SHD*	Src homology 2 domain containing transforming protein D	3.426606	0.001619291
135112	NM_001122842,NM_001199619,NM_001199620,NM_001199621,NM_001199622,NM_181782	*NCOA7*	nuclear receptor coactivator 7	3.419905	1.9313E-102
125488	NM_001135993,NM_001243425,NM_001292030,NM_153211	*TTC39C*	tetratricopeptide repeat domain 39C	3.345688	6.39928E-21
4837	NM_006169	*NNMT*	nicotinamide N-methyltransferase	3.295695	0.016270772
3708	NM_001099952,NM_001168272,NM_002222	*ITPR1*	inositol 1,4,5-trisphosphate receptor type 1	3.293136	7.47749E-19
9308	NM_001040280,NM_001251901,NM_004233	*CD83*	CD83 molecule	3.214823	5.78202E-11
135644	NM_001286633,NM_138700	*TRIM40*	tripartite motif containing 40	3.204277	0.000157358
3604	NM_001561	*TNFRSF9*	TNF receptor superfamily member 9	3.166664	2.02967E-06
8792	NM_001270949,NM_001270950,NM_001270951,NM_001278268,NM_003839	*TNFRSF11A*	TNF receptor superfamily member 11a	3.143917	2.60427E-05
57491	NM_001242412,NM_020731	*AHRR*	aryl-hydrocarbon receptor repressor	3.104681	7.82136E-11
115811	NM_001330452,NM_138451	*IQCD*	IQ motif containing D	3.090306	3.50443E-05
65989	NM_001286655,NM_001286656,NM_023932,NM_206539	*DLK2*	delta like non-canonical Notch ligand 2	3.078482	0.000497401

1958	NM_001964	*EGR1*	early growth response 1	3.034575	1.05852E-10
1846	NM_001394,NM_057158	*DUSP4*	dual specificity phosphatase 4	3.030126	2.81015E-64
26166	NM_001286692,NM_001286693,NM_015668	*RGS22*	regulator of G protein signaling 22	3.019214	0.003648883
1435	NM_000757,NM_172210,NM_172211,NM_172212	*CSF1*	colony stimulating factor 1	3.013399	2.77182E-17
135398	NM_001145652,NR_146853,NR_146854,NR_146855,NR_146856,NR_146857,NR_146858,NR_146859,NR_146860,NR_146861,NR_146862,NR_146863,NR_146864	*C6orf141*	chromosome 6 open reading frame 141	2.982983	7.17801E-15
3484	NM_000596	*IGFBP1*	insulin like growth factor binding protein 1	2.973367	7.5434E-06
5629	NM_001270616,NM_002763	*PROX1*	prospero homeobox 1	2.958410	3.20709E-82
6654	NM_005633	*SOS1*	SOS Ras/Rac guanine nucleotide exchange factor 1	2.935131	9.661E-79
220	NM_000693,NM_001293815	*ALDH1A3*	aldehyde dehydrogenase 1 family member A3	2.923598	2.85371E-16
100533970	NR_037928	*P2RX5-TAX1BP3*	P2RX5-TAX1BP3 readthrough (NMD candidate)	2.892283	0.023672889
255239	NM_178510	*ANKK1*	ankyrin repeat and kinase domain containing 1	2.890317	0.000895666
9953	NM_006041,NR_130138	*HS3ST3B1*	heparan sulfate-glucosamine 3-sulfotransferase 3B1	2.881504	0.000615251
8676	NM_003764	*STX11*	syntaxin 11	2.871522	0.000246709
12	NM_001085	*SERPINA3*	serpin family A member 3	2.867497	3.64128E-10

92610	NM_052864	*TIFA*	TRAF interacting protein with forkhead associated domain	2.851643	2.73503E-33
57822	NM_001195010,NM_021180,NM_198173,NM_198174	*GRHL3*	grainyhead like transcription factor 3	2.848036	1.20894E-16
161742	NM_152594	*SPRED1*	sprouty related EVH1 domain containing 1	2.841740	4.44435E-39
100132234	NR_135254	*LINC00543*	long intergenic non-protein coding RNA 543	2.837947	1.92005E-46
5054	NM_000602	*SERPINE1*	serpin family E member 1	2.829997	1.78814E-07
4583	NM_002457	*MUC2*	mucin 2, oligomeric mucus/gel-forming	2.828962	0.034386661
130367	NM_001320833,NM_001320834,NM_152386	*SGPP2*	sphingosine-1-phosphate phosphatase 2	2.822704	1.00891E-06
84239	NM_032279	*ATP13A4*	ATPase 13A4	2.789804	0.00012624
23764	NM_001161572,NM_001161573,NM_001161574,NM_012323	*MAFF*	MAF bZIP transcription factor F	2.776537	3.54344E-44
54361	NM_030761	*WNT4*	Wnt family member 4	2.773242	8.64033E-08
84807	NM_001321831,NM_139239	*NFKBID*	NFKB inhibitor delta	2.768587	8.43041E-13
55691	NM_001318336,NM_001318337,NM_001318338,NM_018027,NR_134578	*FRMD4A*	FERM domain containing 4A	2.743152	1.4668E-59
6495	NM_005982	*SIX1*	SIX homeobox 1	2.728002	0.020547847
440603	NM_001010922	*BCL2L15*	BCL2 like 15	2.726637	0.018860821
189	NM_000030	*AGXT*	alanine-glyoxylate aminotransferase	2.713616	0.012098756
2571	NM_000817,NM_013445	*GAD1*	glutamate decarboxylase 1	2.707207	2.80963E-34

145837	NR_026979	*DRAIC*	downregulated RNA in cancer, inhibitor of cell invasion and migration	2.696567	3.69627E-06
9982	NM_005130	*FGFBP1*	fibroblast growth factor binding protein 1	2.689650	4.47873E-27
654321	NR_002921	*SNORA75*	small nucleolar RNA, H/ACA box 75	2.654799	0.040579853
4208	NM_001131005,NM_001193347,NM_001193348,NM_001193349,NM_001193350,NM_001308002,NM_002397	*MEF2C*	myocyte enhancer factor 2C	2.649429	3.83792E-06
54979	NM_017878	*HRASLS2*	HRAS like suppressor 2	2.647330	2.16615E-08
201501	NM_001039360,NM_001318841	*ZBTB7C*	zinc finger and BTB domain containing 7C	2.616356	0.017686559
647033	NR_003284	*PA2G4P4*	proliferation-associated 2G4 pseudogene 4	2.614045	1.8801E-07
89884	NM_033343	*LHX4*	LIM homeobox 4	2.611339	0.023877991
83648	NM_053279	*FAM167A*	family with sequence similarity 167 member A	2.602534	0.047130603
3553	NM_000576	*IL1B*	interleukin 1 beta	2.584909	0.001061401
29931	NR_024065	*LINC00312*	long intergenic non-protein coding RNA 312	2.571528	0.039453593
9429	NM_001257386,NM_001348985,NM_001348986,NM_001348987,NM_001348988,NM_001348989,NM_004827	*ABCG2*	ATP binding cassette subfamily G member 2 (Junior blood group)	2.558833	1.09774E-41
319	NM_001638	*APOF*	apolipoprotein F	2.526152	0.015938001
5724	NM_000952,NM_001164721,NM_001164722,NM_001164723	*PTAFR*	platelet activating factor receptor	2.498119	0.000197894

6288	NM_000331,NM_001178006,NM_199161	*SAA1*	serum amyloid A1	2.486918	0.000416395
7475	NM_006522	*WNT6*	Wnt family member 6	2.473360	0.021375431
440823	NR_003491,NR_033319,NR_033320,NR_033321	*MIAT*	myocardial infarction associated transcript (non-protein coding)	2.465545	0.024486896
7128	NM_001270507,NM_001270508,NM_006290	*TNFAIP3*	TNF alpha induced protein 3	2.451854	1.51439E-79
54873	NM_017734	*PALMD*	palmdelphin	2.449319	0.000442443
7474	NM_001256105,NM_003392	*WNT5A*	Wnt family member 5A	2.448495	0.000242313
8870	NM_003897	*IER3*	immediate early response 3	2.434411	1.98443E-32
389384	NM_001010903	*C6orf222*	chromosome 6 open reading frame 222	2.428782	8.10536E-40
80149	NM_001323550,NM_001323551,NM_025079	*ZC3H12A*	zinc finger CCCH-type containing 12A	2.425756	5.61079E-29
25780	NM_001139488,NM_001349975,NM_001349976,NM_001349977,NM_001349978,NM_001349979,NM_001349980,NM_001349981,NM_015376,NM_170672	*RASGRP3*	RAS guanyl releasing protein 3	2.423040	4.12174E-15
8140	NM_003486	*SLC7A5*	solute carrier family 7 member 5	2.422347	2.4074E-113
23308	NM_001283050,NM_001283051,NM_001283052,NM_015259	*ICOSLG*	inducible T-cell costimulator ligand	2.416542	3.47898E-45
80117	NM_025047	*ARL14*	ADP ribosylation factor like GTPase 14	2.414233	2.39001E-20
6752	NM_001050	*SSTR2*	somatostatin receptor 2	2.401737	0.001139554

3656	NM_001570	*IRAK2*	interleukin 1 receptor associated kinase 2	2.394587	7.72303E-43
2353	NM_005252	*FOS*	Fos proto-oncogene, AP-1 transcription factor subunit	2.394276	1.09554E-28
54658	NM_000463	*UGT1A1*	UDP glucuronosyltransferase family 1 member A1	2.386919	2.54865E-13
54757	NM_001243746,NM_017565,NR_027751	*FAM20A*	FAM20A, golgi associated secretory pathway pseudokinase	2.386539	4.80758E-22
8843	NM_006018	*HCAR3*	hydroxycarboxylic acid receptor 3	2.379895	0.014718691
27289	NM_014470	*RND1*	Rho family GTPase 1	2.376839	1.41012E-16
23120	NM_025153	*ATP10B*	ATPase phospholipid transporting 10B (putative)	2.374564	8.60165E-25
574029	NR_002834	*DUSP5P1*	dual specificity phosphatase 5 pseudogene 1	2.361572	3.94878E-05
1009	NM_001308392,NM_001330576,NM_001797	*CDH11*	cadherin 11	2.347858	2.27436E-06
9283	NM_004767	*GPR37L1*	G protein-coupled receptor 37 like 1	2.343529	6.30748E-07
728689	NM_001099661,NM_001099661_dup1,NM_001317856,NM_001317856_dup1,NM_001317857,NM_001317857_dup1	*EIF3CL*	eukaryotic translation initiation factor 3 subunit C like	2.323830	0.037963382
7477	NM_058238	*WNT7B*	Wnt family member 7B	2.318639	0.000915839
57624	NM_020864	*NYAP2*	neuronal tyrosine-phosphorylated phosphoinositide-3-kinase adaptor 2	2.310490	0.018842612
100499467	NR_036488,NR_137280,NR_137281	*LINC00673*	long intergenic non-protein coding RNA 673	2.307246	2.53429E-37

5581	NM_005400	*PRKCE*	protein kinase C epsilon	2.295835	2.80756E-10
654463	NM_001039112	*FER1L6*	fer-1 like family member 6	2.286943	2.09537E-09
1848	NM_001946,NM_022652	*DUSP6*	dual specificity phosphatase 6	2.284662	9.61239E-61
54575	NM_019075	*UGT1A10*	UDP glucuronosyltransferase family 1 member A10	2.282239	0.022268013
30811	NM_014586	*HUNK*	hormonally up-regulated Neu-associated kinase	2.281565	1.28083E-13
374	NM_001657,NM_001657_dup1	*AREG*	amphiregulin	2.265958	1.86558E-45
8193	NM_001135155,NM_001135156,NM_001289978,NM_004647	*DPF1*	double PHD fingers 1	2.249271	0.001403238
93432	NM_001293626	*MGAM2*	maltase-glucoamylase 2 (putative)	2.244787	4.33373E-08
56104	NM_018922,NM_032095	*PCDHGB1*	protocadherin gamma subfamily B, 1	2.239358	0.014739616
220108	NM_001242312,NM_001330522,NM_145019	*FAM124A*	family with sequence similarity 124 member A	2.237977	1.58417E-05
4792	NM_020529	*NFKBIA*	NFKB inhibitor alpha	2.237940	4.34581E-58
64150	NR_002770	*DIO3OS*	DIO3 opposite strand/antisense RNA (head to head)	2.232847	1.46553E-06
10537	NM_006398	*UBD*	ubiquitin D	2.232811	1.39373E-18
7483	NM_003395	*WNT9A*	Wnt family member 9A	2.231213	0.017078306
29995	NM_001278233,NM_001278234,NM_001278235,NM_014583	*LMCD1*	LIM and cysteine rich domains 1	2.227904	2.11388E-19
23025	NM_001080421	*UNC13A*	unc-13 homolog A	2.224093	0.016752745
4843	NM_000625	*NOS2*	nitric oxide synthase 2	2.210390	3.04726E-31

6091	NM_001145845,NM_002941,NM_133631	*ROBO1*	roundabout guidance receptor 1	2.208295	0.044785203
7555	NM_001127192,NM_001127193,NM_001127194,NM_001127195,NM_001127196,NM_003418	*CNBP*	CCHC-type zinc finger nucleic acid binding protein	2.207657	0.019932957
405753	NM_207581	*DUOXA2*	dual oxidase maturation factor 2	2.204369	4.15127E-06
29947	NM_013369,NM_175867	*DNMT3L*	DNA methyltransferase 3 like	2.203337	0.011777928
131601	NM_001136053,NM_001142646,NR_073377	*TPRA1*	transmembrane protein adipocyte associated 1	2.196975	1.63682E-46
8061	NM_001300844,NM_001300855,NM_001300856,NM_001300857,NM_005438,NR_125339	*FOSL1*	FOS like 1, AP-1 transcription factor subunit	2.189346	1.7784E-11
91768	NM_001100619,NM_001256438,NM_138375,NR_023359	*CABLES1*	Cdk5 and Abl enzyme substrate 1	2.188216	1.19381E-51
552900	NM_001031827,NM_001031827_dup1,NM_001320579,NM_001320579_dup1,NR_135304,NR_135304_dup1	*BOLA2*	bolA family member 2	2.177954	2.81382E-05
54600	NM_021027	*UGT1A9*	UDP glucuronosyltransferase family 1 member A9	2.177157	0.013370381
245972	NM_152565	*ATP6V0D2*	ATPase H+ transporting V0 subunit d2	2.164111	5.02506E-10
23491	NM_001185176,NM_001185177,NM_024922	*CES3*	carboxylesterase 3	2.157791	0.020692734
6289	NM_001127380,NM_030754	*SAA2*	serum amyloid A2	2.157295	1.30982E-05
6098	NM_002944	*ROS1*	ROS proto-oncogene 1, receptor tyrosine kinase	2.157269	0.029377843

2254	NM_002010	*FGF9*	fibroblast growth factor 9	2.151630	0.000163477
2113	NM_001143820,NM_001162422,NM_001330451,NM_005238	*ETS1*	ETS proto-oncogene 1, transcription factor	2.147555	1.1921E-11
137994	NM_001199659,NM_001199660,NM_001286787,NM_001286819,NM_001286821,NM_001330515,NM_144652	*LETM2*	leucine zipper and EF-hand containing transmembrane protein 2	2.142534	8.37963E-09
768239	NM_001085382	*PSAPL1*	prosaposin-like 1 (gene/pseudogene)	2.136861	2.11058E-31
6776	NM_001288718,NM_001288719,NM_001288720,NM_003152	*STAT5A*	signal transducer and activator of transcription 5A	2.133961	1.98405E-12
23034	NM_001161576,NM_001161577,NM_015589	*SAMD4A*	sterile alpha motif domain containing 4A	2.133441	0.007907484
26154	NM_015657,NM_173076,NR_103740	*ABCA12*	ATP binding cassette subfamily A member 12	2.125461	0.016040985
80153	NM_001142443,NM_001142444,NM_001351378,NM_001351379,NM_025083	*EDC3*	enhancer of mRNA decapping 3	2.121158	4.42759E-70
3038	NM_001199280,NM_005329,NM_138612	*HAS3*	hyaluronan synthase 3	2.106333	5.43929E-13
89766	NM_001004416,NM_001199527,NM_001199528,NM_173568	*UMODL1*	uromodulin like 1	2.092678	0.004244456
340152	NM_207360	*ZC3H12D*	zinc finger CCCH-type containing 12D	2.084429	4.59396E-09
6493	NM_005069,NM_009586	*SIM2*	single-minded family bHLH transcription factor 2	2.083500	5.65789E-09
10562	NM_006418	*OLFM4*	olfactomedin 4	2.080013	0.03806197

8707	NM_003783	*B3GALT2*	beta-1,3-galactosyltransferase 2	2.079748	4.78676E-07
4155	NM_001025081,NM_001025090,NM_001025092,NM_001025100,NM_001025101,NM_002385	*MBP*	myelin basic protein	2.077663	7.07534E-40
85027	NM_032947	*SMIM3*	small integral membrane protein 3	2.075580	4.91221E-31
1827	NM_001285389,NM_001285391,NM_001285392,NM_001285393,NM_001331016,NM_004414,NM_203417,NM_203418	*RCAN1*	regulator of calcineurin 1	2.057258	1.32803E-43
9901	NM_001033117,NM_014850	*SRGAP3*	SLIT-ROBO Rho GTPase activating protein 3	2.050598	4.38625E-11
7043	NM_001329938,NM_001329939,NM_003239	*TGFB3*	transforming growth factor beta 3	2.048539	5.73353E-08
2069	NM_001432	*EREG*	epiregulin	2.026960	9.06946E-46
83697	NM_001258426,NM_001258427,NM_001258428,NM_031467	*SLC4A9*	solute carrier family 4 member 9	2.022015	0.031686334
22822	NM_007350	*PHLDA1*	pleckstrin homology like domain family A member 1	2.021287	1.56272E-29
643596	NM_001190228	*RNF224*	ring finger protein 224	2.018550	0.013656647
5971	NM_006509	*RELB*	RELB proto-oncogene, NF-kB subunit	2.017163	1.90018E-21
4973	NM_001172632,NM_001172633,NM_002543	*OLR1*	oxidized low density lipoprotein receptor 1	2.015680	7.86678E-23
3659	NM_002198	*IRF1*	interferon regulatory factor 1	2.006544	1.09E-27
56999	NM_001318781,NM_182920	*ADAMTS9*	ADAM metallopeptidase with thrombospondin	2.006445	1.13335E-16

			type 1 motif 9		
2637	NM_001301687,NM_001485	*GBX2*	gastrulation brain homeobox 2	2.006334	0.000168966
2302	NM_001454	*FOXJ1*	forkhead box J1	-2.021778	0.000452699
11248	NM_007225	*NXPH3*	neurexophilin 3	-2.037506	0.000345599
7277	NM_001278552,NM_006000	*TUBA4A*	tubulin alpha 4a	-2.046417	0.029912364
57576	NM_001122819,NM_001287212,NM_020816	*KIF17*	kinesin family member 17	-2.049606	0.0146898
100335030	NR_033267	*LOC100335030*	FGFR1 oncogene partner 2 pseudogene	-2.065713	0.008546575
100506084	NM_001039083,NM_001103154,NM_001330240,NM_001330240_dup1,NM_001330240_dup2	*ARL17B*	ADP ribosylation factor like GTPase 17B	-2.139146	0.000723866
10451	NM_001079874,NM_006113	*VAV3*	vav guanine nucleotide exchange factor 3	-2.193649	1.05066E-14
11037	NM_001198595,NM_006873	*STON1*	stonin 1	-2.199848	0.00237274
105378744	NR_147163	*LINC01767*	long intergenic non-protein coding RNA 1767	-2.217439	0.036423777
2826	NM_016602	*CCR10*	C-C motif chemokine receptor 10	-2.264296	0.002585618
100302285	NR_036052_chr12,NR_036052_chr2,NR_036052_chr5,NR_036052_dup1	*MIR1244-1*	microRNA 1244-1	-2.267974	0.005032682
388121	NM_001311175,NM_207381	*TNFAIP8L3*	TNF alpha induced protein 8 like 3	-2.277357	0.024593843
25790	NM_012337	*CFAP45*	cilia and flagella associated protein 45	-2.316005	0.032593285
57529	NM_020769	*RTL9*	retrotransposon Gag like 9	-2.337786	0.042098959

81031	NM_030777	*SLC2A10*	solute carrier family 2 member 10	-2.406325	0.011579043
7140	NM_001042780,NM_001042781,NM_001042782,NM_001297646,NM_006757	*TNNT3*	troponin T3, fast skeletal type	-2.426537	0.04018045
100529241	NM_001202485	*HSPE1-MOB4*	HSPE1-MOB4 readthrough	-2.497132	0.049725656
80739	NM_025260,NM_138272,NM_138273,NM_138274,NM_138275,NM_138277	*MPIG6B*	megakaryocyte and platelet inhibitory receptor G6b	-2.534046	0.043816712
56154	NM_001350162,NR_146525	*TEX15*	testis expressed 15, meiosis and synapsis associated	-2.610966	0.035846536
101929069	NR_146489	*LINC01633*	long intergenic non-protein coding RNA 1633	-2.622860	0.03609831
2702	NM_005266,NM_181703	*GJA5*	gap junction protein alpha 5	-2.640448	0.03257668
148418	NM_001010971,NM_001134663,NM_001134664	*SAMD13*	sterile alpha motif domain containing 13	-2.641731	0.013972694
100532724	NR_037804	*NPHP3-ACAD11*	NPHP3-ACAD11 readthrough (NMD candidate)	-2.644890	0.00635894
154091	NM_145176	*SLC2A12*	solute carrier family 2 member 12	-2.658703	0.044603932
339988	NR_147207	*LINC002481*	long intergenic non-protein coding RNA 2481	-2.686400	0.03501052
5321	NM_001311193,NM_024420	*PLA2G4A*	phospholipase A2 group IVA	-2.739050	0.017679096
101927528	NR_104644,NR_104645	*LINC01204*	long intergenic non-protein coding RNA 1204	-2.824134	0.025398221
552891	NM_004125	*DNAJC25-GNG10*	DNAJC25-GNG10 readthrough	-2.923793	2.76185E-05
105377623	NR_136203	*LOC105377623*	uncharacterized LOC105377623	-2.961757	0.042343419
387856	NM_001013635	*CCDC184*	coiled-coil domain containing 184	-3.632869	0.006598134

100526767	NM_001198954	*RNF103-CHMP3*	RNF103-CHMP3 readthrough	-3.662446	0.00207372
27185	NM_001012957,NM_001012958,NM_001012959,NM_001164537,NM_001164538,NM_001164539,NM_001164540,NM_001164541,NM_001164542,NM_001164544,NM_001164545,NM_001164546,NM_001164547,NM_001164548,NM_001164549,NM_001164550,NM_001164551,NM_001164552,NM_001164553,NM_001164554,NM_001164555,NM_001164556,NM_018662	*DISC1*	disrupted in schizophrenia 1	-3.835430	0.03197595
414060	NM_001001418,NM_001001418_dup1	*TBC1D3C*	TBC1 domain family member 3C	-3.881845	0.006547149
386593	NR_027928	*CHKB-CPT1B*	CHKB-CPT1B readthrough (NMD candidate)	-3.927349	0.046335632
100526832	NM_001199290,NR_144936	*PHOSPHO2-KLHL23*	PHOSPHO2-KLHL23 readthrough	-4.108550	0.001683051
100533195	NR_037915	*FAM24B-CUZD1*	FAM24B-CUZD1 readthrough	-4.524952	0.024170776
26134	NR_104269	*RALGAPA1P1*	Ral GTPase activating protein catalytic alpha subunit 1 pseudogene 1	-4.679238	0.020276568
100616394	NR_039646_chr2,NR_039646_chr3	*MIR4444-1*	microRNA 4444-1	-8.983079	0.002262391
102465252	NR_106761	*MIR6506*	microRNA 6506	-11.739658	0.008430155
170688	NR_104005	*NUDT4P2*	nudix hydrolase 4 pseudogene 2	-12.648102	0.007944917

Table S5. Gene ontology (GO) enrichment analysis of differentially expressed genes between Caco-2 cells stimulated with wild-type spore and control.

GO ID	GO description	*p*-value	Corrected *p*-value[a]	Cluster frequency[b]	Total frequency[c]	Genes
**Biological process**						
6935	chemotaxis	4.03E-08	2.16E-05	8/54 14.8%	169/17791 0.9%	*ROBO3 CXCL10 CXCL8 CCL20 SLIT1 SAA1 SAA2 CXCL1*
42330	taxis	4.03E-08	2.16E-05	8/54 14.8%	169/17791 0.9%	*ROBO3 CXCL10 CXCL8 CCL20 SLIT1 SAA1 SAA2 CXCL1*
40011	locomotion	6.99E-07	2.49E-04	10/54 18.5%	440/17791 2.4%	*ROBO3 CXCL10 CXCL8 DNAH2 CCL20 SLIT1 SAA1 SIX1 SAA2 CXCL1*
7626	locomotory behavior	1.57E-06	4.19E-04	8/54 14.8%	273/17791 1.5%	*ROBO3 CXCL10 CXCL8 CCL20 SLIT1 SAA1 SAA2 CXCL1*
6954	inflammatory response	4.64E-06	8.27E-04	8/54 14.8%	316/17791 1.7%	*CXCL10 CXCL8 CCL20 PLA2G4B STAB1 SAA1 SAA2 CXCL1*
7610	behavior	1.00E-05	1.53E-03	9/54 16.6%	466/17791 2.6%	*ROBO3 CXCL10 CXCL8 TAL2 CCL20 SLIT1 SAA1 SAA2 CXCL1*
30593	neutrophil chemotaxis	2.47E-05	2.65E-03	3/54 5.5%	19/17791 0.1%	*CXCL8 SAA1 SAA2*
9611	response to wounding	3.31E-05	3.22E-03	9/54 16.6%	542/17791 3.0%	*CXCL10 KLF6 CXCL8 CCL20 PLA2G4B STAB1 SAA1 SAA2 CXCL1*
9605	response to external stimulus	3.77E-05	3.36E-03	9/54 16.6%	551/17791 3.0%	*ROBO3 CXCL10 CXCL8 CCL20 SLIT1 SAA1 SAA2 CXCL1 SSTR2*

6952	defense response	9.47E-05	7.80E-03	9/54<br>16.6%	621/17791<br>3.4%	*CXCL10 CXCL8 CD83 CCL20 PLA2G4B STAB1 SAA1 SAA2 CXCL1*
48247	lymphocyte chemotaxis	1.88E-04	1.44E-02	2/54<br>3.7%	7/17791<br>0.0%	*SAA1 SAA2*
30595	leukocyte chemotaxis	2.99E-04	2.14E-02	3/54<br>5.5%	43/17791<br>0.2%	*CXCL8 SAA1 SAA2*
51674	localization of cell	3.71E-04	2.24E-02	6/54<br>11.1%	315/17791<br>1.7%	*CXCL8 DNAH2 SLIT1 SAA1 SIX1 SAA2*
48870	cell motility	3.71E-04	2.24E-02	6/54<br>11.1%	315/17791<br>1.7%	*CXCL8 DNAH2 SLIT1 SAA1 SIX1 SAA2*
60326	cell chemotaxis	4.15E-04	2.34E-02	3/54<br>5.5%	48/17791<br>0.2%	*CXCL8 SAA1 SAA2*
48246	macrophage chemotaxis	4.89E-04	2.54E-02	2/54<br>3.7%	11/17791<br>0.0%	*SAA1 SAA2*
2376	immune system process	4.99E-04	2.54E-02	10/54<br>18.5%	949/17791<br>5.3%	*CXCL10 KLF6 RIT1 CXCL8 CD83 CCL20 SAA1 SIX1 SAA2 CXCL1*
6928	cellular component movement	5.47E-04	2.66E-02	7/54<br>12.9%	474/17791<br>2.6%	*ROBO3 CXCL8 DNAH2 SLIT1 SAA1 SIX1 SAA2*
50716	positive regulation of interleukin-1 secretion	8.04E-04	3.74E-02	2/54<br>3.7%	14/17791<br>0.0%	*SAA1 SAA2*
50900	leukocyte migration	9.23E-04	3.96E-02	3/54<br>5.5%	63/17791<br>0.3%	*CXCL8 SAA1 SAA2*
50704	regulation of interleukin-1	9.26E-04	3.96E-02	2/54	15/17791	*SAA1 SAA2*

	secretion			3.7%	0.0%	
**Molecular Function**						
1664	G-protein-coupled receptor binding	3.41E-06	7.30E-04	6/54 11.1%	136/17791 0.7%	*CXCL10 CXCL8 CCL20 SAA1 SAA2 CXCL1*
8009	chemokine activity	1.23E-05	1.64E-03	4/54 7.4%	47/17791 0.2%	*CXCL10 CXCL8 CCL20 CXCL1*
42379	chemokine receptor binding	1.84E-05	2.19E-03	4/54 7.4%	52/17791 0.2%	*CXCL10 CXCL8 CCL20 CXCL1*
6935	chemotaxis	4.03E-08	2.16E-05	8/54 14.8%	169/17791 0.9%	*ROBO3 CXCL10 CXCL8 CCL20 SLIT1 SAA1 SAA2 CXCL1*
**Cellular component**						
5615	extracellular space	3.77E-04	2.24E-02	9/54 16.6%	747/17791 4.1%	*CXCL10 CXCL8 CCL20 SLIT1 SAA1 PRH2 SAA2 CXCL1 FBN1*

[a] *p*-value in hypergeometric test after correction

[b] The denominator represents the total number of genes with GO annotation and the numerator represents the number of genes belonging to each GO term

[c] The denominator represents the number of reference genes with GO annotation and the numerator represents the number of references genes annotated in the listed GO terms

Table S6. Gene Ontology (GO) enrichment analysis of differentially expressed genes between Caco-2 cells stimulated with CotG-p75 and control.

GO ID	GO description	*p*-value	Corrected *p*-value[a]	Cluster frequency[b]	Total frequency[c]	Genes
**Biological process**						
42221	response to chemical stimulus	3.70E-15	5.18E-12	54/202 26.7%	1466/17784 8.2%	*ROBO3 OXTR CD83 CXCL8 TAT SERPINE1 CXCL1 CXCL3 ETS1 CXCL2 CX3CL1 ROBO1 PLAU CYP1B1 OLR1 CCR6 MBL2 SLC34A3 DUSP4 IGFBP1 UGT1A1 WNT5A FOS RNASE2 SSTR2 DUSP6 ALDH1A3 IL1B AHRR TLR7 CRYAB ABCG2 ITPR1 TNFRSF11A WNT6 IRAK2 UBD SLIT1 WNT4 STAT5A EGR1 EGR2 CCL22 NOS2 CCL20 WNT7B FOSL1 NFKBIA CXCL10 CXCL11 CYP1A1 SAA1 SAA2 FSBP*
42330	taxis	5.20E-15	5.18E-12	20/202 9.9%	169/17784 0.9%	*ROBO3 CCL22 CXCL8 CCL20 CXCL1 TNFRSF11A RNASE2 CXCL3 CXCL2 CX3CL1 ROBO1 FOSL1 CXCL10 CXCL11 PLAU IL1B SAA1 SLIT1 SAA2 CCR6*
6935	chemotaxis	5.20E-15	5.18E-12	20/202 9.9%	169/17784 0.9%	*ROBO3 CCL22 CXCL8 CCL20 CXCL1 TNFRSF11A RNASE2 CXCL3 CXCL2 CX3CL1 ROBO1 FOSL1 CXCL10 CXCL11 PLAU IL1B SAA1 SLIT1 SAA2 CCR6*
6952	defense response	6.97E-13	5.21E-10	32/202 15.8%	621/17784 3.4%	*SERPINA3 CD83 CXCL8 SERPINE1 CXCL1 CXCL3 CXCL2 CX3CL1 MMP25 IRAK2 UBD TIFA OLR1 S1PR3 CCR6 ICOSLG MBL2 CCL22 NOS2 CCL20 WNT5A FOS FOSL1 CXCL10 CXCL11 IL1B BCL3 SAA1 LCN2 SAA2 TLR7 NFKBID*
9605	response to external stimulus	1.03E-12	6.14E-10	30/202 14.8%	552/17784 3.1%	*ROBO3 CXCL8 CXCL1 TNFRSF11A CXCL3 CXCL2 CX3CL1 ROBO1 WNT6 PLAU GJA10 SLIT1 CCR6*

						SLC34A3 CCL22 UGT1A1 CCL20 WNT7B WNT5A FOS RNASE2 SSTR2 FOSL1 CXCL10 CXCL11 IL1B CYP1A1 SAA1 AHRR SAA2
48518	positive regulation of biological process	1.43E-12	7.13E-10	63/202 31.1%	2210/17784 12.4%	*OXTR CD83 CXCL8 CSF1 SERPINE1 ADRA1D AREG ETS1 CX3CL1 ICAM1 ROBO1 FGF9 IKBKE LMCD1 PHLDA1 ICOSLG MBL2 VAV3 IGFBP1 MEF2C FGFBP1 PRKCE TESC WNT5A FOS PROX1 DUSP6 BTC ALDH1A3 IL1B IRF1 AHRR TLR7 SOS1 HBEGF FAM129A TGFA TNFRSF11A BARX2 RELB WNT6 IRAK2 UBD TIFA S1PR3 WNT4 STAT5A EGR1 EGR2 NOS2 WNT7B TNFRSF9 SAMD4A GRHL3 FOSL1 NFKBIA CXCL10 BCL3 CYP1A1 SAA1 SAA2 NFKBID TMEM9B*
9611	response to wounding	3.92E-12	1.67E-09	29/202 14.3%	543/17784 3.0%	*SERPINA3 CXCL8 SERPINE1 CXCL1 CXCL3 CXCL2 CX3CL1 MMP25 PLAU IRAK2 TIFA OLR1 S1PR3 MBL2 IGFBP1 SERPINB2 CCL22 CCL20 WNT5A GRHL3 FOS CXCL10 CXCL11 IL1B CYP1A1 SAA1 AHRR SAA2 NFKBID*
48522	positive regulation of cellular process	8.84E-12	3.30E-09	58/202 28.7%	2006/17784 11.2%	*OXTR CD83 CSF1 SERPINE1 ADRA1D AREG ETS1 CX3CL1 ICAM1 ROBO1 FGF9 IKBKE LMCD1 PHLDA1 ICOSLG MBL2 VAV3 IGFBP1 MEF2C FGFBP1 PRKCE TESC WNT5A FOS PROX1 DUSP6 BTC ALDH1A3 IL1B IRF1 AHRR TLR7 SOS1 HBEGF FAM129A TGFA TNFRSF11A BARX2 UBD TIFA S1PR3 WNT4 STAT5A EGR1 EGR2 WNT7B TNFRSF9 SAMD4A GRHL3 FOSL1 NFKBIA CXCL10 BCL3 CYP1A1 SAA1 SAA2 NFKBID TMEM9B*
2376	immune system process	3.68E-11	1.10E-08	37/202 18.3%	949/17784 5.3%	*DYRK3 CD83 CXCL8 CSF1 CXCL1 TNFRSF11A CXCL3 ETS1 CXCL2 CX3CL1 ICAM1 RELB IRAK2 UBD CCR6 IKBKE ICOSLG MBL2 STAT5A EGR1*

						CCL22 NOS2 TIPARP CCL20 WNT5A NFKB2 APLN NFKBIA CXCL10 CXCL11 IL1B IRF1 BCL3 SAA1 LCN2 SAA2 TLR7
7626	locomotory behavior	4.31E-11	1.17E-08	20/202 9.9%	273/17784 1.5%	*ROBO3 CCL22 CXCL8 CCL20 CXCL1 TNFRSF11A RNASE2 CXCL3 CXCL2 CX3CL1 ROBO1 FOSL1 CXCL10 CXCL11 PLAU IL1B SAA1 SLIT1 SAA2 CCR6*
48513	organ development	1.33E-10	3.05E-08	52/202 25.7%	1792/17784 10.0%	*OXTR DYRK3 CXCL8 CSF1 SERPINE1 TNFAIP2 IKZF3 CX3CL1 ROBO1 FGF9 PLAU ZC3H12A VAV3 IGFBP1 MEF2C MEF2B UGT1A1 WNT5A PROX1 RGMA ALDH1A3 MRAS IRF1 AHRR ALPK3 CRYAB HBEGF LAMA4 TNFRSF11A BARX2 RELB WNT6 UBD SLIT1 WNT4 STAT5A EGR1 EGR2 NOS2 TIPARP WNT7B GRHL3 NFKB2 APLN RCAN1 CXCL10 GJB2 KRT17 DHRS9 BCL3 CYP1A1 LHX4*
23046	signaling process	1.63E-10	3.25E-08	58/202 28.7%	2157/17784 12.1%	*OXTR CD83 CXCL8 RASEF ADRA1D CXCL1 CX3CL1 RND1 RAB43 SPRED1 FGF9 PLAU CCR6 CA8 ICOSLG DUSP4 VAV3 IGFBP1 FGFBP1 ARL14 PRKCE WNT5A HUNK FOS SSTR2 DUSP6 MRAS SYPL1 SNPH IL1B TLR7 SOS1 CRYAB HBEGF ITPR1 TGFA DAPP1 TNFRSF11A GNAI1 RASGRP3 IRAK2 TIFA SLIT1 S1PR3 NPTX1 STAT5A EGR2 CCL22 NOS2 CCL20 GAD1 NFKB2 APLN NFKBIA RCAN1 CXCL10 CXCL11 BCL3*
23060	signal transmission	1.63E-10	3.25E-08	58/202 28.7%	2157/17784 12.1%	*OXTR CD83 CXCL8 RASEF ADRA1D CXCL1 CX3CL1 RND1 RAB43 SPRED1 FGF9 PLAU CCR6 CA8 ICOSLG DUSP4 VAV3 IGFBP1 FGFBP1 ARL14 PRKCE WNT5A HUNK FOS SSTR2 DUSP6 MRAS SYPL1 SNPH IL1B TLR7 SOS1 CRYAB HBEGF ITPR1 TGFA DAPP1 TNFRSF11A GNAI1 RASGRP3 IRAK2 TIFA SLIT1 S1PR3 NPTX1 STAT5A EGR2*

						CCL22 NOS2 CCL20 GAD1 NFKB2 APLN NFKBIA RCAN1 CXCL10 CXCL11 BCL3
48731	system development	2.26E-10	4.23E-08	62/202 30.6%	2422/17784 13.6%	*ROBO3 OXTR DYRK3 CXCL8 CSF1 SERPINE1 TNFAIP2 CXCL1 IKZF3 CX3CL1 ROBO1 RND1 FGF9 PLAU ZC3H12A VAV3 IGFBP1 MEF2C MEF2B UGT1A1 WNT5A FOS PROX1 RGMA SLC7A5 ALDH1A3 MRAS IRF1 CDH11 AHRR ALPK3 CRYAB HBEGF LAMA4 TNFRSF11A BARX2 RELB WNT6 GJA10 UBD SLIT1 NPTX1 WNT4 STAT5A EGR1 EGR2 NOS2 TIPARP WNT7B CABLES1 GRHL3 FOXN4 NFKB2 APLN RCAN1 CXCL10 GJB2 KRT17 DHRS9 BCL3 CYP1A1 LHX4*
51239	regulation of multicellular organismal process	2.71E-10	4.77E-08	38/202 18.8%	1068/17784 6.0%	*OXTR CD83 CSF1 LAMA4 SERPINE1 TNFRSF11A ETS1 CX3CL1 ROBO1 NPPB WNT6 FGF9 PLAU SLIT1 S1PR3 LMCD1 WNT4 STAT5A EGR1 EGR2 NOS2 TESC WNT7B WNT5A PROX1 SSTR2 BTC NFKBIA CXCL10 IL1B IRF1 BCL3 MAFF SAA1 SAA2 TLR7 NFKBID HBEGF*
32502	developmental process	3.55E-10	5.89E-08	74/202 36.6%	3234/17784 18.1%	*ROBO3 OXTR DYRK3 CXCL8 CSF1 SERPINE1 TNFAIP2 ADRA1D CXCL1 IKZF3 CX3CL1 ROBO1 RND1 NPPB DNMT3L SPRED1 FGF9 PLAU ZC3H12A IER3 VAV3 IGFBP1 MEF2C MEF2B TPRA1 UGT1A1 WNT5A HUNK FOS PROX1 DUSP6 RGMA SLC7A5 ALDH1A3 MRAS IL1B IRF1 CDH11 AHRR ALPK3 CRYAB HBEGF LAMA4 ITPR1 TNFRSF11A BARX2 RELB WNT6 GJA10 UBD SLIT1 S1PR3 NPTX1 WNT4 STAT5A EGR1 EGR2 NOS2 TIPARP WNT7B CABLES1 GRHL3 FOXN4 NFKB2 APLN RCAN1 CXCL10 GJB2 KRT17 DHRS9 BCL3 MAFF CYP1A1 LHX4*
7275	multicellular organismal	3.84E-10	6.04E-08	70/202	2971/17784	*ROBO3 OXTR DYRK3 CXCL8 CSF1 SERPINE1*

	development			34.6%	16.7%	*TNFAIP2 ADRA1D CXCL1 IKZF3 CX3CL1 ROBO1 RND1 NPPB DNMT3L SPRED1 FGF9 PLAU ZC3H12A VAV3 IGFBP1 MEF2C MEF2B UGT1A1 WNT5A HUNK FOS PROX1 RGMA SLC7A5 ALDH1A3 MRAS IL1B IRF1 CDH11 AHRR ALPK3 CRYAB HBEGF LAMA4 ITPR1 TNFRSF11A BARX2 RELB WNT6 GJA10 UBD SLIT1 NPTX1 WNT4 STAT5A EGR1 EGR2 NOS2 TIPARP WNT7B CABLES1 GRHL3 FOXN4 NFKB2 APLN RCAN1 CXCL10 GJB2 KRT17 DHRS9 BCL3 MAFF CYP1A1 LHX4*
48856	anatomical structure development	4.62E-10	6.90E-08	65/202<br>32.1%	2656/17784<br>14.9%	*ROBO3 OXTR DYRK3 CXCL8 CSF1 SERPINE1 TNFAIP2 CXCL1 IKZF3 CX3CL1 ROBO1 RND1 NPPB FGF9 PLAU ZC3H12A IER3 VAV3 IGFBP1 MEF2C MEF2B UGT1A1 WNT5A FOS PROX1 RGMA SLC7A5 ALDH1A3 MRAS IRF1 CDH11 AHRR ALPK3 CRYAB HBEGF LAMA4 TNFRSF11A BARX2 RELB WNT6 GJA10 UBD SLIT1 S1PR3 NPTX1 WNT4 STAT5A EGR1 EGR2 NOS2 TIPARP WNT7B CABLES1 GRHL3 FOXN4 NFKB2 APLN RCAN1 CXCL10 GJB2 KRT17 DHRS9 BCL3 CYP1A1 LHX4*
65007	biological regulation	5.60E-10	7.89E-08	122/202<br>60.3%	6941/17784<br>39.0%	*OXTR CD83 CSF1 SERPINE1 RASEF TBC1D3F IKZF3 ETS1 TBC1D3G ICAM1 NPPB RAB43 FGF9 PLAU CYP1B1 ZNF169 PHLDA1 ICOSLG IER3 SLC34A3 IGFBP1 MEF2C MEF2B ARL14 PRKCE WNT5A BTC ITPR1 TGFA DAPP1 TNFRSF11A RASGRP3 WNT6 IRAK2 UBD PACSIN1 WNT4 STAT5A EGR1 EGR2 TNFRSF9 FOXN4 NFKB2 FOSL1 ABTB2 NFKBIA RCAN1 CXCL10 CXCL11 BCL3 MAFF CYP1A1 LCN2 LHX4 NFKBID TMEM9B SERPINA3 DYRK3 CXCL8 TNFAIP3*

						ADRA1D CXCL1 AREG CX3CL1 ROBO1 RND1 DNMT3L SPRED1 CCR6 CA8 IKBKE LMCD1 MBL2 DUSP4 VAV3 FGFBP1 SERPINB2 UGT1A1 TESC HUNK FOS PROX1 SSTR2 DUSP6 RGMA ALDH1A3 MRAS SNPH IL1B IRF1 AHRR TLR7 NCOA7 SOS1 CRYAB HBEGF BIRC3 LAMA4 RGS16 FAM129A BARX2 GNAI1 RELB PALMD TIFA SLIT1 S1PR3 CCL22 NOS2 CCL20 GAD1 WNT7B LGI3 SAMD4A CABLES1 GRHL3 APLN DHRS9 FABP6 SAA1 SAA2 FSBP
6954	inflammatory response	5.80E-10	7.89E-08	20/202<br>9.9%	316/17784<br>1.7%	*SERPINA3 CCL22 CXCL8 CCL20 CXCL1 FOS CXCL3 CXCL2 CXCL10 MMP25 CXCL11 IRAK2 IL1B TIFA SAA1 OLR1 SAA2 S1PR3 NFKBID MBL2*
7610	behavior	6.80E-10	8.84E-08	24/202<br>11.8%	466/17784<br>2.6%	*EGR1 ROBO3 OXTR EGR2 CCL22 CXCL8 CCL20 CXCL1 FOS TNFRSF11A RNASE2 CXCL3 CXCL2 CX3CL1 ROBO1 FOSL1 CXCL10 CXCL11 PLAU IL1B SAA1 SLIT1 SAA2 CCR6*
7165	signal transduction	7.15E-10	8.91E-08	52/202<br>25.7%	1878/17784<br>10.5%	*OXTR CD83 CXCL8 RASEF ADRA1D CXCL1 CX3CL1 RND1 RAB43 SPRED1 FGF9 PLAU CCR6 CA8 ICOSLG DUSP4 VAV3 IGFBP1 FGFBP1 ARL14 PRKCE WNT5A HUNK FOS SSTR2 DUSP6 MRAS IL1B TLR7 SOS1 CRYAB HBEGF ITPR1 TGFA DAPP1 TNFRSF11A GNAI1 RASGRP3 IRAK2 TIFA S1PR3 STAT5A CCL22 NOS2 CCL20 NFKB2 APLN NFKBIA RCAN1 CXCL10 CXCL11 BCL3*
40011	locomotion	1.19E-09	1.42E-07	23/202<br>11.3%	440/17784<br>2.4%	*VAV3 ROBO3 CCL22 CXCL8 CCL20 CXCL1 TNFRSF11A RNASE2 CXCL3 ETS1 CXCL2 CX3CL1 ICAM1 ROBO1 FOSL1 CXCL10 CXCL11 PLAU IL1B SAA1 SLIT1 SAA2 CCR6*
30155	regulation of cell adhesion	1.60E-09	1.84E-07	14/202	149/17784	*STAT5A VAV3 CXCL8 CSF1 TESC LAMA4*

				6.9%	0.8%	*SERPINE1 CX3CL1 ICAM1 RND1 PLAU IL1B SAA1 SAA2*
50789	regulation of biological process	1.86E-09	2.06E-07	116/202 57.4%	6552/17784 36.8%	*OXTR CD83 CSF1 SERPINE1 RASEF TBC1D3F IKZF3 ETS1 TBC1D3G ICAM1 NPPB RAB43 FGF9 PLAU ZNF169 PHLDA1 ICOSLG IER3 IGFBP1 MEF2C MEF2B ARL14 PRKCE WNT5A BTC ITPR1 TGFA DAPP1 TNFRSF11A RASGRP3 WNT6 IRAK2 UBD PACSIN1 WNT4 STAT5A EGR1 EGR2 TNFRSF9 FOXN4 NFKB2 FOSL1 ABTB2 NFKBIA RCAN1 CXCL10 CXCL11 BCL3 MAFF CYP1A1 LCN2 LHX4 NFKBID TMEM9B SERPINA3 CXCL8 TNFAIP3 ADRA1D CXCL1 AREG CX3CL1 ROBO1 RND1 DNMT3L SPRED1 CCR6 CA8 IKBKE LMCD1 MBL2 DUSP4 VAV3 FGFBP1 SERPINB2 UGT1A1 TESC HUNK FOS PROX1 SSTR2 DUSP6 RGMA ALDH1A3 MRAS IL1B IRF1 AHRR TLR7 NCOA7 SOS1 CRYAB HBEGF BIRC3 LAMA4 RGS16 FAM129A BARX2 GNAI1 RELB PALMD TIFA SLIT1 S1PR3 CCL22 NOS2 CCL20 WNT7B LGI3 SAMD4A CABLES1 GRHL3 APLN FABP6 SAA1 SAA2 FSBP*
50794	regulation of cellular process	4.37E-09	4.51E-07	111/202 54.9%	6222/17784 34.9%	*OXTR CD83 CSF1 SERPINE1 RASEF TBC1D3F IKZF3 ETS1 TBC1D3G ICAM1 NPPB RAB43 FGF9 PLAU ZNF169 PHLDA1 ICOSLG IER3 IGFBP1 MEF2C MEF2B ARL14 PRKCE WNT5A BTC ITPR1 TGFA DAPP1 TNFRSF11A RASGRP3 IRAK2 UBD PACSIN1 WNT4 STAT5A EGR1 EGR2 TNFRSF9 FOXN4 NFKB2 FOSL1 ABTB2 NFKBIA RCAN1 CXCL10 CXCL11 BCL3 MAFF CYP1A1 LCN2 LHX4 NFKBID TMEM9B CXCL8 TNFAIP3 ADRA1D CXCL1 AREG CX3CL1 ROBO1 RND1 SPRED1 CCR6 CA8 IKBKE LMCD1 MBL2 DUSP4 VAV3 FGFBP1 SERPINB2 TESC HUNK FOS PROX1*

						SSTR2 DUSP6 ALDH1A3 MRAS IL1B IRF1 AHRR TLR7 NCOA7 SOS1 CRYAB HBEGF BIRC3 LAMA4 RGS16 FAM129A BARX2 GNAI1 RELB PALMD TIFA SLIT1 S1PR3 CCL22 NOS2 CCL20 WNT7B LGI3 SAMD4A CABLES1 GRHL3 APLN FABP6 SAA1 SAA2 FSBP
6950	response to stress	8.45E-09	8.15E-07	48/202 23.7%	1775/17784 9.9%	*SERPINA3 OXTR CD83 CXCL8 TAT SERPINE1 FAM129A ITPR1 CXCL1 CXCL3 CXCL2 CX3CL1 MMP25 PLAU IRAK2 UBD TIFA OLR1 S1PR3 CCR6 IKBKE ICOSLG MBL2 IGFBP1 SERPINB2 CCL22 UGT1A1 NOS2 CCL20 WNT5A GRHL3 FOS DUSP6 FOSL1 CXCL10 CXCL11 IL1B BCL3 MAFF CYP1A1 SAA1 LCN2 AHRR SAA2 TLR7 FSBP CRYAB NFKBID*
6955	immune response	9.22E-09	8.61E-07	26/202 12.8%	619/17784 3.4%	*CD83 CXCL8 CXCL1 TNFRSF11A CXCL3 ETS1 CXCL2 CX3CL1 ICAM1 RELB UBD CCR6 IKBKE MBL2 CCL22 NOS2 CCL20 WNT5A NFKB2 APLN CXCL10 CXCL11 IL1B BCL3 LCN2 TLR7*
23052	signaling	9.54E-09	8.64E-07	69/202 34.1%	3129/17784 17.5%	*OXTR CD83 CXCL8 RASEF ADRA1D CXCL1 AREG CX3CL1 ROBO1 RND1 NPPB RAB43 SPRED1 FGF9 PLAU CCR6 CA8 IKBKE ICOSLG DUSP4 VAV3 IGFBP1 FGFBP1 ARL14 PRKCE WNT5A HUNK FOS SSTR2 DUSP6 MRAS SYPL1 SNPH IL1B TLR7 SOS1 CRYAB HBEGF BIRC3 NXPH3 ITPR1 TGFA DAPP1 TNFRSF11A GNAI1 RASGRP3 WNT6 IRAK2 TIFA SLIT1 S1PR3 NPTX1 WNT4 STAT5A EGR2 CCL22 NOS2 TIPARP CCL20 GAD1 WNT7B NFKB2 APLN NFKBIA RCAN1 CXCL10 GJB2 CXCL11 BCL3*
35556	intracellular signal transduction	2.04E-08	1.79E-06	30/202 14.8%	833/17784 4.6%	*OXTR CXCL8 RASEF TGFA ADRA1D GNAI1 RASGRP3 RND1 RAB43 SPRED1 IRAK2 TIFA*

						S1PR3 CA8 STAT5A DUSP4 VAV3 ARL14 NOS2 WNT5A SSTR2 DUSP6 NFKBIA RCAN1 MRAS IL1B BCL3 TLR7 SOS1 CRYAB
51240	positive regulation of multicellular organismal process	2.11E-08	1.80E-06	17/202 8.4%	279/17784 1.5%	*STAT5A OXTR CD83 CSF1 SERPINE1 WNT5A TNFRSF11A CX3CL1 BTC WNT6 FGF9 IL1B BCL3 SAA1 SAA2 TLR7 WNT4*
32501	multicellular organismal process	3.24E-08	2.69E-06	85/202 42.0%	4376/17784 24.6%	*SERPINA3 ROBO3 OXTR DYRK3 CXCL8 CSF1 SERPINE1 TNFAIP2 ADRA1D CXCL1 IKZF3 CX3CL1 ACTG2 ROBO1 RND1 NPPB DNMT3L SPRED1 FGF9 PLAU ZC3H12A CYP1B1 OLR1 VAV3 IGFBP1 MEF2C MEF2B UGT1A1 WNT5A HUNK FOS PROX1 SSTR2 RGMA SLC7A5 ALDH1A3 MRAS SYPL1 SNPH IL1B IRF1 CDH11 AHRR ALPK3 MYH4 CRYAB HBEGF BIRC3 LAMA4 RGS16 ITPR1 TNFRSF11A BARX2 RELB WNT6 GJA10 UBD SLIT1 S1PR3 NPTX1 WNT4 STAT5A EGR1 EGR2 NOS2 TIPARP GAD1 WNT7B CABLES1 GRHL3 FOXN4 NFKB2 APLN FOSL1 RCAN1 CXCL10 GJB2 KRT17 DHRS9 BCL3 MAFF CYP1A1 SAA1 SAA2 LHX4*
70887	cellular response to chemical stimulus	3.35E-08	2.71E-06	20/202 9.9%	401/17784 2.2%	*STAT5A IGFBP1 EGR1 CXCL8 UGT1A1 WNT7B SERPINE1 WNT5A FOS TNFRSF11A CXCL3 CX3CL1 WNT6 IL1B CYP1A1 SAA1 SLIT1 AHRR SAA2 WNT4*
30593	neutrophil chemotaxis	4.78E-08	3.76E-06	6/202 2.9%	19/17784 0.1%	*CXCL8 IL1B SAA1 SAA2 CXCL3 CX3CL1*
9617	response to bacterium	6.77E-08	4.94E-06	15/202 7.4%	232/17784 1.3%	*UGT1A1 NOS2 CCL20 SERPINE1 WNT5A FOS TNFRSF11A NFKBIA CXCL10 IRAK2 IL1B BCL3 CYP1A1 AHRR MBL2*

32496	response to lipopolysaccharide	9.39E-08	6.68E-06	11/202 5.4%	117/17784 0.6%	*NFKBIA CXCL10 UGT1A1 IRAK2 IL1B SERPINE1 WNT5A CYP1A1 AHRR FOS TNFRSF11A*
42981	regulation of apoptosis	1.21E-07	8.40E-06	29/202 14.3%	852/17784 4.7%	*SERPINE1 TNFAIP3 ETS1 CX3CL1 ROBO1 UBD IKBKE PHLDA1 IER3 STAT5A VAV3 MEF2C SERPINB2 PRKCE TNFRSF9 WNT5A DUSP6 BTC FOSL1 NFKBIA ALDH1A3 IL1B BCL3 LCN2 LHX4 SOS1 CRYAB NFKBID BIRC3*
43067	regulation of programmed cell death	1.47E-07	9.92E-06	29/202 14.3%	860/17784 4.8%	*SERPINE1 TNFAIP3 ETS1 CX3CL1 ROBO1 UBD IKBKE PHLDA1 IER3 STAT5A VAV3 MEF2C SERPINB2 PRKCE TNFRSF9 WNT5A DUSP6 BTC FOSL1 NFKBIA ALDH1A3 IL1B BCL3 LCN2 LHX4 SOS1 CRYAB NFKBID BIRC3*
51707	response to other organism	1.49E-07	9.92E-06	18/202 8.9%	358/17784 2.0%	*CCL22 UGT1A1 NOS2 CCL20 SERPINE1 WNT5A FOS TNFRSF11A FOSL1 NFKBIA CXCL10 IRAK2 IL1B BCL3 CYP1A1 AHRR TLR7 MBL2*
50896	response to stimulus	1.53E-07	9.92E-06	73/202 36.1%	3634/17784 20.4%	*SERPINA3 ROBO3 OXTR CD83 CXCL8 TAT SERPINE1 CXCL1 CXCL3 ETS1 CXCL2 CX3CL1 ICAM1 ROBO1 MMP25 PLAU CYP1B1 OLR1 CCR6 IKBKE ICOSLG MBL2 SLC34A3 DUSP4 IGFBP1 SERPINB2 UGT1A1 WNT5A FOS RNASE2 SSTR2 DUSP6 ALDH1A3 IL1B AHRR TLR7 MYH4 CRYAB ABCG2 FAM129A ITPR1 TNFRSF11A RELB WNT6 IRAK2 GJA10 UBD TIFA SLIT1 S1PR3 WNT4 STAT5A EGR1 EGR2 CCL22 NOS2 CCL20 WNT7B GRHL3 NFKB2 APLN FOSL1 NFKBIA CXCL10 CXCL11 BCL3 MAFF CYP1A1 SAA1 LCN2 SAA2 FSBP NFKBID*
10941	regulation of cell death	1.75E-07	1.11E-05	29/202 14.3%	867/17784 4.8%	*SERPINE1 TNFAIP3 ETS1 CX3CL1 ROBO1 UBD IKBKE PHLDA1 IER3 STAT5A VAV3 MEF2C SERPINB2 PRKCE TNFRSF9 WNT5A DUSP6 BTC*

						FOSL1 NFKBIA ALDH1A3 IL1B BCL3 LCN2 LHX4 SOS1 CRYAB NFKBID BIRC3
9966	regulation of signal transduction	2.07E-07	1.26E-05	27/202 13.3%	773/17784 4.3%	*CSF1 RGS16 SERPINE1 TGFA TBC1D3F TNFAIP3 TNFRSF11A TBC1D3G ROBO1 RASGRP3 SPRED1 FGF9 PLAU IRAK2 UBD TIFA SLIT1 IKBKE LMCD1 VAV3 WNT5A DUSP6 IL1B SOS1 NFKBID TMEM9B HBEGF*
23033	signaling pathway	2.27E-07	1.33E-05	50/202 24.7%	2100/17784 11.8%	*OXTR CXCL8 RASEF ADRA1D CXCL1 AREG CX3CL1 ROBO1 RND1 NPPB RAB43 SPRED1 FGF9 CA8 IKBKE DUSP4 VAV3 IGFBP1 ARL14 PRKCE WNT5A FOS SSTR2 DUSP6 MRAS IL1B TLR7 SOS1 CRYAB HBEGF BIRC3 NXPH3 TGFA TNFRSF11A GNAI1 RASGRP3 WNT6 IRAK2 TIFA SLIT1 S1PR3 WNT4 STAT5A NOS2 TIPARP WNT7B NFKBIA RCAN1 CXCL10 BCL3*
23051	regulation of signaling process	2.36E-07	1.33E-05	27/202 13.3%	778/17784 4.3%	*CSF1 RGS16 SERPINE1 TGFA TBC1D3F TNFAIP3 TNFRSF11A TBC1D3G ROBO1 RASGRP3 SPRED1 FGF9 PLAU IRAK2 UBD TIFA SLIT1 IKBKE LMCD1 VAV3 WNT5A DUSP6 IL1B SOS1 NFKBID TMEM9B HBEGF*
2237	response to molecule of bacterial origin	2.36E-07	1.33E-05	11/202 5.4%	128/17784 0.7%	*NFKBIA CXCL10 UGT1A1 IRAK2 IL1B SERPINE1 WNT5A CYP1A1 AHRR FOS TNFRSF11A*
10646	regulation of cell communication	2.74E-07	1.51E-05	34/202 16.8%	1154/17784 6.4%	*OXTR CSF1 RGS16 SERPINE1 TGFA TBC1D3F TNFAIP3 TNFRSF11A TBC1D3G ROBO1 RASGRP3 SPRED1 FGF9 PLAU IRAK2 UBD TIFA SLIT1 IKBKE LMCD1 WNT4 VAV3 EGR1 EGR2 FGFBP1 NOS2 WNT5A DUSP6 NFKBIA IL1B SOS1 NFKBID TMEM9B HBEGF*
32101	regulation of response to	3.07E-07	1.67E-05	13/202	192/17784	*STAT5A CXCL8 SERPINE1 WNT5A TNFRSF11A*

	external stimulus			6.4%	1.0%	*CX3CL1 ROBO1 CXCL10 PLAU IL1B SAA1 SAA2 TLR7*
42127	regulation of cell proliferation	3.79E-07	2.02E-05	28/202 13.8%	848/17784 4.7%	*CXCL8 CSF1 SERPINE1 TGFA ADRA1D CXCL1 TNFRSF11A ETS1 ROBO1 FGF9 PLAU SLIT1 S1PR3 ICOSLG STAT5A FGFBP1 NOS2 TESC TNFRSF9 WNT5A PROX1 SSTR2 BTC FOSL1 NFKBIA CXCL10 FABP6 IL1B*
48519	negative regulation of biological process	4.53E-07	2.33E-05	48/202 23.7%	2020/17784 11.3%	*CD83 CXCL8 RGS16 SERPINE1 FAM129A TNFAIP3 CXCL1 ETS1 BARX2 CX3CL1 ROBO1 RND1 NPPB FGF9 PLAU UBD SLIT1 LMCD1 IER3 PACSIN1 WNT4 MBL2 STAT5A EGR1 MEF2C FGFBP1 SERPINB2 UGT1A1 TESC TNFRSF9 WNT5A PROX1 SSTR2 DUSP6 BTC FOSL1 NFKBIA CXCL10 FABP6 IL1B BCL3 SAA1 SAA2 LHX4 CRYAB NFKBID HBEGF BIRC3*
51094	positive regulation of developmental process	4.83E-07	2.45E-05	17/202 8.4%	347/17784 1.9%	*STAT5A OXTR CD83 CSF1 TESC WNT7B SERPINE1 WNT5A PROX1 ETS1 CX3CL1 ROBO1 BTC WNT6 FGF9 IL1B WNT4*
30595	leukocyte chemotaxis	5.01E-07	2.50E-05	7/202 3.4%	43/17784 0.2%	*CXCL8 IL1B SAA1 SAA2 TNFRSF11A CXCL3 CX3CL1*
7154	cell communication	5.33E-07	2.61E-05	27/202 13.3%	811/17784 4.5%	*OXTR ADRA1D TNFRSF11A AREG WNT6 FGF9 GJA10 TIFA SLIT1 NPTX1 WNT4 EGR2 FGFBP1 CCL22 CCL20 GAD1 WNT7B WNT5A FOS SSTR2 FOSL1 CXCL10 GJB2 CXCL11 SYPL1 SNPH IL1B*
50900	leukocyte migration	5.47E-07	2.64E-05	8/202 3.9%	63/17784 0.3%	*CXCL8 IL1B SAA1 SAA2 TNFRSF11A CXCL3 CX3CL1 ICAM1*
30879	mammary gland development	6.29E-07	2.93E-05	9/202 4.4%	87/17784 0.4%	*STAT5A OXTR CSF1 WNT7B WNT5A TNFRSF11A ROBO1 WNT4 APLN*

32103	positive regulation of response to external stimulus	6.29E-07	2.93E-05	9/202 4.4%	87/17784 0.4%	*STAT5A CXCL10 CXCL8 IL1B SERPINE1 WNT5A TLR7 TNFRSF11A CX3CL1*
1775	cell activation	6.38E-07	2.93E-05	15/202 7.4%	276/17784 1.5%	*STAT5A EGR1 CXCL8 CSF1 CX3CL1 ICAM1 RELB NFKB2 UBD IRF1 BCL3 SAA1 SAA2 TLR7 ICOSLG*
32879	regulation of localization	8.33E-07	3.77E-05	25/202 12.3%	728/17784 4.0%	*OXTR CSF1 LAMA4 SERPINE1 TNFRSF11A ETS1 ICAM1 PLAU SLIT1 PACSIN1 MBL2 NOS2 LGI3 WNT5A PROX1 BTC NFKBIA CXCL10 IL1B BCL3 SAA1 SAA2 TLR7 CRYAB HBEGF*
23034	intracellular signaling pathway	8.88E-07	3.96E-05	33/202 16.3%	1158/17784 6.5%	*OXTR CXCL8 RASEF TGFA ADRA1D CXCL1 GNAI1 RASGRP3 RND1 RAB43 SPRED1 IRAK2 TIFA S1PR3 CA8 IKBKE STAT5A DUSP4 VAV3 ARL14 NOS2 PRKCE WNT5A SSTR2 DUSP6 NFKBIA RCAN1 MRAS IL1B BCL3 TLR7 SOS1 CRYAB*
7267	cell-cell signaling	9.59E-07	4.22E-05	22/202 10.8%	587/17784 3.3%	*OXTR FGFBP1 CCL22 CCL20 GAD1 WNT7B ADRA1D TNFRSF11A SSTR2 AREG WNT6 CXCL10 GJB2 CXCL11 FGF9 SYPL1 SNPH IL1B TIFA SLIT1 NPTX1 WNT4*
60326	cell chemotaxis	1.09E-06	4.73E-05	7/202 3.4%	48/17784 0.2%	*CXCL8 IL1B SAA1 SAA2 TNFRSF11A CXCL3 CX3CL1*
50793	regulation of developmental process	1.12E-06	4.79E-05	26/202 12.8%	791/17784 4.4%	*OXTR CD83 CSF1 LAMA4 SERPINE1 ETS1 CX3CL1 ROBO1 NPPB WNT6 FGF9 PALMD SLIT1 WNT4 STAT5A EGR2 TESC WNT7B WNT5A PROX1 BTC NFKBIA CXCL10 IL1B MAFF NFKBID*
42573	retinoic acid metabolic process	1.37E-06	5.75E-05	5/202 2.4%	18/17784 0.1%	*ALDH1A3 DHRS9 UGT1A1 CYP1A1 AHRR*
45787	positive regulation of cell cycle	1.39E-06	5.77E-05	8/202 3.9%	71/17784 0.3%	*BTC STAT5A FOSL1 IL1B CYP1A1 TGFA AHRR PROX1*

65008	regulation of biological quality	1.59E-06	6.50E-05	39/202 19.3%	1543/17784 8.6%	*SERPINA3 OXTR DYRK3 CSF1 SERPINE1 ITPR1 TNFRSF11A NPPB PALMD PLAU SLIT1 CYP1B1 S1PR3 CCR6 WNT4 MBL2 SLC34A3 STAT5A IGFBP1 EGR1 EGR2 UGT1A1 NOS2 GAD1 WNT7B NFKBIA ALDH1A3 DHRS9 SNPH IL1B BCL3 CYP1A1 SAA1 LCN2 AHRR SAA2 TLR7 CRYAB HBEGF*
51716	cellular response to stimulus	2.55E-06	1.03E-04	29/202 14.3%	989/17784 5.5%	*CXCL8 SERPINE1 FAM129A TNFRSF11A CXCL3 CX3CL1 WNT6 IRAK2 SLIT1 IKBKE WNT4 STAT5A IGFBP1 EGR1 UGT1A1 WNT7B WNT5A FOS FOSL1 NFKBIA CXCL10 IL1B BCL3 CYP1A1 SAA1 AHRR SAA2 FSBP CRYAB*
1819	positive regulation of cytokine production	2.85E-06	1.14E-04	9/202 4.4%	104/17784 0.5%	*CD83 IL1B SERPINE1 WNT5A BCL3 SAA1 SAA2 TLR7 CX3CL1*
35466	regulation of signaling pathway	3.36E-06	1.32E-04	29/202 14.3%	1003/17784 5.6%	*CSF1 RGS16 TGFA TBC1D3F TNFAIP3 TNFRSF11A TBC1D3G ROBO1 RASGRP3 SPRED1 FGF9 IRAK2 UBD TIFA SLIT1 IKBKE LMCD1 WNT4 VAV3 FGFBP1 WNT5A DUSP6 RGMA NFKBIA IL1B SOS1 NFKBID TMEM9B HBEGF*
1817	regulation of cytokine production	3.67E-06	1.43E-04	12/202 5.9%	203/17784 1.1%	*STAT5A CD83 IL1B IRF1 SERPINE1 WNT5A BCL3 SAA1 SAA2 TLR7 S1PR3 CX3CL1*
9607	response to biotic stimulus	3.78E-06	1.45E-04	18/202 8.9%	448/17784 2.5%	*CCL22 UGT1A1 NOS2 CCL20 SERPINE1 WNT5A FOS TNFRSF11A FOSL1 NFKBIA CXCL10 IRAK2 IL1B BCL3 CYP1A1 AHRR TLR7 MBL2*
23014	signal transmission via phosphorylation event	4.32E-06	1.61E-04	16/202 7.9%	364/17784 2.0%	*STAT5A DUSP4 OXTR NOS2 WNT5A TGFA DUSP6 RASGRP3 NFKBIA SPRED1 IRAK2 IL1B BCL3 TIFA TLR7 CRYAB*
7243	intracellular protein kinase	4.32E-06	1.61E-04	16/202	364/17784	*STAT5A DUSP4 OXTR NOS2 WNT5A TGFA DUSP6*

	cascade			7.9%	2.0%	*RASGRP3 NFKBIA SPRED1 IRAK2 IL1B BCL3 TIFA TLR7 CRYAB*
31349	positive regulation of defense response	4.59E-06	1.68E-04	8/202 3.9%	83/17784 0.4%	*STAT5A NFKBIA IRAK2 IL1B SERPINE1 TLR7 TNFRSF11A CX3CL1*
7567	parturition	5.01E-06	1.80E-04	4/202 1.9%	11/17784 0.0%	*OXTR MAFF CYP1A1 AHRR*
10628	positive regulation of gene expression	5.41E-06	1.92E-04	21/202 10.3%	603/17784 3.3%	*STAT5A EGR1 MEF2C EGR2 CSF1 TESC WNT5A GRHL3 FOS PROX1 ETS1 BARX2 RELB FOSL1 NFKBIA WNT6 IL1B IRF1 BCL3 TIFA WNT4*
45750	positive regulation of S phase of mitotic cell cycle	5.73E-06	1.97E-04	3/202 1.4%	4/17784 0.0%	*CYP1A1 AHRR PROX1*
60137	maternal process involved in parturition	5.73E-06	1.97E-04	3/202 1.4%	4/17784 0.0%	*OXTR CYP1A1 AHRR*
10033	response to organic substance	6.34E-06	2.15E-04	26/202 12.8%	870/17784 4.8%	*OXTR CD83 TAT SERPINE1 TNFRSF11A IRAK2 UBD SLIT1 CYP1B1 WNT4 STAT5A DUSP4 IGFBP1 EGR1 EGR2 UGT1A1 WNT5A FOS DUSP6 FOSL1 NFKBIA CXCL10 IL1B CYP1A1 AHRR CRYAB*
50729	positive regulation of inflammatory response	6.44E-06	2.16E-04	6/202 2.9%	41/17784 0.2%	*STAT5A IL1B SERPINE1 TLR7 TNFRSF11A CX3CL1*
2682	regulation of immune system process	7.30E-06	2.42E-04	17/202 8.4%	424/17784 2.3%	*STAT5A CD83 CSF1 NOS2 TESC SERPINE1 WNT5A ETS1 ICAM1 NFKBIA CXCL10 IRAK2 IL1B TLR7 NFKBID ICOSLG MBL2*
31328	positive regulation of cellular biosynthetic process	7.82E-06	2.57E-04	23/202 11.3%	720/17784 4.0%	*STAT5A EGR1 MEF2C EGR2 TESC WNT5A FAM129A SAMD4A GRHL3 FOS PROX1 AREG ETS1 BARX2 ICAM1 FOSL1 NFKBIA IL1B IRF1 BCL3 TIFA TLR7 WNT4*

48514	blood vessel morphogenesis	8.36E-06	2.69E-04	12/202 5.9%	220/17784 1.2%	*VAV3 CXCL8 FGF9 NOS2 TIPARP PLAU WNT7B ZC3H12A TNFAIP2 PROX1 CX3CL1 ROBO1*
48583	regulation of response to stimulus	9.04E-06	2.85E-04	19/202 9.4%	525/17784 2.9%	*STAT5A CXCL8 NOS2 SERPINE1 WNT5A TNFRSF11A CX3CL1 ICAM1 ROBO1 NFKBIA CXCL10 PLAU IRAK2 IL1B SAA1 SAA2 TLR7 HBEGF MBL2*
10557	positive regulation of macromolecule biosynthetic process	9.13E-06	2.85E-04	22/202 10.8%	675/17784 3.7%	*STAT5A EGR1 MEF2C EGR2 TESC WNT5A FAM129A SAMD4A GRHL3 FOS PROX1 AREG ETS1 BARX2 FOSL1 NFKBIA IL1B IRF1 BCL3 TIFA TLR7 WNT4*
50727	regulation of inflammatory response	9.15E-06	2.85E-04	8/202 3.9%	91/17784 0.5%	*STAT5A IL1B SERPINE1 SAA1 SAA2 TLR7 TNFRSF11A CX3CL1*
90068	positive regulation of cell cycle process	9.82E-06	3.03E-04	6/202 2.9%	44/17784 0.2%	*BTC IL1B CYP1A1 TGFA AHRR PROX1*
9891	positive regulation of biosynthetic process	9.99E-06	3.05E-04	23/202 11.3%	731/17784 4.1%	*STAT5A EGR1 MEF2C EGR2 TESC WNT5A FAM129A SAMD4A GRHL3 FOS PROX1 AREG ETS1 BARX2 ICAM1 FOSL1 NFKBIA IL1B IRF1 BCL3 TIFA TLR7 WNT4*
51704	multi-organism process	1.04E-05	3.14E-04	24/202 11.8%	786/17784 4.4%	*STAT5A OXTR CCL22 UGT1A1 NOS2 CCL20 SERPINE1 WNT5A FOS TNFRSF11A ICAM1 FOSL1 NFKBIA CXCL10 PLAU IRAK2 IL1B BCL3 MAFF CYP1A1 AHRR TLR7 WNT4 MBL2*
34754	cellular hormone metabolic process	1.07E-05	3.18E-04	7/202 3.4%	67/17784 0.3%	*ALDH1A3 DHRS9 UGT1A1 CYP1A1 CYP1B1 AHRR WNT4*
1568	blood vessel development	1.10E-05	3.20E-04	13/202 6.4%	265/17784 1.4%	*VAV3 CXCL8 NOS2 TIPARP WNT7B LAMA4 TNFAIP2 PROX1 CX3CL1 ROBO1 FGF9 PLAU ZC3H12A*

2684	positive regulation of immune system process	1.10E-05	3.20E-04	13/202 6.4%	265/17784 1.4%	*STAT5A CD83 NOS2 SERPINE1 WNT5A ICAM1 NFKBIA CXCL10 IRAK2 IL1B TLR7 ICOSLG MBL2*
48584	positive regulation of response to stimulus	1.24E-05	3.57E-04	13/202 6.4%	268/17784 1.5%	*STAT5A CXCL8 SERPINE1 WNT5A TNFRSF11A CX3CL1 NFKBIA CXCL10 IRAK2 IL1B TLR7 HBEGF MBL2*
70365	hepatocyte differentiation	1.42E-05	3.89E-04	3/202 1.4%	5/17784 0.0%	*CYP1A1 AHRR PROX1*
42904	9-cis-retinoic acid biosynthetic process	1.42E-05	3.89E-04	3/202 1.4%	5/17784 0.0%	*DHRS9 CYP1A1 AHRR*
42905	9-cis-retinoic acid metabolic process	1.42E-05	3.89E-04	3/202 1.4%	5/17784 0.0%	*DHRS9 CYP1A1 AHRR*
35238	vitamin A biosynthetic process	1.42E-05	3.89E-04	3/202 1.4%	5/17784 0.0%	*DHRS9 CYP1A1 AHRR*
60900	embryonic camera-type eye formation	1.42E-05	3.89E-04	3/202 1.4%	5/17784 0.0%	*ALDH1A3 WNT5A PROX1*
45321	leukocyte activation	1.49E-05	4.05E-04	12/202 5.9%	233/17784 1.3%	*STAT5A EGR1 CXCL8 CSF1 UBD IRF1 BCL3 TLR7 ICOSLG CX3CL1 ICAM1 RELB*
1944	vasculature development	1.51E-05	4.07E-04	13/202 6.4%	273/17784 1.5%	*VAV3 CXCL8 NOS2 TIPARP WNT7B LAMA4 TNFAIP2 PROX1 CX3CL1 ROBO1 FGF9 PLAU ZC3H12A*
31347	regulation of defense response	1.65E-05	4.42E-04	10/202 4.9%	162/17784 0.9%	*STAT5A NFKBIA IRAK2 IL1B SERPINE1 SAA1 SAA2 TLR7 TNFRSF11A CX3CL1*
48523	negative regulation of cellular process	1.91E-05	5.04E-04	41/202 20.2%	1843/17784 10.3%	*CXCL8 RGS16 SERPINE1 FAM129A TNFAIP3 CXCL1 ETS1 BARX2 CX3CL1 ROBO1 RND1 NPPB FGF9 UBD SLIT1 LMCD1 IER3 PACSIN1 WNT4*

						STAT5A EGR1 MEF2C FGFBP1 SERPINB2 TESC TNFRSF9 WNT5A PROX1 SSTR2 DUSP6 BTC FOSL1 NFKBIA FABP6 IL1B BCL3 LHX4 CRYAB NFKBID HBEGF BIRC3
7166	cell surface receptor linked signaling pathway	1.99E-05	5.20E-04	32/202 15.8%	1280/17784 7.1%	*OXTR CXCL8 NXPH3 ADRA1D CXCL1 TNFRSF11A AREG CX3CL1 GNAI1 ROBO1 NPPB WNT6 FGF9 IRAK2 SLIT1 S1PR3 WNT4 STAT5A VAV3 IGFBP1 NOS2 TIPARP WNT7B WNT5A FOS SSTR2 NFKBIA CXCL10 IL1B SOS1 HBEGF BIRC3*
1523	retinoid metabolic process	2.04E-05	5.20E-04	5/202 2.4%	30/17784 0.1%	*ALDH1A3 DHRS9 UGT1A1 CYP1A1 AHRR*
6776	vitamin A metabolic process	2.04E-05	5.20E-04	5/202 2.4%	30/17784 0.1%	*ALDH1A3 DHRS9 UGT1A1 CYP1A1 AHRR*
16101	diterpenoid metabolic process	2.04E-05	5.20E-04	5/202 2.4%	30/17784 0.1%	*ALDH1A3 DHRS9 UGT1A1 CYP1A1 AHRR*
42108	positive regulation of cytokine biosynthetic process	2.35E-05	5.94E-04	6/202 2.9%	51/17784 0.2%	*STAT5A IL1B IRF1 WNT5A BCL3 TLR7*
43066	negative regulation of apoptosis	2.71E-05	6.81E-04	15/202 7.4%	376/17784 2.1%	*STAT5A MEF2C SERPINB2 SERPINE1 WNT5A TNFAIP3 CX3CL1 BTC NFKBIA IL1B BCL3 LHX4 CRYAB IER3 BIRC3*
42362	fat-soluble vitamin biosynthetic process	2.82E-05	7.01E-04	3/202 1.4%	6/17784 0.0%	*DHRS9 CYP1A1 AHRR*
34097	response to cytokine stimulus	3.01E-05	7.43E-04	8/202 3.9%	107/17784 0.6%	*FOSL1 OXTR IRAK2 UBD SERPINE1 WNT5A FOS TNFRSF11A*
6928	cellular component movement	3.06E-05	7.49E-04	17/202 8.4%	474/17784 2.6%	*VAV3 ROBO3 EGR2 CCL22 CXCL8 TNFRSF11A CXCL3 ETS1 CX3CL1 ICAM1 ROBO1 IL1B SAA1*

						SLIT1 SAA2 CCR6 LHX4
43069	negative regulation of programmed cell death	3.16E-05	7.67E-04	15/202 7.4%	381/17784 2.1%	*STAT5A MEF2C SERPINB2 SERPINE1 WNT5A TNFAIP3 CX3CL1 BTC NFKBIA IL1B BCL3 LHX4 CRYAB IER3 BIRC3*
71222	cellular response to lipopolysaccharide	3.42E-05	8.26E-04	4/202 1.9%	17/17784 0.0%	*NFKBIA IRAK2 SERPINE1 WNT5A*
9893	positive regulation of metabolic process	3.50E-05	8.37E-04	27/202 13.3%	1018/17784 5.7%	*CSF1 FAM129A AREG ETS1 BARX2 ICAM1 RELB WNT6 TIFA WNT4 STAT5A VAV3 EGR1 MEF2C EGR2 TESC WNT5A SAMD4A GRHL3 FOS PROX1 FOSL1 NFKBIA IL1B IRF1 BCL3 TLR7*
60548	negative regulation of cell death	4.00E-05	9.50E-04	15/202 7.4%	389/17784 2.1%	*STAT5A MEF2C SERPINB2 SERPINE1 WNT5A TNFAIP3 CX3CL1 BTC NFKBIA IL1B BCL3 LHX4 CRYAB IER3 BIRC3*
48589	developmental growth	4.18E-05	9.83E-04	8/202 3.9%	112/17784 0.6%	*IGFBP1 CSF1 PLAU WNT7B SERPINE1 WNT5A SLIT1 ROBO1*
48534	hemopoietic or lymphoid organ development	4.22E-05	9.87E-04	12/202 5.9%	259/17784 1.4%	*STAT5A EGR1 DYRK3 CSF1 TIPARP UBD IRF1 WNT5A BCL3 TNFRSF11A RELB NFKB2*
9653	anatomical structure morphogenesis	4.85E-05	1.11E-03	30/202 14.8%	1217/17784 6.8%	*ROBO3 CXCL8 CSF1 SERPINE1 TNFAIP2 BARX2 CX3CL1 ROBO1 NPPB WNT6 FGF9 PLAU ZC3H12A SLIT1 S1PR3 IER3 WNT4 STAT5A VAV3 IGFBP1 EGR2 NOS2 TIPARP WNT7B WNT5A PROX1 RGMA ALDH1A3 LHX4 CRYAB*
42537	benzene and derivative metabolic process	4.89E-05	1.11E-03	3/202 1.4%	7/17784 0.0%	*UGT1A1 CYP1A1 AHRR*
48247	lymphocyte chemotaxis	4.89E-05	1.11E-03	3/202 1.4%	7/17784 0.0%	*SAA1 SAA2 CX3CL1*

61180	mammary gland epithelium development	5.09E-05	1.15E-03	5/202 2.4%	36/17784 0.2%	*CSF1 WNT7B WNT5A ROBO1 WNT4*
9991	response to extracellular stimulus	5.27E-05	1.18E-03	12/202 5.9%	265/17784 1.4%	*SLC34A3 FOSL1 WNT6 CXCL10 UGT1A1 IL1B WNT7B WNT5A CYP1A1 AHRR FOS SSTR2*
60560	developmental growth involved in morphogenesis	5.48E-05	1.22E-03	4/202 1.9%	19/17784 0.1%	*CSF1 WNT7B WNT5A ROBO1*
14070	response to organic cyclic substance	5.50E-05	1.22E-03	9/202 4.4%	150/17784 0.8%	*FOSL1 OXTR CD83 IL1B TAT CYP1A1 AHRR FOS DUSP6*
30154	cell differentiation	5.61E-05	1.23E-03	37/202 18.3%	1668/17784 9.3%	*ROBO3 DYRK3 CSF1 LAMA4 TNFAIP2 TNFRSF11A BARX2 ROBO1 RELB RND1 WNT6 FGF9 UBD ZC3H12A SLIT1 WNT4 STAT5A EGR1 MEF2C EGR2 NOS2 TIPARP WNT7B WNT5A FOXN4 PROX1 DUSP6 NFKB2 SLC7A5 RCAN1 DHRS9 IRF1 BCL3 CYP1A1 AHRR LHX4 CRYAB*
6721	terpenoid metabolic process	6.66E-05	1.43E-03	5/202 2.4%	38/17784 0.2%	*ALDH1A3 DHRS9 UGT1A1 CYP1A1 AHRR*
51674	localization of cell	6.67E-05	1.43E-03	13/202 6.4%	315/17784 1.7%	*VAV3 CCL22 CXCL8 TNFRSF11A CXCL3 ETS1 CX3CL1 ICAM1 ROBO1 IL1B SAA1 SLIT1 SAA2*
48870	cell motility	6.67E-05	1.43E-03	13/202 6.4%	315/17784 1.7%	*VAV3 CCL22 CXCL8 TNFRSF11A CXCL3 ETS1 CX3CL1 ICAM1 ROBO1 IL1B SAA1 SLIT1 SAA2*
71219	cellular response to molecule of bacterial origin	6.79E-05	1.44E-03	4/202 1.9%	20/17784 0.1%	*NFKBIA IRAK2 SERPINE1 WNT5A*
10604	positive regulation of macromolecule metabolic process	6.80E-05	1.44E-03	25/202 12.3%	941/17784 5.2%	*CSF1 FAM129A AREG ETS1 BARX2 RELB WNT6 TIFA WNT4 STAT5A EGR1 MEF2C EGR2 TESC WNT5A SAMD4A GRHL3 FOS PROX1 FOSL1 NFKBIA IL1B IRF1 BCL3 TLR7*

19748	secondary metabolic process	7.22E-05	1.52E-03	6/202<br>2.9%	62/17784<br>0.3%	*ALDH1A3 DHRS9 UGT1A1 CYP1A1 CYP1B1 AHRR*
48732	gland development	7.37E-05	1.54E-03	10/202<br>4.9%	193/17784<br>1.0%	*STAT5A ALDH1A3 OXTR CSF1 WNT7B WNT5A TNFRSF11A ROBO1 WNT4 APLN*
30097	hemopoiesis	7.47E-05	1.55E-03	11/202<br>5.4%	233/17784<br>1.3%	*STAT5A EGR1 DYRK3 CSF1 TIPARP UBD IRF1 WNT5A BCL3 TNFRSF11A RELB*
2520	immune system development	7.52E-05	1.55E-03	12/202<br>5.9%	275/17784<br>1.5%	*STAT5A EGR1 DYRK3 CSF1 TIPARP UBD IRF1 WNT5A BCL3 TNFRSF11A RELB NFKB2*
6953	acute-phase response	7.57E-05	1.55E-03	5/202<br>2.4%	39/17784<br>0.2%	*SERPINA3 TIFA SAA1 SAA2 MBL2*
9404	toxin metabolic process	7.75E-05	1.57E-03	3/202<br>1.4%	8/17784<br>0.0%	*CYP1A1 CYP1B1 AHRR*
80134	regulation of response to stress	7.82E-05	1.57E-03	13/202<br>6.4%	320/17784<br>1.7%	*STAT5A SERPINE1 WNT5A TNFRSF11A CX3CL1 NFKBIA PLAU IRAK2 IL1B SAA1 SAA2 TLR7 HBEGF*
51101	regulation of DNA binding	7.83E-05	1.57E-03	9/202<br>4.4%	157/17784<br>0.8%	*NFKBIA IRAK2 IL1B WNT5A BCL3 PROX1 TNFRSF11A NFKBID ICAM1*
7223	Wnt receptor signaling pathway, calcium modulating pathway	8.31E-05	1.66E-03	4/202<br>1.9%	21/17784<br>0.1%	*WNT6 WNT7B WNT5A WNT4*
6775	fat-soluble vitamin metabolic process	8.57E-05	1.70E-03	5/202<br>2.4%	40/17784<br>0.2%	*ALDH1A3 DHRS9 UGT1A1 CYP1A1 AHRR*
51173	positive regulation of nitrogen compound metabolic process	9.33E-05	1.83E-03	20/202<br>9.9%	677/17784<br>3.8%	*STAT5A EGR1 MEF2C EGR2 TESC WNT5A GRHL3 FOS PROX1 AREG ETS1 BARX2 ICAM1 FOSL1 NFKBIA IL1B IRF1 BCL3 TIFA WNT4*

51270	regulation of cellular component movement	9.38E-05	1.83E-03	11/202 5.4%	239/17784 1.3%	*CXCL10 CSF1 PLAU LAMA4 SERPINE1 WNT5A SLIT1 PROX1 ETS1 ICAM1 HBEGF*
6916	anti-apoptosis	9.51E-05	1.84E-03	10/202 4.9%	199/17784 1.1%	*STAT5A NFKBIA MEF2C SERPINB2 IL1B TNFAIP3 LHX4 CRYAB IER3 BIRC3*
16477	cell migration	9.55E-05	1.84E-03	12/202 5.9%	282/17784 1.5%	*VAV3 CCL22 CXCL8 IL1B SAA1 SLIT1 SAA2 TNFRSF11A CXCL3 CX3CL1 ICAM1 ROBO1*
48869	cellular developmental process	9.89E-05	1.87E-03	37/202 18.3%	1714/17784 9.6%	*ROBO3 DYRK3 CSF1 LAMA4 TNFAIP2 TNFRSF11A BARX2 ROBO1 RELB RND1 WNT6 FGF9 UBD ZC3H12A SLIT1 WNT4 STAT5A EGR1 MEF2C EGR2 NOS2 TIPARP WNT7B WNT5A FOXN4 PROX1 DUSP6 NFKB2 SLC7A5 RCAN1 DHRS9 IRF1 BCL3 CYP1A1 AHRR LHX4 CRYAB*
42060	wound healing	9.91E-05	1.87E-03	10/202 4.9%	200/17784 1.1%	*IGFBP1 SERPINB2 PLAU IL1B SERPINE1 WNT5A SAA1 SAA2 GRHL3 CX3CL1*
45931	positive regulation of mitotic cell cycle	1.01E-04	1.87E-03	4/202 1.9%	22/17784 0.1%	*STAT5A CYP1A1 AHRR PROX1*
35567	non-canonical Wnt receptor signaling pathway	1.01E-04	1.87E-03	4/202 1.9%	22/17784 0.1%	*WNT6 WNT7B WNT5A WNT4*
60603	mammary gland duct morphogenesis	1.01E-04	1.87E-03	4/202 1.9%	22/17784 0.1%	*CSF1 WNT5A ROBO1 WNT4*
40012	regulation of locomotion	1.01E-04	1.87E-03	11/202 5.4%	241/17784 1.3%	*CXCL10 CXCL8 CSF1 PLAU LAMA4 SERPINE1 WNT5A PROX1 ICAM1 ROBO1 HBEGF*
2521	leukocyte differentiation	1.02E-04	1.88E-03	8/202 3.9%	127/17784 0.7%	*STAT5A EGR1 CSF1 UBD IRF1 BCL3 TNFRSF11A RELB*
48646	anatomical structure	1.02E-04	1.88E-03	14/202	375/17784	*VAV3 EGR2 CXCL8 WNT5A TNFAIP2 PROX1*

	formation involved in morphogenesis			6.9%	2.1%	*CX3CL1 ROBO1 RGMA ALDH1A3 FGF9 PLAU ZC3H12A WNT4*
45941	positive regulation of transcription	1.03E-04	1.88E-03	18/202 8.9%	575/17784 3.2%	*STAT5A EGR1 MEF2C EGR2 TESC WNT5A GRHL3 FOS PROX1 ETS1 BARX2 FOSL1 NFKBIA IL1B IRF1 BCL3 TIFA WNT4*
8285	negative regulation of cell proliferation	1.14E-04	2.07E-03	14/202 6.9%	379/17784 2.1%	*FGFBP1 CXCL8 TESC TNFRSF9 WNT5A CXCL1 PROX1 SSTR2 ETS1 ROBO1 FOSL1 FABP6 IL1B SLIT1*
51384	response to glucocorticoid stimulus	1.19E-04	2.14E-03	7/202 3.4%	97/17784 0.5%	*FOSL1 UGT1A1 IL1B TAT SERPINE1 SLIT1 FOS*
17143	insecticide metabolic process	1.28E-04	2.22E-03	2/202 0.9%	2/17784 0.0%	*CYP1A1 AHRR*
9635	response to herbicide	1.28E-04	2.22E-03	2/202 0.9%	2/17784 0.0%	*CYP1A1 AHRR*
2266	follicular dendritic cell activation	1.28E-04	2.22E-03	2/202 0.9%	2/17784 0.0%	*BCL3 NFKB2*
2268	follicular dendritic cell differentiation	1.28E-04	2.22E-03	2/202 0.9%	2/17784 0.0%	*BCL3 NFKB2*
19341	dibenzo-p-dioxin catabolic process	1.28E-04	2.22E-03	2/202 0.9%	2/17784 0.0%	*CYP1A1 AHRR*
22414	reproductive process	1.35E-04	2.32E-03	22/202 10.8%	808/17784 4.5%	*STAT5A OXTR CSF1 NOS2 WNT5A FOS ICAM1 APLN FOSL1 GJB2 DNMT3L FGF9 PLAU GJA10 IL1B MAFF CYP1A1 SLIT1 AHRR TRIM36 WNT4 BIRC3*
7584	response to nutrient	1.37E-04	2.35E-03	9/202	169/17784	*WNT6 CXCL10 UGT1A1 IL1B WNT7B WNT5A*

				4.4%	0.9%	*CYP1A1 AHRR SSTR2*
3	reproduction	1.42E-04	2.41E-03	22/202<br>10.8%	811/17784<br>4.5%	*STAT5A OXTR CSF1 NOS2 WNT5A FOS ICAM1 APLN FOSL1 GJB2 DNMT3L FGF9 PLAU GJA10 IL1B MAFF CYP1A1 SLIT1 AHRR TRIM36 WNT4 BIRC3*
45785	positive regulation of cell adhesion	1.43E-04	2.41E-03	6/202<br>2.9%	70/17784<br>0.3%	*VAV3 CSF1 IL1B SAA1 SAA2 CX3CL1*
2250	adaptive immune response	1.55E-04	2.60E-03	6/202<br>2.9%	71/17784<br>0.3%	*BCL3 TNFRSF11A ICAM1 RELB NFKB2 MBL2*
51090	regulation of transcription factor activity	1.64E-04	2.72E-03	8/202<br>3.9%	136/17784<br>0.7%	*NFKBIA IRAK2 IL1B WNT5A PROX1 TNFRSF11A NFKBID ICAM1*
90046	regulation of transcription regulator activity	1.64E-04	2.72E-03	8/202<br>3.9%	136/17784<br>0.7%	*NFKBIA IRAK2 IL1B WNT5A PROX1 TNFRSF11A NFKBID ICAM1*
9987	cellular process	1.66E-04	2.74E-03	132/202<br>65.3%	9368/17784<br>52.6%	*OXTR CSF1 SERPINE1 ETS1 ICAM1 NPPB FGF9 ZC3H12A CYP1B1 PHLDA1 ICOSLG IER3 SLC34A3 IGFBP1 MEF2C UNC13A SNRPN PRKCE WNT5A C8ORF4 RNASE2 TUBA4A ALDH3A1 SYPL1 ALPK3 ATP6V0D2 SLC45A4 ITPR1 TGFA DAPP1 TNFRSF11A ARL17B WNT6 IRAK2 GJA10 UBD NPTX1 PACSIN1 WNT4 STAT5A EGR1 KCNF1 EGR2 PSAPL1 TIPARP FOXN4 FHDC1 LSM4 NFKB2 FOSL1 NFKBIA RCAN1 CXCL10 GJB2 CXCL11 BCL3 MAFF CYP1A1 LCN2 LHX4 ROBO3 DYRK3 CXCL8 TAT TNFAIP2 TNFAIP3 ADRA1D CXCL1 CXCL3 AREG SLC2A6 CX3CL1 ROBO1 RND1 DNMT3L ACOXL OLR1 PGM5 CCR6 CA8 IKBKE MBL2 DUSP4 VAV3 HS3ST3B1 FGFBP1 UGT1A1 SLC2A10 PCDHGA3 HUNK FOS PROX1 SSTR2 DUSP6 SLC7A5 ALDH1A3 MRAS SNPH IL1B*

						IRF1 CDH11 AHRR TLR7 MYH4 SOS1 CRYAB VAMP2 LAMA4 FAM129A GDPD1 ATP10B BARX2 STX11 RELB TIFA STX6 SLIT1 TDH S1PR3 CCL22 NDUFA5 NOS2 CCL20 GAD1 WNT7B SGPP2 DHRS9 SAA1 ATP13A4 SAA2 TRIM36 FSBP
51272	positive regulation of cellular component movement	1.73E-04	2.82E-03	8/202 3.9%	137/17784 0.7%	*CXCL10 CSF1 SERPINE1 WNT5A PROX1 ETS1 ICAM1 HBEGF*
40017	positive regulation of locomotion	1.73E-04	2.82E-03	8/202 3.9%	137/17784 0.7%	*CXCL10 CXCL8 CSF1 SERPINE1 WNT5A PROX1 ICAM1 HBEGF*
33273	response to vitamin	1.73E-04	2.82E-03	7/202 3.4%	103/17784 0.5%	*WNT6 CXCL10 IL1B WNT7B WNT5A CYP1A1 AHRR*
22603	regulation of anatomical structure morphogenesis	1.76E-04	2.84E-03	12/202 5.9%	301/17784 1.6%	*NPPB WNT6 CXCL10 CSF1 PALMD IL1B SERPINE1 WNT5A SLIT1 PROX1 CX3CL1 ROBO1*
42493	response to drug	1.78E-04	2.85E-03	11/202 5.4%	257/17784 1.4%	*FOSL1 ALDH1A3 OXTR UGT1A1 IL1B CYP1A1 AHRR FSBP FOS DUSP6 ABCG2*
45935	positive regulation of nucleobase, nucleoside, nucleotide and nucleic acid metabolic process	1.81E-04	2.89E-03	19/202 9.4%	656/17784 3.6%	*STAT5A EGR1 MEF2C EGR2 TESC WNT5A GRHL3 FOS PROX1 AREG ETS1 BARX2 FOSL1 NFKBIA IL1B IRF1 BCL3 TIFA WNT4*
31960	response to corticosteroid stimulus	1.84E-04	2.93E-03	7/202 3.4%	104/17784 0.5%	*FOSL1 UGT1A1 IL1B TAT SERPINE1 SLIT1 FOS*
33628	regulation of cell adhesion mediated by integrin	2.23E-04	3.49E-03	3/202 1.4%	11/17784 0.0%	*PLAU TESC SERPINE1*
9812	flavonoid metabolic process	2.23E-04	3.49E-03	3/202 1.4%	11/17784 0.0%	*UGT1A1 CYP1A1 AHRR*

48246	macrophage chemotaxis	2.23E-04	3.49E-03	3/202 1.4%	11/17784 0.0%	*SAA1 SAA2 CX3CL1*
51302	regulation of cell division	2.29E-04	3.54E-03	5/202 2.4%	49/17784 0.2%	*BTC FGF9 IL1B TGFA CABLES1*
2274	myeloid leukocyte activation	2.29E-04	3.54E-03	5/202 2.4%	49/17784 0.2%	*CXCL8 CSF1 UBD TLR7 RELB*
61061	muscle structure development	2.31E-04	3.57E-03	11/202 5.4%	265/17784 1.4%	*RCAN1 CXCL10 MEF2C MEF2B MRAS TIPARP PROX1 CRYAB BARX2 HBEGF WNT4*
42035	regulation of cytokine biosynthetic process	2.42E-04	3.71E-03	6/202 2.9%	77/17784 0.4%	*STAT5A IL1B IRF1 WNT5A BCL3 TLR7*
8284	positive regulation of cell proliferation	2.50E-04	3.81E-03	15/202 7.4%	459/17784 2.5%	*STAT5A FGFBP1 CSF1 WNT5A TGFA ADRA1D PROX1 TNFRSF11A BTC FOSL1 CXCL10 FGF9 IL1B S1PR3 ICOSLG*
31325	positive regulation of cellular metabolic process	2.63E-04	3.99E-03	24/202 11.8%	966/17784 5.4%	*STAT5A EGR1 MEF2C EGR2 CSF1 TESC WNT5A FAM129A SAMD4A GRHL3 FOS PROX1 AREG ETS1 BARX2 ICAM1 FOSL1 NFKBIA IL1B IRF1 BCL3 TIFA TLR7 WNT4*
51049	regulation of transport	2.68E-04	4.05E-03	16/202 7.9%	514/17784 2.8%	*OXTR NOS2 LGI3 SERPINE1 WNT5A TNFRSF11A BTC NFKBIA IL1B BCL3 SAA1 SAA2 TLR7 CRYAB PACSIN1 MBL2*
42445	hormone metabolic process	2.75E-04	4.13E-03	7/202 3.4%	111/17784 0.6%	*ALDH1A3 DHRS9 UGT1A1 CYP1A1 CYP1B1 AHRR WNT4*
50920	regulation of chemotaxis	2.77E-04	4.13E-03	5/202 2.4%	51/17784 0.2%	*CXCL10 CXCL8 SERPINE1 WNT5A ROBO1*
10647	positive regulation of cell	2.78E-04	4.13E-03	14/202	413/17784	*OXTR FGFBP1 CSF1 WNT5A TGFA TNFRSF11A*

	communication			6.9%	2.3%	*FGF9 IL1B UBD IKBKE LMCD1 TMEM9B HBEGF WNT4*
51050	positive regulation of transport	2.81E-04	4.15E-03	11/202 5.4%	271/17784 1.5%	*BTC NFKBIA OXTR IL1B SERPINE1 WNT5A SAA1 SAA2 TLR7 TNFRSF11A MBL2*
165	MAPKKK cascade	2.82E-04	4.15E-03	9/202 4.4%	186/17784 1.0%	*DUSP4 OXTR SPRED1 IL1B WNT5A TGFA CRYAB DUSP6 RASGRP3*
45939	negative regulation of steroid metabolic process	2.95E-04	4.29E-03	3/202 1.4%	12/17784 0.0%	*UGT1A1 PROX1 WNT4*
8045	motor axon guidance	2.95E-04	4.29E-03	3/202 1.4%	12/17784 0.0%	*EGR2 SLIT1 LHX4*
7204	elevation of cytosolic calcium ion concentration	3.07E-04	4.46E-03	7/202 3.4%	113/17784 0.6%	*OXTR IL1B SAA1 ITPR1 SAA2 S1PR3 CCR6*
10627	regulation of intracellular protein kinase cascade	3.35E-04	4.84E-03	12/202 5.9%	323/17784 1.8%	*FGF9 IL1B UBD WNT5A TIFA TNFAIP3 TNFRSF11A IKBKE NFKBID TMEM9B DUSP6 HBEGF*
1525	angiogenesis	3.50E-04	5.00E-03	8/202 3.9%	152/17784 0.8%	*VAV3 CXCL8 FGF9 PLAU ZC3H12A TNFAIP2 CX3CL1 ROBO1*
60443	mammary gland morphogenesis	3.51E-04	5.00E-03	4/202 1.9%	30/17784 0.1%	*CSF1 WNT5A ROBO1 WNT4*
9888	tissue development	3.60E-04	5.10E-03	20/202 9.9%	750/17784 4.2%	*STAT5A IGFBP1 CSF1 TIPARP WNT7B SERPINE1 WNT5A GRHL3 PROX1 IKZF3 BARX2 ROBO1 RGMA RCAN1 ALDH1A3 KRT17 DHRS9 FGF9 PLAU WNT4*
9698	phenylpropanoid metabolic process	3.80E-04	5.17E-03	3/202 1.4%	13/17784 0.0%	*UGT1A1 CYP1A1 AHRR*

40036	regulation of fibroblast growth factor receptor signaling pathway	3.80E-04	5.17E-03	3/202 1.4%	13/17784 0.0%	*FGFBP1 WNT5A WNT4*
48596	embryonic camera-type eye morphogenesis	3.80E-04	5.17E-03	3/202 1.4%	13/17784 0.0%	*ALDH1A3 WNT5A PROX1*
70099	regulation of chemokine-mediated signaling pathway	3.82E-04	5.17E-03	2/202 0.9%	3/17784 0.0%	*SLIT1 ROBO1*
70100	negative regulation of chemokine-mediated signaling pathway	3.82E-04	5.17E-03	2/202 0.9%	3/17784 0.0%	*SLIT1 ROBO1*
42178	xenobiotic catabolic process	3.82E-04	5.17E-03	2/202 0.9%	3/17784 0.0%	*CYP1A1 AHRR*
10041	response to iron(III) ion	3.82E-04	5.17E-03	2/202 0.9%	3/17784 0.0%	*CYP1A1 AHRR*
2072	optic cup morphogenesis involved in camera-type eye development	3.82E-04	5.17E-03	2/202 0.9%	3/17784 0.0%	*ALDH1A3 WNT5A*
18894	dibenzo-p-dioxin metabolic process	3.82E-04	5.17E-03	2/202 0.9%	3/17784 0.0%	*CYP1A1 AHRR*
35385	Roundabout signaling pathway	3.82E-04	5.17E-03	2/202 0.9%	3/17784 0.0%	*SLIT1 ROBO1*
51092	positive regulation of NF-kappaB transcription factor activity	3.95E-04	5.29E-03	5/202 2.4%	55/17784 0.3%	*IRAK2 IL1B WNT5A TNFRSF11A ICAM1*
48565	digestive tract development	3.95E-04	5.29E-03	5/202	55/17784	*OXTR FGF9 WNT5A CYP1A1 AHRR*

				2.4%	0.3%	
2687	positive regulation of leukocyte migration	4.00E-04	5.33E-03	4/202 1.9%	31/17784 0.1%	*CXCL10 SERPINE1 WNT5A ICAM1*
10035	response to inorganic substance	4.17E-04	5.54E-03	10/202 4.9%	239/17784 1.3%	*SLC34A3 FOSL1 TAT SERPINE1 WNT5A CYP1A1 OLR1 AHRR FOS CRYAB*
35468	positive regulation of signaling pathway	4.20E-04	5.54E-03	13/202 6.4%	380/17784 2.1%	*FGFBP1 CSF1 WNT5A TGFA TNFRSF11A FGF9 IL1B UBD IKBKE LMCD1 TMEM9B HBEGF WNT4*
42110	T cell activation	4.21E-04	5.54E-03	7/202 3.4%	119/17784 0.6%	*STAT5A EGR1 IRF1 BCL3 ICOSLG ICAM1 RELB*
65009	regulation of molecular function	4.37E-04	5.72E-03	25/202 12.3%	1062/17784 5.9%	*CSF1 SERPINE1 TGFA TBC1D3F TNFRSF11A GNAI1 TBC1D3G ICAM1 ROBO1 RASGRP3 SPRED1 PLAU IRAK2 S1PR3 VAV3 WNT5A PROX1 SSTR2 DUSP6 NFKBIA RCAN1 IL1B BCL3 CRYAB NFKBID*
43010	camera-type eye development	4.43E-04	5.78E-03	7/202 3.4%	120/17784 0.6%	*ALDH1A3 WNT7B WNT5A CYP1A1 AHRR PROX1 CRYAB*
45765	regulation of angiogenesis	4.69E-04	6.10E-03	6/202 2.9%	87/17784 0.4%	*NPPB CXCL10 IL1B SERPINE1 WNT5A CX3CL1*
2286	T cell activation involved in immune response	4.79E-04	6.17E-03	3/202 1.4%	14/17784 0.0%	*BCL3 ICAM1 RELB*
51098	regulation of binding	4.79E-04	6.17E-03	9/202 4.4%	200/17784 1.1%	*NFKBIA IRAK2 IL1B WNT5A BCL3 PROX1 TNFRSF11A NFKBID ICAM1*
7565	female pregnancy	4.89E-04	6.27E-03	7/202 3.4%	122/17784 0.6%	*STAT5A FOSL1 OXTR PLAU IL1B FOS WNT4*

31667	response to nutrient levels	4.91E-04	6.27E-03	10/202 4.9%	244/17784 1.3%	*SLC34A3 WNT6 CXCL10 UGT1A1 IL1B WNT7B WNT5A CYP1A1 AHRR SSTR2*
45944	positive regulation of transcription from RNA polymerase II promoter	5.11E-04	6.47E-03	13/202 6.4%	388/17784 2.1%	*STAT5A EGR1 MEF2C EGR2 FOS ETS1 BARX2 FOSL1 NFKBIA IL1B IRF1 BCL3 TIFA*
51480	cytosolic calcium ion homeostasis	5.14E-04	6.48E-03	7/202 3.4%	123/17784 0.6%	*OXTR IL1B SAA1 ITPR1 SAA2 S1PR3 CCR6*
9719	response to endogenous stimulus	5.29E-04	6.64E-03	15/202 7.4%	493/17784 2.7%	*STAT5A IGFBP1 EGR1 OXTR EGR2 UGT1A1 TAT SERPINE1 WNT5A FOS FOSL1 IL1B SLIT1 CRYAB WNT4*
43065	positive regulation of apoptosis	5.47E-04	6.82E-03	14/202 6.9%	442/17784 2.4%	*VAV3 PRKCE TNFRSF9 ETS1 DUSP6 FOSL1 ALDH1A3 IL1B UBD BCL3 SOS1 IKBKE PHLDA1 NFKBID*
6720	isoprenoid metabolic process	5.48E-04	6.82E-03	5/202 2.4%	59/17784 0.3%	*ALDH1A3 DHRS9 UGT1A1 CYP1A1 AHRR*
2088	lens development in camera-type eye	5.74E-04	7.09E-03	4/202 1.9%	34/17784 0.1%	*WNT7B WNT5A PROX1 CRYAB*
43068	positive regulation of programmed cell death	5.84E-04	7.19E-03	14/202 6.9%	445/17784 2.5%	*VAV3 PRKCE TNFRSF9 ETS1 DUSP6 FOSL1 ALDH1A3 IL1B UBD BCL3 SOS1 IKBKE PHLDA1 NFKBID*
46632	alpha-beta T cell differentiation	5.94E-04	7.22E-03	3/202 1.4%	15/17784 0.0%	*IRF1 BCL3 RELB*
2224	toll-like receptor signaling pathway	5.94E-04	7.22E-03	3/202 1.4%	15/17784 0.0%	*NFKBIA IRAK2 TLR7*
60444	branching involved in	5.94E-04	7.22E-03	3/202	15/17784	*CSF1 WNT5A WNT4*

	mammary gland duct morphogenesis			1.4%	0.0%	
45595	regulation of cell differentiation	5.97E-04	7.22E-03	16/202 7.9%	553/17784 3.1%	*STAT5A CD83 CSF1 TESC WNT7B WNT5A PROX1 ETS1 ROBO1 BTC NFKBIA FGF9 MAFF SLIT1 NFKBID WNT4*
45893	positive regulation of transcription, DNA-dependent	6.11E-04	7.37E-03	15/202 7.4%	500/17784 2.8%	*STAT5A EGR1 MEF2C EGR2 TESC FOS PROX1 ETS1 BARX2 FOSL1 NFKBIA IL1B IRF1 BCL3 TIFA*
43122	regulation of I-kappaB kinase/NF-kappaB cascade	6.22E-04	7.47E-03	7/202 3.4%	127/17784 0.7%	*IL1B UBD TNFAIP3 TNFRSF11A IKBKE NFKBID TMEM9B*
10942	positive regulation of cell death	6.38E-04	7.60E-03	14/202 6.9%	449/17784 2.5%	*VAV3 PRKCE TNFRSF9 ETS1 DUSP6 FOSL1 ALDH1A3 IL1B UBD BCL3 SOS1 IKBKE PHLDA1 NFKBID*
30335	positive regulation of cell migration	6.52E-04	7.74E-03	7/202 3.4%	128/17784 0.7%	*CXCL10 CSF1 SERPINE1 WNT5A PROX1 ICAM1 HBEGF*
30217	T cell differentiation	6.89E-04	8.10E-03	5/202 2.4%	62/17784 0.3%	*STAT5A EGR1 IRF1 BCL3 RELB*
7249	I-kappaB kinase/NF-kappaB cascade	6.89E-04	8.10E-03	5/202 2.4%	62/17784 0.3%	*NFKBIA IRAK2 BCL3 TIFA TLR7*
51254	positive regulation of RNA metabolic process	6.91E-04	8.10E-03	15/202 7.4%	506/17784 2.8%	*STAT5A EGR1 MEF2C EGR2 TESC FOS PROX1 ETS1 BARX2 FOSL1 NFKBIA IL1B IRF1 BCL3 TIFA*
1889	liver development	7.41E-04	8.65E-03	5/202 2.4%	63/17784 0.3%	*UGT1A1 CYP1A1 AHRR PROX1 WNT4*
42093	T-helper cell differentiation	7.59E-04	8.66E-03	2/202 0.9%	4/17784 0.0%	*BCL3 RELB*

9624	response to nematode	7.59E-04	8.66E-03	2/202 0.9%	4/17784 0.0%	*CYP1A1 AHRR*
22028	tangential migration from the subventricular zone to the olfactory bulb	7.59E-04	8.66E-03	2/202 0.9%	4/17784 0.0%	*SLIT1 ROBO1*
2294	CD4-positive, alpha-beta T cell differentiation involved in immune response	7.59E-04	8.66E-03	2/202 0.9%	4/17784 0.0%	*BCL3 RELB*
60763	mammary duct terminal end bud growth	7.59E-04	8.66E-03	2/202 0.9%	4/17784 0.0%	*CSF1 ROBO1*
60341	regulation of cellular localization	7.75E-04	8.78E-03	11/202 5.4%	306/17784 1.7%	*NFKBIA OXTR NOS2 IL1B LGI3 WNT5A BCL3 SAA1 SAA2 TLR7 CRYAB*
51179	localization	7.76E-04	8.78E-03	52/202 25.7%	2985/17784 16.7%	*OXTR CXCL8 RASEF CXCL3 ETS1 SLC2A6 CX3CL1 ICAM1 ROBO1 NPPB RAB43 FGF9 DUOXA2 MBL2 SLC34A3 VAV3 UNC13A SLC2A10 WNT5A SLC7A5 SNPH IL1B MYH4 ATP6V0D2 SCN4B VAMP2 ABCG2 SLC45A4 ITPR1 ATP10B TNFRSF11A STX11 ARL17B APOF STX6 SLIT1 NPTX1 PACSIN1 WNT4 STAT5A KCNF1 CCL22 NDUFA5 NFKBIA CXCL10 GJB2 BCL3 SAA1 LCN2 ATP13A4 SAA2 TRIM36*
45639	positive regulation of myeloid cell differentiation	7.96E-04	8.92E-03	4/202 1.9%	37/17784 0.2%	*STAT5A CSF1 TESC ETS1*
55123	digestive system development	7.97E-04	8.92E-03	5/202 2.4%	64/17784 0.3%	*OXTR FGF9 WNT5A CYP1A1 AHRR*
61008	hepaticobiliary system development	7.97E-04	8.92E-03	5/202 2.4%	64/17784 0.3%	*UGT1A1 CYP1A1 AHRR PROX1 WNT4*

19216	regulation of lipid metabolic process	8.19E-04	9.13E-03	7/202 3.4%	133/17784 0.7%	*STAT5A VAV3 SERPINA3 UGT1A1 IL1B PROX1 WNT4*
30334	regulation of cell migration	8.32E-04	9.24E-03	9/202 4.4%	216/17784 1.2%	*CXCL10 CSF1 PLAU LAMA4 SERPINE1 WNT5A PROX1 ICAM1 HBEGF*
35467	negative regulation of signaling pathway	8.50E-04	9.41E-03	10/202 4.9%	262/17784 1.4%	*NFKBIA FGF9 IL1B WNT5A SLIT1 TNFAIP3 NFKBID DUSP6 ROBO1 WNT4*
31076	embryonic camera-type eye development	8.73E-04	9.56E-03	3/202 1.4%	17/17784 0.0%	*ALDH1A3 WNT5A PROX1*
60324	face development	8.73E-04	9.56E-03	3/202 1.4%	17/17784 0.0%	*ALDH1A3 TIPARP WNT5A*
48048	embryonic eye morphogenesis	8.73E-04	9.56E-03	3/202 1.4%	17/17784 0.0%	*ALDH1A3 WNT5A PROX1*
2573	myeloid leukocyte differentiation	8.81E-04	9.62E-03	4/202 1.9%	38/17784 0.2%	*CSF1 UBD TNFRSF11A RELB*
7346	regulation of mitotic cell cycle	8.88E-04	9.66E-03	8/202 3.9%	175/17784 0.9%	*BTC STAT5A IL1B UBD CYP1A1 TGFA AHRR PROX1*
51047	positive regulation of secretion	8.94E-04	9.68E-03	7/202 3.4%	135/17784 0.7%	*BTC OXTR IL1B WNT5A SAA1 SAA2 TNFRSF11A*
6800	oxygen and reactive oxygen species metabolic process	9.17E-04	9.86E-03	5/202 2.4%	66/17784 0.3%	*NOS2 TIFA CYP1A1 AHRR CRYAB*
7517	muscle organ development	9.17E-04	9.86E-03	9/202 4.4%	219/17784 1.2%	*RCAN1 CXCL10 MEF2C MEF2B MRAS TIPARP PROX1 CRYAB HBEGF*
2263	cell activation involved in immune response	9.74E-04	1.04E-02	4/202 1.9%	39/17784 0.2%	*BCL3 TLR7 ICAM1 RELB*

2366	leukocyte activation involved in immune response	9.74E-04	1.04E-02	4/202 1.9%	39/17784 0.2%	*BCL3 TLR7 ICAM1 RELB*
2221	pattern recognition receptor signaling pathway	1.04E-03	1.11E-02	3/202 1.4%	18/17784 0.1%	*NFKBIA IRAK2 TLR7*
10564	regulation of cell cycle process	1.06E-03	1.12E-02	7/202 3.4%	139/17784 0.7%	*BTC IL1B UBD CYP1A1 TGFA AHRR PROX1*
45833	negative regulation of lipid metabolic process	1.07E-03	1.12E-02	4/202 1.9%	40/17784 0.2%	*UGT1A1 IL1B PROX1 WNT4*
51781	positive regulation of cell division	1.07E-03	1.12E-02	4/202 1.9%	40/17784 0.2%	*BTC FGF9 IL1B TGFA*
2685	regulation of leukocyte migration	1.07E-03	1.12E-02	4/202 1.9%	40/17784 0.2%	*CXCL10 SERPINE1 WNT5A ICAM1*
48545	response to steroid hormone stimulus	1.11E-03	1.16E-02	9/202 4.4%	225/17784 1.2%	*FOSL1 OXTR UGT1A1 IL1B TAT SERPINE1 SLIT1 FOS CRYAB*
33189	response to vitamin A	1.12E-03	1.16E-02	5/202 2.4%	69/17784 0.3%	*WNT6 WNT7B WNT5A CYP1A1 AHRR*
50795	regulation of behavior	1.12E-03	1.16E-02	5/202 2.4%	69/17784 0.3%	*CXCL10 CXCL8 SERPINE1 WNT5A ROBO1*
16331	morphogenesis of embryonic epithelium	1.12E-03	1.16E-02	5/202 2.4%	69/17784 0.3%	*ALDH1A3 WNT7B WNT5A RGMA WNT4*
72001	renal system development	1.14E-03	1.18E-02	6/202 2.9%	103/17784 0.5%	*ALDH1A3 TIPARP WNT7B WNT5A PROX1 WNT4*
21510	spinal cord development	1.18E-03	1.21E-02	4/202 1.9%	41/17784 0.2%	*SLIT1 PROX1 LHX4 ROBO1*

50921	positive regulation of chemotaxis	1.18E-03	1.21E-02	4/202 1.9%	41/17784 0.2%	*CXCL10 CXCL8 SERPINE1 WNT5A*
2460	adaptive immune response based on somatic recombination of immune receptors built from immunoglobulin superfamily domains	1.20E-03	1.21E-02	5/202 2.4%	70/17784 0.3%	*BCL3 ICAM1 RELB NFKB2 MBL2*
19221	cytokine-mediated signaling pathway	1.20E-03	1.21E-02	5/202 2.4%	70/17784 0.3%	*STAT5A IRAK2 IL1B TNFRSF11A CX3CL1*
1655	urogenital system development	1.20E-03	1.21E-02	7/202 3.4%	142/17784 0.7%	*STAT5A ALDH1A3 TIPARP WNT7B WNT5A PROX1 WNT4*
44092	negative regulation of molecular function	1.21E-03	1.21E-02	12/202 5.9%	374/17784 2.1%	*NFKBIA SPRED1 IRAK2 IL1B SERPINE1 S1PR3 PROX1 SSTR2 CRYAB NFKBID DUSP6 GNAI1*
46631	alpha-beta T cell activation	1.22E-03	1.21E-02	3/202 1.4%	19/17784 0.1%	*IRF1 BCL3 RELB*
71300	cellular response to retinoic acid	1.22E-03	1.21E-02	3/202 1.4%	19/17784 0.1%	*WNT6 WNT7B WNT5A*
2285	lymphocyte activation involved in immune response	1.22E-03	1.21E-02	3/202 1.4%	19/17784 0.1%	*BCL3 ICAM1 RELB*
7090	regulation of S phase of mitotic cell cycle	1.22E-03	1.21E-02	3/202 1.4%	19/17784 0.1%	*CYP1A1 AHRR PROX1*
32370	positive regulation of lipid transport	1.22E-03	1.21E-02	3/202 1.4%	19/17784 0.1%	*NFKBIA IL1B TNFRSF11A*
8150	biological_process	1.24E-03	1.21E-02	179/202	14300/17784	*PAGE4 OXTR CD83 CSF1 SERPINE1 RASEF*

				88.6%	80.4%	*TBC1D3F IKZF3 ETS1 ACTG2 TBC1D3G ICAM1 NPPB RAB43 CAPN6 FGF9 PLAU ZC3H12A DUOXA2 CYP1B1 ZNF169 PHLDA1 ICOSLG IER3 SLC34A3 IGFBP1 MEF2C UNC13A MEF2B SNRPN TPRA1 ARL14 PRKCE WNT5A C8ORF4 GPR37L1 RNASE2 TUBA4A BTC ALDH3A1 SYPL1 ALPK3 ATP6V0D2 ABCG2 SLC45A4 NXPH3 ITPR1 TGFA DAPP1 TNFRSF11A RASGRP3 ARL17B WNT6 IRAK2 GJA10 UBD APOF NPTX1 PACSIN1 WNT4 STAT5A EGR1 KCNF1 EGR2 PSAPL1 TIPARP TNFRSF9 FOXN4 FHDC1 LSM4 NFKB2 FOSL1 ABTB2 NFKBIA RCAN1 CXCL10 GJB2 CXCL11 BCL3 MAFF CYP1A1 LCN2 LHX4 NFKBID TMEM9B SERPINA3 ROBO3 DYRK3 CXCL8 TAT TNFAIP2 TNFAIP3 ADRA1D CXCL1 CXCL3 AREG CXCL2 SLC2A6 CX3CL1 ROBO1 RND1 MMP25 DNMT3L ACOXL SPRED1 OLR1 PGM5 CCR6 CA8 IKBKE LMCD1 MBL2 DUSP4 VAV3 HS3ST3B1 FGFBP1 SERPINB2 UGT1A1 SLC2A10 PCDHGA3 TESC HUNK FOS PROX1 SSTR2 DUSP6 RGMA SLC7A5 ALDH1A3 MRAS SNPH IL1B IRF1 CDH11 AHRR TLR7 NCOA7 MYH4 SOS1 CRYAB SCN4B VAMP2 HBEGF BIRC3 LAMA4 RGS16 FAM129A GDPD1 ATP10B BARX2 GNAI1 STX11 RELB PALMD TIFA STX6 SLIT1 TDH S1PR3 CCL22 NDUFA5 NOS2 CCL20 GAD1 WNT7B LGI3 SAMD4A CABLES1 GRHL3 SGPP2 APLN KRT17 DHRS9 FABP6 SAA1 ATP13A4 SAA2 TRIM36 FSBP*
2287	alpha-beta T cell activation involved in immune response	1.26E-03	1.21E-02	2/202<br>0.9%	5/17784<br>0.0%	*BCL3 RELB*
2292	T cell differentiation involved in immune response	1.26E-03	1.21E-02	2/202<br>0.9%	5/17784<br>0.0%	*BCL3 RELB*

2293	alpha-beta T cell differentiation involved in immune response	1.26E-03	1.21E-02	2/202 0.9%	5/17784 0.0%	*BCL3 RELB*
2467	germinal center formation	1.26E-03	1.21E-02	2/202 0.9%	5/17784 0.0%	*BCL3 NFKB2*
16199	axon midline choice point recognition	1.26E-03	1.21E-02	2/202 0.9%	5/17784 0.0%	*ROBO3 ROBO1*
9967	positive regulation of signal transduction	1.26E-03	1.21E-02	10/202 4.9%	276/17784 1.5%	*CSF1 FGF9 IL1B UBD WNT5A TNFRSF11A IKBKE LMCD1 TMEM9B HBEGF*
23036	initiation of signal transduction	1.28E-03	1.22E-02	5/202 2.4%	71/17784 0.3%	*STAT5A IRAK2 IL1B TNFRSF11A CX3CL1*
23038	signal initiation by diffusible mediator	1.28E-03	1.22E-02	5/202 2.4%	71/17784 0.3%	*STAT5A IRAK2 IL1B TNFRSF11A CX3CL1*
23049	signal initiation by protein/peptide mediator	1.28E-03	1.22E-02	5/202 2.4%	71/17784 0.3%	*STAT5A IRAK2 IL1B TNFRSF11A CX3CL1*
10648	negative regulation of cell communication	1.36E-03	1.29E-02	11/202 5.4%	328/17784 1.8%	*NFKBIA FGF9 IL1B RGS16 WNT5A SLIT1 TNFAIP3 NFKBID DUSP6 ROBO1 WNT4*
45766	positive regulation of angiogenesis	1.41E-03	1.33E-02	4/202 1.9%	43/17784 0.2%	*IL1B SERPINE1 WNT5A CX3CL1*
2699	positive regulation of immune effector process	1.41E-03	1.33E-02	4/202 1.9%	43/17784 0.2%	*STAT5A NOS2 IL1B WNT5A*
51223	regulation of protein transport	1.41E-03	1.33E-02	7/202 3.4%	146/17784 0.8%	*NFKBIA IL1B WNT5A BCL3 SAA1 SAA2 TLR7*
71299	cellular response to vitamin A	1.43E-03	1.34E-02	3/202	20/17784	*WNT6 WNT7B WNT5A*

				1.4%	0.1%	
2758	innate immune response-activating signal transduction	1.43E-03	1.34E-02	3/202 1.4%	20/17784 0.1%	*NFKBIA IRAK2 TLR7*
23056	positive regulation of signaling process	1.44E-03	1.35E-02	10/202 4.9%	281/17784 1.5%	*CSF1 FGF9 IL1B UBD WNT5A TNFRSF11A IKBKE LMCD1 TMEM9B HBEGF*
50776	regulation of immune response	1.50E-03	1.40E-02	9/202 4.4%	235/17784 1.3%	*STAT5A NFKBIA NOS2 IRAK2 IL1B WNT5A TLR7 ICAM1 MBL2*
1654	eye development	1.53E-03	1.42E-02	7/202 3.4%	148/17784 0.8%	*ALDH1A3 WNT7B WNT5A CYP1A1 AHRR PROX1 CRYAB*
19218	regulation of steroid metabolic process	1.54E-03	1.42E-02	4/202 1.9%	44/17784 0.2%	*STAT5A UGT1A1 PROX1 WNT4*
45637	regulation of myeloid cell differentiation	1.63E-03	1.50E-02	5/202 2.4%	75/17784 0.4%	*STAT5A NFKBIA CSF1 TESC ETS1*
9725	response to hormone stimulus	1.64E-03	1.50E-02	13/202 6.4%	441/17784 2.4%	*STAT5A IGFBP1 EGR1 OXTR EGR2 UGT1A1 TAT SERPINE1 FOS FOSL1 IL1B SLIT1 CRYAB*
8299	isoprenoid biosynthetic process	1.65E-03	1.50E-02	3/202 1.4%	21/17784 0.1%	*DHRS9 CYP1A1 AHRR*
2218	activation of innate immune response	1.65E-03	1.50E-02	3/202 1.4%	21/17784 0.1%	*NFKBIA IRAK2 TLR7*
10469	regulation of receptor activity	1.65E-03	1.50E-02	3/202 1.4%	21/17784 0.1%	*PLAU SERPINE1 TGFA*
32677	regulation of interleukin-8 production	1.65E-03	1.50E-02	3/202 1.4%	21/17784 0.1%	*SERPINE1 BCL3 TLR7*

51046	regulation of secretion	1.83E-03	1.65E-02	9/202 4.4%	242/17784 1.3%	*BTC OXTR NOS2 IL1B LGI3 WNT5A SAA1 SAA2 TNFRSF11A*
7611	learning or memory	1.84E-03	1.65E-02	6/202 2.9%	113/17784 0.6%	*FOSL1 EGR1 OXTR EGR2 IL1B FOS*
70307	lens fiber cell development	1.87E-03	1.65E-02	2/202 0.9%	6/17784 0.0%	*WNT7B PROX1*
9804	coumarin metabolic process	1.87E-03	1.65E-02	2/202 0.9%	6/17784 0.0%	*CYP1A1 AHRR*
50919	negative chemotaxis	1.87E-03	1.65E-02	2/202 0.9%	6/17784 0.0%	*SLIT1 ROBO1*
71560	cellular response to transforming growth factor beta stimulus	1.87E-03	1.65E-02	2/202 0.9%	6/17784 0.0%	*WNT5A WNT4*
40037	negative regulation of fibroblast growth factor receptor signaling pathway	1.87E-03	1.65E-02	2/202 0.9%	6/17784 0.0%	*WNT5A WNT4*
16198	axon choice point recognition	1.87E-03	1.65E-02	2/202 0.9%	6/17784 0.0%	*ROBO3 ROBO1*
70848	response to growth factor stimulus	1.90E-03	1.66E-02	3/202 1.4%	22/17784 0.1%	*WNT5A DUSP6 WNT4*
1959	regulation of cytokine-mediated signaling pathway	1.90E-03	1.66E-02	3/202 1.4%	22/17784 0.1%	*IRAK2 SLIT1 ROBO1*
19439	aromatic compound catabolic process	1.90E-03	1.66E-02	3/202 1.4%	22/17784 0.1%	*TAT CYP1A1 AHRR*

70201	regulation of establishment of protein localization	1.91E-03	1.66E-02	7/202 3.4%	154/17784 0.8%	*NFKBIA IL1B WNT5A BCL3 SAA1 SAA2 TLR7*
10817	regulation of hormone levels	1.91E-03	1.66E-02	7/202 3.4%	154/17784 0.8%	*ALDH1A3 DHRS9 UGT1A1 CYP1A1 CYP1B1 AHRR WNT4*
48520	positive regulation of behavior	1.97E-03	1.70E-02	4/202 1.9%	47/17784 0.2%	*CXCL10 CXCL8 SERPINE1 WNT5A*
22612	gland morphogenesis	2.05E-03	1.77E-02	5/202 2.4%	79/17784 0.4%	*STAT5A CSF1 WNT5A ROBO1 WNT4*
71295	cellular response to vitamin	2.16E-03	1.85E-02	3/202 1.4%	23/17784 0.1%	*WNT6 WNT7B WNT5A*
2690	positive regulation of leukocyte chemotaxis	2.16E-03	1.85E-02	3/202 1.4%	23/17784 0.1%	*CXCL10 SERPINE1 WNT5A*
60322	head development	2.16E-03	1.85E-02	3/202 1.4%	23/17784 0.1%	*ALDH1A3 TIPARP WNT5A*
32642	regulation of chemokine production	2.16E-03	1.85E-02	3/202 1.4%	23/17784 0.1%	*IL1B WNT5A TLR7*
70482	response to oxygen levels	2.29E-03	1.94E-02	7/202 3.4%	159/17784 0.8%	*OXTR NOS2 PLAU CYP1A1 ITPR1 AHRR CRYAB*
45089	positive regulation of innate immune response	2.30E-03	1.94E-02	4/202 1.9%	49/17784 0.2%	*STAT5A NFKBIA IRAK2 TLR7*
71216	cellular response to biotic stimulus	2.30E-03	1.94E-02	4/202 1.9%	49/17784 0.2%	*NFKBIA IRAK2 SERPINE1 WNT5A*
51091	positive regulation of transcription factor activity	2.42E-03	2.03E-02	5/202 2.4%	82/17784 0.4%	*IRAK2 IL1B WNT5A TNFRSF11A ICAM1*

6766	vitamin metabolic process	2.42E-03	2.03E-02	5/202 2.4%	82/17784 0.4%	*ALDH1A3 DHRS9 UGT1A1 CYP1A1 AHRR*
90047	positive regulation of transcription regulator activity	2.42E-03	2.03E-02	5/202 2.4%	82/17784 0.4%	*IRAK2 IL1B WNT5A TNFRSF11A ICAM1*
45840	positive regulation of mitosis	2.45E-03	2.03E-02	3/202 1.4%	24/17784 0.1%	*BTC IL1B TGFA*
42345	regulation of NF-kappaB import into nucleus	2.45E-03	2.03E-02	3/202 1.4%	24/17784 0.1%	*NFKBIA BCL3 TLR7*
1910	regulation of leukocyte mediated cytotoxicity	2.45E-03	2.03E-02	3/202 1.4%	24/17784 0.1%	*STAT5A NOS2 ICAM1*
51785	positive regulation of nuclear division	2.45E-03	2.03E-02	3/202 1.4%	24/17784 0.1%	*BTC IL1B TGFA*
32570	response to progesterone stimulus	2.45E-03	2.03E-02	3/202 1.4%	24/17784 0.1%	*FOSL1 OXTR FOS*
30900	forebrain development	2.46E-03	2.03E-02	7/202 3.4%	161/17784 0.9%	*ALDH1A3 OXTR WNT7B WNT5A SLIT1 ROBO1 WNT4*
2009	morphogenesis of an epithelium	2.49E-03	2.05E-02	8/202 3.9%	206/17784 1.1%	*STAT5A ALDH1A3 CSF1 WNT7B WNT5A ROBO1 RGMA WNT4*
46661	male sex differentiation	2.55E-03	2.09E-02	5/202 2.4%	83/17784 0.4%	*STAT5A GJB2 FGF9 WNT5A WNT4*
40007	growth	2.57E-03	2.10E-02	8/202 3.9%	207/17784 1.1%	*IGFBP1 CSF1 PLAU WNT7B SERPINE1 WNT5A SLIT1 ROBO1*
71559	response to transforming	2.60E-03	2.11E-02	2/202	7/17784	*WNT5A WNT4*

	growth factor beta stimulus			0.9%	0.0%	
43367	CD4-positive, alpha beta T cell differentiation	2.60E-03	2.11E-02	2/202 0.9%	7/17784 0.0%	*BCL3 RELB*
10894	negative regulation of steroid biosynthetic process	2.60E-03	2.11E-02	2/202 0.9%	7/17784 0.0%	*PROX1 WNT4*
45597	positive regulation of cell differentiation	2.61E-03	2.11E-02	9/202 4.4%	255/17784 1.4%	*BTC STAT5A CD83 CSF1 TESC WNT7B ETS1 ROBO1 WNT4*
40008	regulation of growth	2.69E-03	2.17E-02	11/202 5.4%	358/17784 2.0%	*STAT5A ABTB2 NPPB IGFBP1 CSF1 FGF9 SLIT1 PROX1 CRYAB HBEGF MBL2*
8202	steroid metabolic process	2.73E-03	2.20E-02	8/202 3.9%	209/17784 1.1%	*DHRS9 UGT1A1 FABP6 APOF CYP1A1 CYP1B1 AHRR WNT4*
33261	regulation of S phase	2.76E-03	2.20E-02	3/202 1.4%	25/17784 0.1%	*CYP1A1 AHRR PROX1*
17144	drug metabolic process	2.76E-03	2.20E-02	3/202 1.4%	25/17784 0.1%	*UGT1A1 CYP1A1 AHRR*
45927	positive regulation of growth	2.83E-03	2.25E-02	5/202 2.4%	85/17784 0.4%	*STAT5A IGFBP1 CSF1 PROX1 HBEGF*
302	response to reactive oxygen species	2.98E-03	2.35E-02	5/202 2.4%	86/17784 0.4%	*FOSL1 SERPINE1 OLR1 FOS CRYAB*
30278	regulation of ossification	2.98E-03	2.35E-02	5/202 2.4%	86/17784 0.4%	*EGR2 CSF1 WNT7B WNT5A WNT4*
51222	positive regulation of protein transport	2.98E-03	2.35E-02	5/202 2.4%	86/17784 0.4%	*IL1B WNT5A SAA1 SAA2 TLR7*

32088	negative regulation of NF-kappaB transcription factor activity	3.10E-03	2.44E-02	3/202 1.4%	26/17784 0.1%	*NFKBIA IRAK2 NFKBID*
44057	regulation of system process	3.16E-03	2.48E-02	10/202 4.9%	313/17784 1.7%	*BTC NPPB EGR1 OXTR EGR2 NOS2 IL1B SSTR2 LMCD1 HBEGF*
42592	homeostatic process	3.26E-03	2.55E-02	18/202 8.9%	775/17784 4.3%	*SLC34A3 STAT5A SERPINA3 OXTR EGR2 DYRK3 CSF1 WNT7B SERPINE1 ITPR1 TNFRSF11A NPPB IL1B SAA1 LCN2 SAA2 S1PR3 CCR6*
45080	positive regulation of chemokine biosynthetic process	3.44E-03	2.61E-02	2/202 0.9%	8/17784 0.0%	*IL1B WNT5A*
42359	vitamin D metabolic process	3.44E-03	2.61E-02	2/202 0.9%	8/17784 0.0%	*CYP1A1 AHRR*
9629	response to gravity	3.44E-03	2.61E-02	2/202 0.9%	8/17784 0.0%	*FOSL1 FOS*
46543	development of secondary female sexual characteristics	3.44E-03	2.61E-02	2/202 0.9%	8/17784 0.0%	*STAT5A WNT5A*
32303	regulation of icosanoid secretion	3.44E-03	2.61E-02	2/202 0.9%	8/17784 0.0%	*IL1B TNFRSF11A*
32305	positive regulation of icosanoid secretion	3.44E-03	2.61E-02	2/202 0.9%	8/17784 0.0%	*IL1B TNFRSF11A*
32306	regulation of prostaglandin secretion	3.44E-03	2.61E-02	2/202 0.9%	8/17784 0.0%	*IL1B TNFRSF11A*
32308	positive regulation of prostaglandin secretion	3.44E-03	2.61E-02	2/202 0.9%	8/17784 0.0%	*IL1B TNFRSF11A*

48853	forebrain morphogenesis	3.44E-03	2.61E-02	2/202 0.9%	8/17784 0.0%	*WNT5A SLIT1*
32652	regulation of interleukin-1 production	3.45E-03	2.61E-02	3/202 1.4%	27/17784 0.1%	*SAA1 SAA2 S1PR3*
2526	acute inflammatory response	3.62E-03	2.73E-02	5/202 2.4%	90/17784 0.5%	*SERPINA3 TIFA SAA1 SAA2 MBL2*
32880	regulation of protein localization	3.67E-03	2.75E-02	7/202 3.4%	173/17784 0.9%	*NFKBIA IL1B WNT5A BCL3 SAA1 SAA2 TLR7*
31341	regulation of cell killing	3.84E-03	2.87E-02	3/202 1.4%	28/17784 0.1%	*STAT5A NOS2 ICAM1*
2688	regulation of leukocyte chemotaxis	3.84E-03	2.87E-02	3/202 1.4%	28/17784 0.1%	*CXCL10 SERPINE1 WNT5A*
51130	positive regulation of cellular component organization	3.93E-03	2.93E-02	8/202 3.9%	222/17784 1.2%	*BTC OXTR IL1B SERPINE1 TGFA PROX1 ROBO1 MBL2*
10740	positive regulation of intracellular protein kinase cascade	3.93E-03	2.93E-02	8/202 3.9%	222/17784 1.2%	*FGF9 IL1B UBD WNT5A TNFRSF11A IKBKE TMEM9B HBEGF*
7411	axon guidance	3.98E-03	2.94E-02	5/202 2.4%	92/17784 0.5%	*ROBO3 EGR2 SLIT1 LHX4 ROBO1*
43433	negative regulation of transcription factor activity	3.99E-03	2.94E-02	4/202 1.9%	57/17784 0.3%	*NFKBIA IRAK2 PROX1 NFKBID*
32102	negative regulation of response to external stimulus	3.99E-03	2.94E-02	4/202 1.9%	57/17784 0.3%	*SERPINE1 SAA1 SAA2 ROBO1*
90048	negative regulation of	3.99E-03	2.94E-02	4/202	57/17784	*NFKBIA IRAK2 PROX1 NFKBID*

	transcription regulator activity			1.9%	0.3%	
43388	positive regulation of DNA binding	4.17E-03	3.06E-02	5/202 2.4%	93/17784 0.5%	*IRAK2 IL1B WNT5A TNFRSF11A ICAM1*
9110	vitamin biosynthetic process	4.24E-03	3.06E-02	3/202 1.4%	29/17784 0.1%	*DHRS9 CYP1A1 AHRR*
21983	pituitary gland development	4.24E-03	3.06E-02	3/202 1.4%	29/17784 0.1%	*ALDH1A3 WNT5A WNT4*
50715	positive regulation of cytokine secretion	4.24E-03	3.06E-02	3/202 1.4%	29/17784 0.1%	*WNT5A SAA1 SAA2*
46677	response to antibiotic	4.24E-03	3.06E-02	3/202 1.4%	29/17784 0.1%	*CYP1A1 AHRR ETS1*
31670	cellular response to nutrient	4.24E-03	3.06E-02	3/202 1.4%	29/17784 0.1%	*WNT6 WNT7B WNT5A*
7595	lactation	4.24E-03	3.06E-02	3/202 1.4%	29/17784 0.1%	*STAT5A OXTR APLN*
48729	tissue morphogenesis	4.29E-03	3.07E-02	9/202 4.4%	275/17784 1.5%	*STAT5A ALDH1A3 CSF1 WNT7B WNT5A PROX1 ROBO1 RGMA WNT4*
30005	cellular di-, tri-valent inorganic cation homeostasis	4.38E-03	3.07E-02	8/202 3.9%	226/17784 1.2%	*OXTR IL1B SAA1 ITPR1 LCN2 SAA2 S1PR3 CCR6*
45136	development of secondary sexual characteristics	4.39E-03	3.07E-02	2/202 0.9%	9/17784 0.0%	*STAT5A WNT5A*
45414	regulation of interleukin-8 biosynthetic process	4.39E-03	3.07E-02	2/202 0.9%	9/17784 0.0%	*BCL3 TLR7*

17085	response to insecticide	4.39E-03	3.07E-02	2/202 0.9%	9/17784 0.0%	*CYP1A1 AHRR*
50665	hydrogen peroxide biosynthetic process	4.39E-03	3.07E-02	2/202 0.9%	9/17784 0.0%	*CYP1A1 AHRR*
30316	osteoclast differentiation	4.39E-03	3.07E-02	2/202 0.9%	9/17784 0.0%	*CSF1 TNFRSF11A*
30431	sleep	4.39E-03	3.07E-02	2/202 0.9%	9/17784 0.0%	*OXTR FOS*
43011	myeloid dendritic cell differentiation	4.39E-03	3.07E-02	2/202 0.9%	9/17784 0.0%	*UBD RELB*
31620	regulation of fever	4.39E-03	3.07E-02	2/202 0.9%	9/17784 0.0%	*IL1B TNFRSF11A*
31622	positive regulation of fever	4.39E-03	3.07E-02	2/202 0.9%	9/17784 0.0%	*IL1B TNFRSF11A*
51783	regulation of nuclear division	4.52E-03	3.15E-02	4/202 1.9%	59/17784 0.3%	*BTC IL1B UBD TGFA*
7088	regulation of mitosis	4.52E-03	3.15E-02	4/202 1.9%	59/17784 0.3%	*BTC IL1B UBD TGFA*
30098	lymphocyte differentiation	4.56E-03	3.17E-02	5/202 2.4%	95/17784 0.5%	*STAT5A EGR1 IRF1 BCL3 RELB*
30099	myeloid cell differentiation	4.56E-03	3.17E-02	5/202 2.4%	95/17784 0.5%	*DYRK3 CSF1 UBD TNFRSF11A RELB*
45995	regulation of embryonic development	4.68E-03	3.23E-02	3/202 1.4%	30/17784 0.1%	*IL1B LAMA4 WNT5A*

32663	regulation of interleukin-2 production	4.68E-03	3.23E-02	3/202 1.4%	30/17784 0.1%	*STAT5A CD83 IL1B*
7417	central nervous system development	4.75E-03	3.27E-02	12/202 5.9%	442/17784 2.4%	*RCAN1 ALDH1A3 OXTR EGR2 WNT7B WNT5A SLIT1 PROX1 NPTX1 LHX4 ROBO1 WNT4*
31668	cellular response to extracellular stimulus	4.77E-03	3.28E-02	5/202 2.4%	96/17784 0.5%	*FOSL1 WNT6 WNT7B WNT5A FOS*
43193	positive regulation of gene-specific transcription	4.79E-03	3.28E-02	6/202 2.9%	137/17784 0.7%	*NFKBIA MEF2C TESC IL1B PROX1 ETS1*
45088	regulation of innate immune response	4.80E-03	3.28E-02	4/202 1.9%	60/17784 0.3%	*STAT5A NFKBIA IRAK2 TLR7*
71496	cellular response to external stimulus	4.99E-03	3.39E-02	5/202 2.4%	97/17784 0.5%	*FOSL1 WNT6 WNT7B WNT5A FOS*
32386	regulation of intracellular transport	4.99E-03	3.39E-02	5/202 2.4%	97/17784 0.5%	*NFKBIA IL1B BCL3 TLR7 CRYAB*
19932	second-messenger-mediated signaling	4.99E-03	3.39E-02	8/202 3.9%	231/17784 1.2%	*RCAN1 CXCL8 NOS2 ADRA1D S1PR3 CA8 SSTR2 GNAI1*
6979	response to oxidative stress	5.13E-03	3.46E-02	7/202 3.4%	184/17784 1.0%	*FOSL1 TAT SERPINE1 OLR1 FOS CRYAB MBL2*
16525	negative regulation of angiogenesis	5.13E-03	3.46E-02	3/202 1.4%	31/17784 0.1%	*NPPB CXCL10 SERPINE1*
42246	tissue regeneration	5.13E-03	3.46E-02	3/202 1.4%	31/17784 0.1%	*IGFBP1 PLAU SERPINE1*
51726	regulation of cell cycle	5.19E-03	3.49E-02	12/202 5.9%	447/17784 2.5%	*BTC STAT5A FOSL1 CXCL8 IL1B UBD CYP1A1 TGFA AHRR CABLES1 PROX1 ETS1*

6874	cellular calcium ion homeostasis	5.28E-03	3.52E-02	7/202 3.4%	185/17784 1.0%	*OXTR IL1B SAA1 ITPR1 SAA2 S1PR3 CCR6*
60429	epithelium development	5.29E-03	3.52E-02	10/202 4.9%	337/17784 1.8%	*STAT5A ALDH1A3 DHRS9 CSF1 WNT7B WNT5A PROX1 ROBO1 RGMA WNT4*
46649	lymphocyte activation	5.43E-03	3.52E-02	7/202 3.4%	186/17784 1.0%	*STAT5A EGR1 IRF1 BCL3 ICOSLG ICAM1 RELB*
8210	estrogen metabolic process	5.44E-03	3.52E-02	2/202 0.9%	10/17784 0.0%	*UGT1A1 CYP1B1*
70306	lens fiber cell differentiation	5.44E-03	3.52E-02	2/202 0.9%	10/17784 0.0%	*WNT7B PROX1*
42088	T-helper 1 type immune response	5.44E-03	3.52E-02	2/202 0.9%	10/17784 0.0%	*BCL3 RELB*
42104	positive regulation of activated T cell proliferation	5.44E-03	3.52E-02	2/202 0.9%	10/17784 0.0%	*STAT5A ICOSLG*
46685	response to arsenic	5.44E-03	3.52E-02	2/202 0.9%	10/17784 0.0%	*CYP1A1 AHRR*
1773	myeloid dendritic cell activation	5.44E-03	3.52E-02	2/202 0.9%	10/17784 0.0%	*UBD RELB*
6105	succinate metabolic process	5.44E-03	3.52E-02	2/202 0.9%	10/17784 0.0%	*STAT5A GAD1*
43124	negative regulation of I-kappaB kinase/NF-kappaB cascade	5.44E-03	3.52E-02	2/202 0.9%	10/17784 0.0%	*TNFAIP3 NFKBID*
43584	nose development	5.44E-03	3.52E-02	2/202	10/17784	*ALDH1A3 PROX1*

				0.9%	0.0%	
31650	regulation of heat generation	5.44E-03	3.52E-02	2/202 0.9%	10/17784 0.0%	*IL1B TNFRSF11A*
31652	positive regulation of heat generation	5.44E-03	3.52E-02	2/202 0.9%	10/17784 0.0%	*IL1B TNFRSF11A*
31663	lipopolysaccharide-mediated signaling pathway	5.44E-03	3.52E-02	2/202 0.9%	10/17784 0.0%	*NFKBIA IRAK2*
48745	smooth muscle tissue development	5.44E-03	3.52E-02	2/202 0.9%	10/17784 0.0%	*TIPARP PROX1*
32757	positive regulation of interleukin-8 production	5.44E-03	3.52E-02	2/202 0.9%	10/17784 0.0%	*SERPINE1 TLR7*
45727	positive regulation of translation	5.62E-03	3.63E-02	3/202 1.4%	32/17784 0.1%	*BCL3 FAM129A SAMD4A*
1822	kidney development	5.67E-03	3.65E-02	5/202 2.4%	100/17784 0.5%	*ALDH1A3 TIPARP WNT7B PROX1 WNT4*
9615	response to virus	5.89E-03	3.78E-02	6/202 2.9%	143/17784 0.8%	*FOSL1 CCL22 BCL3 CYP1A1 AHRR TLR7*
51093	negative regulation of developmental process	5.90E-03	3.78E-02	9/202 4.4%	289/17784 1.6%	*STAT5A NPPB NFKBIA CXCL10 SERPINE1 WNT5A SLIT1 NFKBID WNT4*
9887	organ morphogenesis	5.98E-03	3.82E-02	15/202 7.4%	634/17784 3.5%	*STAT5A CSF1 TIPARP WNT7B WNT5A PROX1 BARX2 ROBO1 RGMA WNT6 ALDH1A3 FGF9 SLIT1 LHX4 WNT4*
7399	nervous system development	6.15E-03	3.92E-02	23/202 11.3%	1154/17784 6.4%	*ROBO3 OXTR MEF2C EGR2 WNT7B WNT5A CABLES1 FOXN4 CXCL1 FOS PROX1 ROBO1*

						RGMA RND1 SLC7A5 WNT6 RCAN1 ALDH1A3 GJA10 SLIT1 NPTX1 LHX4 WNT4
904	cell morphogenesis involved in differentiation	6.24E-03	3.97E-02	8/202 3.9%	240/17784 1.3%	*ROBO3 EGR2 WNT5A SLIT1 PROX1 LHX4 ROBO1 WNT4*
7568	aging	6.29E-03	3.99E-02	6/202 2.9%	145/17784 0.8%	*TPRA1 NOS2 IL1B SERPINE1 FOS CRYAB*
32946	positive regulation of mononuclear cell proliferation	6.37E-03	4.02E-02	4/202 1.9%	65/17784 0.3%	*STAT5A CSF1 IL1B ICOSLG*
43392	negative regulation of DNA binding	6.37E-03	4.02E-02	4/202 1.9%	65/17784 0.3%	*NFKBIA IRAK2 PROX1 NFKBID*
55066	di-, tri-valent inorganic cation homeostasis	6.40E-03	4.03E-02	8/202 3.9%	241/17784 1.3%	*OXTR IL1B SAA1 ITPR1 LCN2 SAA2 S1PR3 CCR6*
19222	regulation of metabolic process	6.44E-03	4.03E-02	60/202 29.7%	3918/17784 22.0%	*SERPINA3 CSF1 SERPINE1 TBC1D3F IKZF3 AREG ETS1 TBC1D3G ICAM1 DNMT3L SPRED1 ZNF169 LMCD1 VAV3 MEF2C MEF2B UGT1A1 PRKCE TESC WNT5A FOS PROX1 SSTR2 DUSP6 IL1B IRF1 AHRR TLR7 NCOA7 CRYAB HBEGF FAM129A TGFA TNFRSF11A BARX2 GNAI1 RELB RASGRP3 WNT6 IRAK2 TIFA SLIT1 S1PR3 WNT4 STAT5A EGR1 EGR2 NOS2 SAMD4A GRHL3 FOXN4 NFKB2 FOSL1 NFKBIA RCAN1 BCL3 MAFF FSBP LHX4 NFKBID*
55074	calcium ion homeostasis	6.44E-03	4.03E-02	7/202 3.4%	192/17784 1.0%	*OXTR IL1B SAA1 ITPR1 SAA2 S1PR3 CCR6*
45137	development of primary sexual characteristics	6.50E-03	4.04E-02	6/202 2.9%	146/17784 0.8%	*STAT5A GJB2 FGF9 WNT5A SLIT1 WNT4*

50778	positive regulation of immune response	6.50E-03	4.04E-02	6/202 2.9%	146/17784 0.8%	*STAT5A NFKBIA IRAK2 IL1B TLR7 MBL2*
45073	regulation of chemokine biosynthetic process	6.60E-03	4.04E-02	2/202 0.9%	11/17784 0.0%	*IL1B WNT5A*
32892	positive regulation of organic acid transport	6.60E-03	4.04E-02	2/202 0.9%	11/17784 0.0%	*IL1B TNFRSF11A*
42481	regulation of odontogenesis	6.60E-03	4.04E-02	2/202 0.9%	11/17784 0.0%	*WNT6 CSF1*
50829	defense response to Gram-negative bacterium	6.60E-03	4.04E-02	2/202 0.9%	11/17784 0.0%	*NOS2 SERPINE1*
71363	cellular response to growth factor stimulus	6.60E-03	4.04E-02	2/202 0.9%	11/17784 0.0%	*WNT5A WNT4*
55093	response to hyperoxia	6.60E-03	4.04E-02	2/202 0.9%	11/17784 0.0%	*CYP1A1 AHRR*
1960	negative regulation of cytokine-mediated signaling pathway	6.60E-03	4.04E-02	2/202 0.9%	11/17784 0.0%	*SLIT1 ROBO1*
32094	response to food	6.60E-03	4.04E-02	2/202 0.9%	11/17784 0.0%	*CYP1A1 AHRR*
71466	cellular response to xenobiotic stimulus	6.67E-03	4.07E-02	3/202 1.4%	34/17784 0.1%	*UGT1A1 CYP1A1 AHRR*
6805	xenobiotic metabolic process	6.67E-03	4.07E-02	3/202 1.4%	34/17784 0.1%	*UGT1A1 CYP1A1 AHRR*
70665	positive regulation of	6.73E-03	4.10E-02	4/202	66/17784	*STAT5A CSF1 IL1B ICOSLG*

	leukocyte proliferation			1.9%	0.3%	
2697	regulation of immune effector process	6.95E-03	4.22E-02	5/202<br>2.4%	105/17784<br>0.5%	*STAT5A NOS2 IL1B WNT5A ICAM1*
50801	ion homeostasis	6.98E-03	4.23E-02	11/202<br>5.4%	407/17784<br>2.2%	*SLC34A3 NPPB OXTR EGR2 IL1B SAA1 ITPR1 LCN2 SAA2 S1PR3 CCR6*
35295	tube development	7.01E-03	4.24E-02	9/202<br>4.4%	297/17784<br>1.6%	*NPPB CSF1 FGF9 WNT7B WNT5A PROX1 ROBO1 RGMA WNT4*
6875	cellular metal ion homeostasis	7.18E-03	4.33E-02	7/202<br>3.4%	196/17784<br>1.1%	*OXTR IL1B SAA1 ITPR1 SAA2 S1PR3 CCR6*
7423	sensory organ development	7.38E-03	4.42E-02	8/202<br>3.9%	247/17784<br>1.3%	*ALDH1A3 FGF9 WNT7B WNT5A CYP1A1 AHRR PROX1 CRYAB*
1666	response to hypoxia	7.39E-03	4.42E-02	6/202<br>2.9%	150/17784<br>0.8%	*NOS2 PLAU CYP1A1 ITPR1 AHRR CRYAB*
50790	regulation of catalytic activity	7.56E-03	4.52E-02	19/202<br>9.4%	907/17784<br>5.1%	*VAV3 CSF1 SERPINE1 WNT5A TGFA TBC1D3F PROX1 TNFRSF11A SSTR2 DUSP6 GNAI1 TBC1D3G ROBO1 RASGRP3 RCAN1 SPRED1 IL1B S1PR3 CRYAB*
48878	chemical homeostasis	7.70E-03	4.59E-02	13/202<br>6.4%	530/17784<br>2.9%	*SLC34A3 OXTR EGR2 WNT7B SERPINE1 ITPR1 NPPB IL1B SAA1 LCN2 SAA2 S1PR3 CCR6*
7420	brain development	7.78E-03	4.63E-02	9/202<br>4.4%	302/17784<br>1.6%	*ALDH1A3 OXTR EGR2 WNT7B WNT5A SLIT1 PROX1 ROBO1 WNT4*
45885	positive regulation of survival gene product expression	7.86E-03	4.65E-02	2/202<br>0.9%	12/17784<br>0.0%	*STAT5A MEF2C*
6906	vesicle fusion	7.86E-03	4.65E-02	2/202	12/17784	*VAV3 STX6*

				0.9%	0.0%	
43405	regulation of MAP kinase activity	7.86E-03	4.65E-02	6/202 2.9%	152/17784 0.8%	*SPRED1 IL1B WNT5A TGFA TNFRSF11A DUSP6*
51099	positive regulation of binding	8.11E-03	4.79E-02	5/202 2.4%	109/17784 0.6%	*IRAK2 IL1B WNT5A TNFRSF11A ICAM1*
30003	cellular cation homeostasis	8.28E-03	4.88E-02	8/202 3.9%	252/17784 1.4%	*OXTR IL1B SAA1 ITPR1 LCN2 SAA2 S1PR3 CCR6*
50707	regulation of cytokine secretion	8.45E-03	4.97E-02	3/202 1.4%	37/17784 0.2%	*WNT5A SAA1 SAA2*
8210	estrogen metabolic process	5.44E-03	3.52E-02	2/202 0.9%	10/17784 0.0%	*UGT1A1 CYP1B1*
70306	lens fiber cell differentiation	5.44E-03	3.52E-02	2/202 0.9%	10/17784 0.0%	*WNT7B PROX1*
42088	T-helper 1 type immune response	5.44E-03	3.52E-02	2/202 0.9%	10/17784 0.0%	*BCL3 RELB*
42104	positive regulation of activated T cell proliferation	5.44E-03	3.52E-02	2/202 0.9%	10/17784 0.0%	*STAT5A ICOSLG*
46685	response to arsenic	5.44E-03	3.52E-02	2/202 0.9%	10/17784 0.0%	*CYP1A1 AHRR*
1773	myeloid dendritic cell activation	5.44E-03	3.52E-02	2/202 0.9%	10/17784 0.0%	*UBD RELB*
6105	succinate metabolic process	5.44E-03	3.52E-02	2/202 0.9%	10/17784 0.0%	*STAT5A GAD1*

43124	negative regulation of I-kappaB kinase/NF-kappaB cascade	5.44E-03	3.52E-02	2/202 0.9%	10/17784 0.0%	*TNFAIP3 NFKBID*
43584	nose development	5.44E-03	3.52E-02	2/202 0.9%	10/17784 0.0%	*ALDH1A3 PROX1*
31650	regulation of heat generation	5.44E-03	3.52E-02	2/202 0.9%	10/17784 0.0%	*IL1B TNFRSF11A*
31652	positive regulation of heat generation	5.44E-03	3.52E-02	2/202 0.9%	10/17784 0.0%	*IL1B TNFRSF11A*
31663	lipopolysaccharide-mediated signaling pathway	5.44E-03	3.52E-02	2/202 0.9%	10/17784 0.0%	*NFKBIA IRAK2*
48745	smooth muscle tissue development	5.44E-03	3.52E-02	2/202 0.9%	10/17784 0.0%	*TIPARP PROX1*
32757	positive regulation of interleukin-8 production	5.44E-03	3.52E-02	2/202 0.9%	10/17784 0.0%	*SERPINE1 TLR7*
45727	positive regulation of translation	5.62E-03	3.63E-02	3/202 1.4%	32/17784 0.1%	*BCL3 FAM129A SAMD4A*
1822	kidney development	5.67E-03	3.65E-02	5/202 2.4%	100/17784 0.5%	*ALDH1A3 TIPARP WNT7B PROX1 WNT4*
9615	response to virus	5.89E-03	3.78E-02	6/202 2.9%	143/17784 0.8%	*FOSL1 CCL22 BCL3 CYP1A1 AHRR TLR7*
51093	negative regulation of developmental process	5.90E-03	3.78E-02	9/202 4.4%	289/17784 1.6%	*STAT5A NPPB NFKBIA CXCL10 SERPINE1 WNT5A SLIT1 NFKBID WNT4*
9887	organ morphogenesis	5.98E-03	3.82E-02	15/202	634/17784	*STAT5A CSF1 TIPARP WNT7B WNT5A PROX1*

				7.4%	3.5%	*BARX2 ROBO1 RGMA WNT6 ALDH1A3 FGF9 SLIT1 LHX4 WNT4*
7399	nervous system development	6.15E-03	3.92E-02	23/202 11.3%	1154/17784 6.4%	*ROBO3 OXTR MEF2C EGR2 WNT7B WNT5A CABLES1 FOXN4 CXCL1 FOS PROX1 ROBO1 RGMA RND1 SLC7A5 WNT6 RCAN1 ALDH1A3 GJA10 SLIT1 NPTX1 LHX4 WNT4*
904	cell morphogenesis involved in differentiation	6.24E-03	3.97E-02	8/202 3.9%	240/17784 1.3%	*ROBO3 EGR2 WNT5A SLIT1 PROX1 LHX4 ROBO1 WNT4*
7568	aging	6.29E-03	3.99E-02	6/202 2.9%	145/17784 0.8%	*TPRA1 NOS2 IL1B SERPINE1 FOS CRYAB*
32946	positive regulation of mononuclear cell proliferation	6.37E-03	4.02E-02	4/202 1.9%	65/17784 0.3%	*STAT5A CSF1 IL1B ICOSLG*
43392	negative regulation of DNA binding	6.37E-03	4.02E-02	4/202 1.9%	65/17784 0.3%	*NFKBIA IRAK2 PROX1 NFKBID*
55066	di-, tri-valent inorganic cation homeostasis	6.40E-03	4.03E-02	8/202 3.9%	241/17784 1.3%	*OXTR IL1B SAA1 ITPR1 LCN2 SAA2 S1PR3 CCR6*
19222	regulation of metabolic process	6.44E-03	4.03E-02	60/202 29.7%	3918/17784 22.0%	*SERPINA3 CSF1 SERPINE1 TBC1D3F IKZF3 AREG ETS1 TBC1D3G ICAM1 DNMT3L SPRED1 ZNF169 LMCD1 VAV3 MEF2C MEF2B UGT1A1 PRKCE TESC WNT5A FOS PROX1 SSTR2 DUSP6 IL1B IRF1 AHRR TLR7 NCOA7 CRYAB HBEGF FAM129A TGFA TNFRSF11A BARX2 GNAI1 RELB RASGRP3 WNT6 IRAK2 TIFA SLIT1 S1PR3 WNT4 STAT5A EGR1 EGR2 NOS2 SAMD4A GRHL3 FOXN4 NFKB2 FOSL1 NFKBIA RCAN1 BCL3 MAFF FSBP LHX4 NFKBID*

55074	calcium ion homeostasis	6.44E-03	4.03E-02	7/202<br>3.4%	192/17784<br>1.0%	*OXTR IL1B SAA1 ITPR1 SAA2 S1PR3 CCR6*
45137	development of primary sexual characteristics	6.50E-03	4.04E-02	6/202<br>2.9%	146/17784<br>0.8%	*STAT5A GJB2 FGF9 WNT5A SLIT1 WNT4*
50778	positive regulation of immune response	6.50E-03	4.04E-02	6/202<br>2.9%	146/17784<br>0.8%	*STAT5A NFKBIA IRAK2 IL1B TLR7 MBL2*
45073	regulation of chemokine biosynthetic process	6.60E-03	4.04E-02	2/202<br>0.9%	11/17784<br>0.0%	*IL1B WNT5A*
32892	positive regulation of organic acid transport	6.60E-03	4.04E-02	2/202<br>0.9%	11/17784<br>0.0%	*IL1B TNFRSF11A*
42481	regulation of odontogenesis	6.60E-03	4.04E-02	2/202<br>0.9%	11/17784<br>0.0%	*WNT6 CSF1*
50829	defense response to Gram-negative bacterium	6.60E-03	4.04E-02	2/202<br>0.9%	11/17784<br>0.0%	*NOS2 SERPINE1*
71363	cellular response to growth factor stimulus	6.60E-03	4.04E-02	2/202<br>0.9%	11/17784<br>0.0%	*WNT5A WNT4*
55093	response to hyperoxia	6.60E-03	4.04E-02	2/202<br>0.9%	11/17784<br>0.0%	*CYP1A1 AHRR*
1960	negative regulation of cytokine-mediated signaling pathway	6.60E-03	4.04E-02	2/202<br>0.9%	11/17784<br>0.0%	*SLIT1 ROBO1*
32094	response to food	6.60E-03	4.04E-02	2/202<br>0.9%	11/17784<br>0.0%	*CYP1A1 AHRR*
71466	cellular response to	6.67E-03	4.07E-02	3/202	34/17784	*UGT1A1 CYP1A1 AHRR*

	xenobiotic stimulus			1.4%	0.1%	
6805	xenobiotic metabolic process	6.67E-03	4.07E-02	3/202<br>1.4%	34/17784<br>0.1%	*UGT1A1 CYP1A1 AHRR*
70665	positive regulation of leukocyte proliferation	6.73E-03	4.10E-02	4/202<br>1.9%	66/17784<br>0.3%	*STAT5A CSF1 IL1B ICOSLG*
2697	regulation of immune effector process	6.95E-03	4.22E-02	5/202<br>2.4%	105/17784<br>0.5%	*STAT5A NOS2 IL1B WNT5A ICAM1*
50801	ion homeostasis	6.98E-03	4.23E-02	11/202<br>5.4%	407/17784<br>2.2%	*SLC34A3 NPPB OXTR EGR2 IL1B SAA1 ITPR1 LCN2 SAA2 S1PR3 CCR6*
35295	tube development	7.01E-03	4.24E-02	9/202<br>4.4%	297/17784<br>1.6%	*NPPB CSF1 FGF9 WNT7B WNT5A PROX1 ROBO1 RGMA WNT4*
6875	cellular metal ion homeostasis	7.18E-03	4.33E-02	7/202<br>3.4%	196/17784<br>1.1%	*OXTR IL1B SAA1 ITPR1 SAA2 S1PR3 CCR6*
7423	sensory organ development	7.38E-03	4.42E-02	8/202<br>3.9%	247/17784<br>1.3%	*ALDH1A3 FGF9 WNT7B WNT5A CYP1A1 AHRR PROX1 CRYAB*
1666	response to hypoxia	7.39E-03	4.42E-02	6/202<br>2.9%	150/17784<br>0.8%	*NOS2 PLAU CYP1A1 ITPR1 AHRR CRYAB*
50790	regulation of catalytic activity	7.56E-03	4.52E-02	19/202<br>9.4%	907/17784<br>5.1%	*VAV3 CSF1 SERPINE1 WNT5A TGFA TBC1D3F PROX1 TNFRSF11A SSTR2 DUSP6 GNAI1 TBC1D3G ROBO1 RASGRP3 RCAN1 SPRED1 IL1B S1PR3 CRYAB*
48878	chemical homeostasis	7.70E-03	4.59E-02	13/202<br>6.4%	530/17784<br>2.9%	*SLC34A3 OXTR EGR2 WNT7B SERPINE1 ITPR1 NPPB IL1B SAA1 LCN2 SAA2 S1PR3 CCR6*

7420	brain development	7.78E-03	4.63E-02	9/202 4.4%	302/17784 1.6%	*ALDH1A3 OXTR EGR2 WNT7B WNT5A SLIT1 PROX1 ROBO1 WNT4*
45885	positive regulation of survival gene product expression	7.86E-03	4.65E-02	2/202 0.9%	12/17784 0.0%	*STAT5A MEF2C*
6906	vesicle fusion	7.86E-03	4.65E-02	2/202 0.9%	12/17784 0.0%	*VAV3 STX6*
43405	regulation of MAP kinase activity	7.86E-03	4.65E-02	6/202 2.9%	152/17784 0.8%	*SPRED1 IL1B WNT5A TGFA TNFRSF11A DUSP6*
51099	positive regulation of binding	8.11E-03	4.79E-02	5/202 2.4%	109/17784 0.6%	*IRAK2 IL1B WNT5A TNFRSF11A ICAM1*
30003	cellular cation homeostasis	8.28E-03	4.88E-02	8/202 3.9%	252/17784 1.4%	*OXTR IL1B SAA1 ITPR1 LCN2 SAA2 S1PR3 CCR6*
50707	regulation of cytokine secretion	8.45E-03	4.97E-02	3/202 1.4%	37/17784 0.2%	*WNT5A SAA1 SAA2*
**Molecular function**						
8009	chemokine activity	2.47E-09	2.64E-07	9/202 4.4%	47/17784 0.2%	*CXCL10 CXCL11 CCL22 CXCL8 CCL20 CXCL1 CXCL3 CXCL2 CX3CL1*
42379	chemokine receptor binding	6.35E-09	6.33E-07	9/202 4.4%	52/17784 0.2%	*CXCL10 CXCL11 CCL22 CXCL8 CCL20 CXCL1 CXCL3 CXCL2 CX3CL1*
1664	G-protein-coupled receptor binding	4.92E-08	3.77E-06	12/202 5.9%	136/17784 0.7%	*CXCL10 CXCL11 CCL22 CXCL8 CCL20 WNT5A SAA1 SAA2 CXCL1 CXCL3 CXCL2 CX3CL1*
5102	receptor binding	5.53E-08	4.13E-06	31/202 15.3%	922/17784 5.1%	*CXCL8 CSF1 LAMA4 TGFA CXCL1 CXCL3 AREG CXCL2 CX3CL1 ICAM1 NPPB SPRED1 FGF9 TIFA*

						APOF SLIT1 ICOSLG MBL2 VAV3 CCL22 CCL20 WNT5A PROX1 APLN BTC CXCL10 CXCL11 IL1B SAA1 SAA2 HBEGF
5126	cytokine receptor binding	2.13E-07	1.27E-05	13/202 6.4%	186/17784 1.0%	*CCL22 CXCL8 CSF1 CCL20 CXCL1 CXCL3 CXCL2 CX3CL1 CXCL10 CXCL11 SPRED1 IL1B TIFA*
5125	cytokine activity	4.12E-07	2.16E-05	13/202 6.4%	197/17784 1.1%	*CCL22 CXCL8 CSF1 CCL20 CXCL1 CXCL3 AREG CXCL2 CX3CL1 CXCL10 CXCL11 IL1B TIFA*
5515	protein binding	4.61E-06	1.68E-04	124/202 61.3%	8122/17784 45.6%	*OXTR CSF1 SERPINE1 IKZF3 ETS1 ACTG2 ICAM1 EDC3 NPPB FGF9 CYP1B1 PHLDA1 ICOSLG IER3 IGFBP1 MEF2C MEF2B SNRPN PRKCE WNT5A TUBA4A BTC S100A3 ATP6V0D2 ABCG2 NXPH3 ITPR1 TGFA DAPP1 TNFRSF11A RASGRP3 IRAK2 UBD APOF PACSIN1 WNT4 STAT5A EGR1 KCNF1 EGR2 TIPARP TNFRSF9 FHDC1 LSM4 NFKB2 FOSL1 ABTB2 NFKBIA RCAN1 CXCL10 GJB2 CXCL11 BCL3 CYP1A1 RNF183 NFKBID SERPINA3 DYRK3 DOCK3 CXCL8 TNFAIP3 CXCL1 CXCL3 AREG CXCL2 CX3CL1 ROBO1 RND1 DNMT3L SPRED1 OLR1 PGM5 CCR6 CA8 IKBKE LMCD1 MBL2 VAV3 FGFBP1 UGT1A1 TESC FOS PROX1 SSTR2 DUSP6 ALDH1A3 MRAS SNPH IL1B CDH11 AHRR TLR7 NCOA7 MYH4 SOS1 CRYAB VAMP2 HBEGF BIRC3 LAMA4 RGS16 BARX2 GNAI1 STX11 RELB SERTAD4 TIFA STX6 SLIT1 TRIM40 CCL22 NOS2 CCL20 GAD1 LGI3 CABLES1 GRHL3 APLN KRT17 NEURL3 SAA1 SAA2 TRIM36 FSBP*
5154	epidermal growth factor receptor binding	1.07E-05	3.18E-04	4/202 1.9%	13/17784 0.0%	*BTC VAV3 TGFA HBEGF*

16711	flavonoid 3'-monooxygenase activity	1.28E-04	2.22E-03	2/202 0.9%	2/17784 0.0%	*CYP1A1 AHRR*
70851	growth factor receptor binding	3.41E-04	4.90E-03	6/202 2.9%	82/17784 0.4%	*BTC VAV3 FGF9 IL1B TGFA HBEGF*
8083	growth factor activity	4.93E-04	6.27E-03	8/202 3.9%	160/17784 0.8%	*BTC CSF1 FGF9 IL1B TGFA CXCL1 AREG HBEGF*
70576	vitamin D 24-hydroxylase activity	7.59E-04	8.66E-03	2/202 0.9%	4/17784 0.0%	*CYP1A1 AHRR*
4704	NF-kappaB-inducing kinase activity	1.26E-03	1.21E-02	2/202 0.9%	5/17784 0.0%	*IRAK2 IKBKE*
4030	aldehyde dehydrogenase [NAD(P)+] activity	1.87E-03	1.65E-02	2/202 0.9%	6/17784 0.0%	*ALDH3A1 ALDH1A3*
70330	aromatase activity	2.76E-03	2.20E-02	3/202 1.4%	25/17784 0.1%	*CYP1A1 CYP1B1 AHRR*
16712	oxidoreductase activity, acting on paired donors, with incorporation or reduction of molecular oxygen, reduced flavin or flavoprotein as one donor, and incorporation of one atom of oxygen	3.45E-03	2.61E-02	3/202 1.4%	27/17784 0.1%	*CYP1A1 CYP1B1 AHRR*
5488	binding	3.51E-03	2.65E-02	158/202 78.2%	12362/17784 69.5%	*OXTR CSF1 GALNT18 SERPINE1 RASEF IKZF3 ETS1 ACTG2 ICAM1 EDC3 ZC3H12D NPPB RAB43 FGF9 ZC3H12A CYP1B1 ZNF169 PHLDA1 ICOSLG IER3 IGFBP1 MEF2C UNC13A MEF2B SNRPN ARL14 PRKCE WNT5A FRMD4A RNASE2 TUBA4A BTC ALPK3 S100A3 ATP6V0D2 ABCG2*

						NXPH3 ITPR1 TGFA DAPP1 TNFRSF11A RASGRP3 ARL17B IRAK2 UBD APOF NPTX1 PACSIN1 WNT4 STAT5A EGR1 APOBEC3D KCNF1 EGR2 TIPARP LHFP TNFRSF9 FOXN4 FHDC1 LSM4 NFKB2 FOSL1 ABTB2 NFKBIA RCAN1 CXCL10 GJB2 CXCL11 BCL3 MAFF CYP1A1 LCN2 LHX4 RNF183 NFKBID SERPINA3 DYRK3 DOCK3 CXCL8 TAT TNFAIP3 CXCL1 CXCL3 AREG CXCL2 CX3CL1 ROBO1 RND1 MMP25 DNMT3L ACOXL SPRED1 OLR1 PGM5 CCR6 CA8 IKBKE LMCD1 MBL2 VAV3 FGFBP1 UGT1A1 PCDHGA3 TESC HUNK FOS PROX1 SSTR2 DUSP6 SLC7A5 ALDH1A3 MRAS SNPH IL1B IRF1 CDH11 AHRR TLR7 NCOA7 MYH4 SOS1 CRYAB VAMP2 HBEGF BIRC3 LAMA4 RGS16 GDPD1 ATP10B BARX2 GNAI1 STX11 RELB SERTAD4 TIFA STX6 SLIT1 TDH S1PR3 TRIM40 CCL22 NOS2 CCL20 GAD1 LGI3 CABLES1 GRHL3 APLN DLK2 KRT17 DHRS9 FABP6 NEURL3 SAA1 ATP13A4 SAA2 TRIM36 FSBP
16709	oxidoreductase activity, acting on paired donors, with incorporation or reduction of molecular oxygen, NADH or NADPH as one donor, and incorporation of one atom of oxygen	4.24E-03	3.06E-02	3/202 1.4%	29/17784 0.1%	*NOS2 CYP1A1 AHRR*
30234	enzyme regulator activity	4.35E-03	3.07E-02	19/202 9.4%	860/17784 4.8%	*VAV3 SERPINA3 SERPINB2 DOCK3 UGT1A1 NOS2 PRKCE TESC RGS16 SERPINE1 TBC1D3F CABLES1 CXCL1 TBC1D3G RASGRP3 CXCL10 MMP25 DNMT3L SOS1*

4029	aldehyde dehydrogenase (NAD) activity	4.39E-03	3.07E-02	2/202 0.9%	9/17784 0.0%	*ALDH3A1 ALDH1A3*
16679	oxidoreductase activity, acting on diphenols and related substances as donors	5.44E-03	3.52E-02	2/202 0.9%	10/17784 0.0%	*CYP1A1 AHRR*
3700	transcription factor activity	5.87E-03	3.77E-02	20/202 9.9%	950/17784 5.3%	*STAT5A EGR1 MEF2C EGR2 MEF2B FOXN4 FOS PROX1 IKZF3 ETS1 BARX2 RELB NFKB2 FOSL1 RCAN1 IRF1 BCL3 MAFF LHX4 ZNF169*
8047	enzyme activator activity	6.33E-03	4.01E-02	10/202 4.9%	346/17784 1.9%	*VAV3 MMP25 DNMT3L PRKCE RGS16 TBC1D3F CXCL1 SOS1 TBC1D3G RASGRP3*
5243	gap junction channel activity	6.60E-03	4.04E-02	2/202 0.9%	11/17784 0.0%	*GJB2 GJA10*
**Cellular component**						
5615	extracellular space	1.91E-11	6.33E-09	33/202 16.3%	747/17784 4.2%	*CXCL8 CSF1 SERPINE1 TNFAIP2 TGFA CXCL1 CXCL3 AREG CXCL2 CX3CL1 ICAM1 NPPB FGF9 PLAU TIFA APOF SLIT1 LMCD1 WNT4 MBL2 IGFBP1 FGFBP1 SERPINB2 CCL22 CCL20 WNT5A BTC CXCL10 CXCL11 IL1B SAA1 SAA2 HBEGF*
44421	extracellular region part	1.00E-10	2.50E-08	37/202 18.3%	983/17784 5.5%	*CXCL8 CSF1 LAMA4 SERPINE1 TNFAIP2 TGFA CXCL1 CXCL3 AREG CXCL2 CX3CL1 ICAM1 NPPB WNT6 MMP25 FGF9 PLAU TIFA APOF SLIT1 LMCD1 WNT4 MBL2 IGFBP1 FGFBP1 SERPINB2 CCL22 CCL20 WNT7B WNT5A BTC CXCL10 CXCL11 IL1B SAA1 SAA2 HBEGF*
5576	extracellular region	1.91E-07	1.19E-05	49/202 24.2%	2025/17784 11.3%	*SERPINA3 OLFML2B CXCL8 CSF1 SERPINE1 TNFAIP2 CXCL1 CXCL3 AREG CXCL2 CX3CL1*

						ICAM1 NPPB MMP25 FGF9 PLAU OLR1 LMCD1 MBL2 IGFBP1 FGFBP1 SERPINB2 WNT5A RNASE2 TUBA4A BTC IL1B FAM20A HBEGF NXPH3 LAMA4 TGFA WNT6 TIFA APOF SLIT1 WNT4 CCL22 PSAPL1 CCL20 WNT7B LGI3 APLN CXCL10 CXCL11 SAA1 LCN2 SAA2 KCP
33256	I-kappaB/NF-kappaB complex	5.73E-06	1.97E-04	3/202 1.4%	4/17784 0.0%	*NFKBIA BCL3 NFKB2*
9986	cell surface	8.30E-06	2.69E-04	15/202 7.4%	340/17784 1.9%	*FGFBP1 CD83 TGFA TNFRSF11A AREG CX3CL1 ICAM1 ROBO1 RGMA MMP25 PLAU IRAK2 CRYAB HBEGF WNT4*
33257	Bcl3/NF-kappaB2 complex	1.28E-04	2.22E-03	2/202 0.9%	2/17784 0.0%	*BCL3 NFKB2*
267	cell fraction	5.51E-04	6.83E-03	25/202 12.3%	1079/17784 6.0%	*DOCK3 RGS16 SERPINE1 ITPR1 CXCL2 CLMP STX6 SLIT1 CYP1B1 OLR1 DUSP4 UGT1A1 LGI3 WNT5A SAMD4A FOS SSTR2 BTC FOSL1 DHRS9 SNPH CYP1A1 AHRR CRYAB VAMP2*
19717	synaptosome	6.32E-04	7.56E-03	6/202 2.9%	92/17784 0.5%	*FOSL1 SNPH LGI3 SAMD4A FOS VAMP2*
5626	insoluble fraction	1.23E-03	1.21E-02	20/202 9.9%	828/17784 4.6%	*UGT1A1 RGS16 LGI3 WNT5A SAMD4A ITPR1 FOS SSTR2 FOSL1 CLMP DHRS9 SNPH CYP1A1 STX6 SLIT1 CYP1B1 OLR1 AHRR CRYAB VAMP2*
5624	membrane fraction	1.89E-03	1.66E-02	19/202 9.4%	797/17784 4.4%	*UGT1A1 RGS16 LGI3 WNT5A SAMD4A ITPR1 FOS SSTR2 FOSL1 CLMP DHRS9 SNPH CYP1A1 STX6 SLIT1 CYP1B1 OLR1 AHRR VAMP2*
34364	high-density lipoprotein particle	2.76E-03	2.20E-02	3/202 1.4%	25/17784 0.1%	*APOF SAA1 SAA2*

5886	plasma membrane	3.37E-03	2.61E-02	59/202 29.2%	3730/17784 20.9%	*OXTR CD83 CSF1 SERPINE1 ADRA1D SLC2A6 CX3CL1 ICAM1 ROBO1 RND1 RAB43 MMP25 SPRED1 PLAU OLR1 PGM5 CCR6 SLC34A3 VAV3 HS3ST3B1 FGFBP1 UNC13A UGT1A1 PCDHGA3 PRKCE TESC GPR37L1 SSTR2 RGMA BTC SLC7A5 MRAS SYPL1 SNPH CDH11 TLR7 ATP6V0D2 CRYAB SCN4B VAMP2 HBEGF ABCG2 ITPR1 TGFA DAPP1 TNFRSF11A GNAI1 RASGRP3 CLMP GJA10 STX6 S1PR3 KCNF1 TNFRSF9 LGI3 SAMD4A GJB2 SLC4A9 CD200*
44459	plasma membrane part	3.40E-03	2.61E-02	36/202 17.8%	1998/17784 11.2%	*OXTR CD83 ADRA1D TNFRSF11A GNAI1 ICAM1 ROBO1 RASGRP3 RND1 MMP25 SPRED1 CLMP GJA10 OLR1 PGM5 S1PR3 CCR6 SLC34A3 HS3ST3B1 KCNF1 UNC13A UGT1A1 TNFRSF9 LGI3 SAMD4A SSTR2 SLC7A5 GJB2 SLC4A9 SYPL1 SNPH ATP6V0D2 SCN4B CD200 VAMP2 HBEGF*
32994	protein-lipid complex	7.23E-03	4.35E-02	3/202 1.4%	35/17784 0.1%	*APOF SAA1 SAA2*
34358	plasma lipoprotein particle	7.23E-03	4.35E-02	3/202 1.4%	35/17784 0.1%	*APOF SAA1 SAA2*

[a] *p*-value in hypergeometric test after correction

[b] The denominator represents the total number of genes with GO annotation and the numerator represents the number of genes belonging to each GO term

[c] The denominator represents the number of reference genes with GO annotation and the numerator represents the number of references genes annotated in the listed GO terms

Table S7. Gene ontology (GO) enrichment analysis of differentially expressed genes between Caco-2 cells stimulated with CotG-p75 and control ($p < 0.00005$).

GO ID	GO description	*p*-value	Corrected *p*-value[a]	Cluster frequency[b]	Total frequency[c]	Genes
**Biological process**						
42221	response to chemical stimulus	1.14E-16	3.21E-13	50/164 30.4%	1466/17782 8.2%	*CD83 CXCL8 SERPINE1 CXCL1 CXCL3 ETS1 CXCL2 CX3CL1 ROBO1 PLAU CYP1B1 OLR1 MBL2 DUSP4 IGFBP1 UGT1A1 WNT5A PLA2G4A FOS WNT9A SSTR2 DUSP6 EREG ALDH1A3 IL1B AHRR AGXT ABCG2 PTAFR ITPR1 TNFRSF11A WNT6 IRAK2 UBD MBP UGT1A9 CCR10 WNT4 STAT5A EGR1 NOS2 CCL20 TGFB3 WNT7B FOSL1 NFKBIA CXCL10 CYP1A1 SAA1 SAA2*
9611	response to wounding	2.98E-16	4.18E-13	31/164 18.9%	544/17782 3.0%	*SERPINA3 CXCL8 SERPINE1 PTAFR CXCL1 CXCL3 CXCL2 CX3CL1 MMP25 PLAU IRAK2 TIFA OLR1 MBP MBL2 IGFBP1 SERPINB2 CCL20 TGFB3 PLA2G4B WNT5A GRHL3 FOS EREG CXCL10 IL1B CYP1A1 SAA1 AHRR SAA2 NFKBID*
48518	positive regulation of biological process	2.69E-14	2.51E-11	58/164 35.3%	2211/17782 12.4%	*CD83 CXCL8 CSF1 SERPINE1 SIX1 ADRA1D AREG ETS1 CX3CL1 ROBO1 FGF9 DPF1 LMCD1 PHLDA1 ICOSLG MBL2 VAV3 IGFBP1 MEF2C FGFBP1 PRKCE WNT5A PLA2G4A PROX1 FOS DUSP6 EREG ALDH1A3 IL1B IRF1 AHRR SOS1 TGFA TNFRSF11A RELB WNT6 IRAK2 UBD TIFA MBP DISC1 WNT4 STAT5A EGR1 NOS2 TGFB3 WNT7B TNFRSF9 SAMD4A FOXJ1 GRHL3 FOSL1 NFKBIA CXCL10 CYP1A1 SAA1 SAA2 NFKBID*
48522	positive regulation of cellular process	1.66E-12	1.17E-09	52/164 31.7%	2007/17782 11.2%	*CD83 CSF1 SERPINE1 SIX1 ADRA1D AREG ETS1 CX3CL1 ROBO1 FGF9 DPF1 LMCD1 PHLDA1 ICOSLG MBL2 VAV3 IGFBP1 MEF2C FGFBP1 PRKCE WNT5A PLA2G4A PROX1 FOS DUSP6 EREG ALDH1A3 IL1B IRF1 AHRR SOS1 TGFA TNFRSF11A UBD TIFA MBP DISC1 WNT4 STAT5A EGR1 TGFB3 WNT7B TNFRSF9 SAMD4A GRHL3 FOSL1 NFKBIA CXCL10 CYP1A1 SAA1 SAA2 NFKBID*

6952	defense response	3.02E-12	1.34E-09	28/164 17.0%	621/17782 3.4%	*SERPINA3 CD83 CXCL8 SERPINE1 PTAFR CXCL1 CXCL3 CXCL2 CX3CL1 MMP25 IRAK2 UBD TIFA OLR1 MBP ICOSLG MBL2 NOS2 CCL20 PLA2G4B WNT5A FOS FOSL1 CXCL10 IL1B SAA1 SAA2 NFKBID*
42330	taxis	3.60E-12	1.34E-09	16/164 9.7%	168/17782 0.9%	*CXCL8 CCL20 PTAFR CXCL1 TNFRSF11A CXCL3 CXCL2 CX3CL1 ROBO1 FOSL1 CXCL10 PLAU IL1B SAA1 SAA2 CCR10*
6935	chemotaxis	3.60E-12	1.34E-09	16/164 9.7%	168/17782 0.9%	*CXCL8 CCL20 PTAFR CXCL1 TNFRSF11A CXCL3 CXCL2 CX3CL1 ROBO1 FOSL1 CXCL10 PLAU IL1B SAA1 SAA2 CCR10*
7275	multicellular organismal development	6.48E-12	2.02E-09	64/164 39.0%	2971/17782 16.7%	*DYRK3 CXCL8 CSF1 SERPINE1 SIX1 CXCL1 ADRA1D CX3CL1 ROBO1 RND1 SPRED1 DNMT3L FGF9 PLAU GJA5 ZC3H12A DPF1 ADAMTS9 VAV3 IGFBP1 MEF2C UGT1A1 WNT5A HUNK PLA2G4A PROX1 FOS WNT9A TEX15 EREG SLC7A5 ALDH1A3 IL1B IRF1 CDH11 AHRR ITPR1 TNFRSF11A RELB WNT6 GBX2 UBD MBP NPTX1 DISC1 CCR10 WNT4 STAT5A EGR1 NOS2 TIPARP TGFB3 WNT7B CABLES1 FOXJ1 GRHL3 APLN CXCL10 RCAN1 DHRS9 MAFF CYP1A1 LHX4 SIM2*
9605	response to external stimulus	7.20E-12	2.02E-09	26/164 15.8%	551/17782 3.0%	*CXCL8 PTAFR CXCL1 TNFRSF11A CXCL3 CXCL2 CX3CL1 ROBO1 WNT6 PLAU CCR10 UGT1A1 CCL20 WNT7B WNT5A PLA2G4A FOS WNT9A SSTR2 FOSL1 CXCL10 IL1B CYP1A1 SAA1 AHRR SAA2*
51239	regulation of multicellular organismal process	8.07E-12	2.06E-09	36/164 21.9%	1068/17782 6.0%	*CD83 CSF1 SERPINE1 SIX1 TNFRSF11A ETS1 CX3CL1 ROBO1 WNT6 FGF9 PLAU MBP DISC1 LMCD1 WNT4 STAT5A EGR1 NOS2 TGFB3 WNT7B WNT5A PLA2G4A FOXJ1 PROX1 WNT9A SSTR2 EREG NFKBIA CXCL10 IL1B IRF1 TNNT3 MAFF SAA1 SAA2 NFKBID*
32502	developmental process	9.32E-12	2.18E-09	67/164 40.8%	3234/17782 18.1%	*DYRK3 CXCL8 CSF1 SERPINE1 SIX1 CXCL1 ADRA1D CX3CL1 ROBO1 RND1 SPRED1 DNMT3L FGF9 PLAU GJA5 ZC3H12A DPF1 ADAMTS9 IER3 VAV3 IGFBP1 MEF2C*

						TPRA1 UGT1A1 WNT5A HUNK PLA2G4A PROX1 FOS WNT9A TEX15 DUSP6 EREG SLC7A5 ALDH1A3 IL1B IRF1 CDH11 AHRR ITPR1 TNFRSF11A RELB WNT6 GBX2 UBD MBP NPTX1 DISC1 CCR10 WNT4 STAT5A EGR1 NOS2 TIPARP TGFB3 WNT7B CABLES1 FOXJ1 GRHL3 APLN CXCL10 RCAN1 DHRS9 MAFF CYP1A1 LHX4 SIM2
6954	inflammatory response	1.39E-11	2.99E-09	20/164 12.1%	317/17782 1.7%	*SERPINA3 CXCL8 CCL20 PLA2G4B PTAFR CXCL1 FOS CXCL3 CXCL2 CXCL10 MMP25 IRAK2 IL1B TIFA SAA1 OLR1 SAA2 MBP NFKBID MBL2*
40011	locomotion	1.63E-11	3.28E-09	23/164 14.0%	439/17782 2.4%	*VAV3 CXCL8 CCL20 DNAH5 PTAFR SIX1 FOXJ1 CXCL1 TNFRSF11A CXCL3 ETS1 CXCL2 CX3CL1 ROBO1 FOSL1 CXCL10 GBX2 PLAU IL1B SAA1 SAA2 DISC1 CCR10*
65007	biological regulation	2.54E-11	4.76E-09	106/164 64.6%	6941/17782 39.0%	*CD83 CSF1 SERPINE1 TBC1D3C ABCA12 ETS1 FGF9 PLAU DPF1 CYP1B1 ROS1 PHLDA1 ICOSLG IER3 IGFBP1 MEF2C ARL14 PRKCE WNT5A EREG ITPR1 TGFA TNFRSF11A RASGRP3 WNT6 IRAK2 UBD MBP SRGAP3 CCR10 WNT4 STAT5A EGR1 TGFB3 TNFRSF9 FOSL1 NFKBIA CXCL10 RCAN1 MAFF CYP1A1 LHX4 SIM2 NFKBID SERPINA3 DYRK3 CXCL8 STON1 SIX1 TNFAIP3 CXCL1 ADRA1D AREG CX3CL1 ROBO1 RND1 SPRED1 DNMT3L LMCD1 MBL2 DUSP4 VAV3 FGFBP1 SERPINB2 UGT1A1 PLA2G4B HUNK PLA2G4A PROX1 FOS WNT9A TEX15 SSTR2 DUSP6 ALDH1A3 SNPH IL1B IRF1 TNNT3 AHRR NCOA7 SOS1 BIRC3 PTAFR GNAI1 RELB GBX2 PALMD TIFA RGS22 DISC1 UGT1A9 NOS2 CCL20 GAD1 WNT7B LGI3 CNBP SAMD4A CABLES1 FOXJ1 GRHL3 APLN DHRS9 SAA1 SAA2*
2376	immune system process	2.96E-11	5.19E-09	33/164 20.1%	947/17782 5.3%	*DYRK3 CD83 CXCL8 CSF1 PTAFR SIX1 CXCL1 TNFRSF11A CXCL3 ETS1 CXCL2 CX3CL1 RELB IRAK2 UBD MBP ICOSLG CCR10 MBL2 STAT5A EGR1 NOS2 TIPARP CCL20 WNT5A FOXJ1 APLN NFKBIA CXCL10 IL1B IRF1 SAA1 SAA2*
9617	response to bacterium	5.01E-11	7.81E-09	17/164	232/17782	*UGT1A1 NOS2 CCL20 SERPINE1 PTAFR WNT5A PLA2G4A*

				10.3%	1.3%	*FOS TNFRSF11A NFKBIA CXCL10 IRAK2 IL1B CYP1A1 AHRR MBP MBL2*
48731	system development	5.71E-11	8.20E-09	55/164 33.5%	2423/17782 13.6%	*DYRK3 CXCL8 CSF1 SERPINE1 SIX1 CXCL1 CX3CL1 ROBO1 RND1 FGF9 PLAU GJA5 ZC3H12A DPF1 VAV3 IGFBP1 MEF2C UGT1A1 WNT5A PLA2G4A PROX1 FOS WNT9A TEX15 EREG SLC7A5 ALDH1A3 IRF1 CDH11 AHRR TNFRSF11A RELB WNT6 GBX2 UBD MBP NPTX1 DISC1 WNT4 STAT5A EGR1 NOS2 TIPARP TGFB3 WNT7B CABLES1 FOXJ1 GRHL3 APLN CXCL10 RCAN1 DHRS9 CYP1A1 LHX4 SIM2*
32496	response to lipopolysaccharide	5.85E-11	8.20E-09	13/164 7.9%	117/17782 0.6%	*UGT1A1 SERPINE1 PTAFR WNT5A PLA2G4A FOS TNFRSF11A NFKBIA CXCL10 IRAK2 IL1B CYP1A1 AHRR*
70887	cellular response to chemical stimulus	1.35E-10	1.81E-08	21/164 12.8%	401/17782 2.2%	*STAT5A IGFBP1 EGR1 CXCL8 UGT1A1 WNT7B SERPINE1 WNT5A FOS WNT9A TNFRSF11A CXCL3 CX3CL1 WNT6 IL1B CYP1A1 SAA1 AHRR SAA2 UGT1A9 WNT4*
2237	response to molecule of bacterial origin	1.83E-10	2.34E-08	13/164 7.9%	128/17782 0.7%	*UGT1A1 SERPINE1 PTAFR WNT5A PLA2G4A FOS TNFRSF11A NFKBIA CXCL10 IRAK2 IL1B CYP1A1 AHRR*
32501	multicellular organismal process	3.77E-10	4.61E-08	77/164 46.9%	4376/17782 24.6%	*SERPINA3 DYRK3 CXCL8 CSF1 SERPINE1 SIX1 CXCL1 ADRA1D CX3CL1 ROBO1 RND1 SPRED1 DNMT3L FGF9 PLAU GJA5 ZC3H12A DPF1 CYP1B1 OLR1 ROS1 ADAMTS9 VAV3 IGFBP1 MEF2C UGT1A1 WNT5A HUNK PLA2G4A PROX1 FOS WNT9A TEX15 SSTR2 EREG SLC7A5 ALDH1A3 SNPH IL1B IRF1 TNNT3 CDH11 AHRR BIRC3 PTAFR ITPR1 TNFRSF11A RELB WNT6 GBX2 UBD MBP NPTX1 DISC1 CCR10 WNT4 STAT5A EGR1 NOS2 TIPARP TGFB3 GAD1 WNT7B CABLES1 FOXJ1 GRHL3 APLN FOSL1 CXCL10 RCAN1 DHRS9 MAFF CYP1A1 SAA1 SAA2 LHX4 SIM2*
48856	anatomical structure development	6.26E-10	7.33E-08	56/164 34.1%	2657/17782 14.9%	*DYRK3 CXCL8 CSF1 SERPINE1 SIX1 CXCL1 CX3CL1 ROBO1 RND1 FGF9 PLAU GJA5 ZC3H12A DPF1 IER3 VAV3 IGFBP1 MEF2C UGT1A1 WNT5A PLA2G4A PROX1 FOS WNT9A TEX15 EREG SLC7A5 ALDH1A3 IRF1 CDH11 AHRR*

						TNFRSF11A RELB WNT6 GBX2 UBD MBP NPTX1 DISC1 WNT4 STAT5A EGR1 NOS2 TIPARP TGFB3 WNT7B CABLES1 FOXJ1 GRHL3 APLN CXCL10 RCAN1 DHRS9 CYP1A1 LHX4 SIM2
50789	regulation of biological process	7.14E-10	8.02E-08	99/164 60.3%	6552/17782 36.8%	*CD83 CSF1 SERPINE1 TBC1D3C ETS1 FGF9 PLAU DPF1 ROS1 PHLDA1 ICOSLG IER3 IGFBP1 MEF2C ARL14 PRKCE WNT5A EREG ITPR1 TGFA TNFRSF11A RASGRP3 WNT6 IRAK2 UBD MBP SRGAP3 WNT4 STAT5A EGR1 TGFB3 TNFRSF9 FOSL1 NFKBIA CXCL10 RCAN1 MAFF CYP1A1 LHX4 SIM2 NFKBID SERPINA3 CXCL8 STON1 SIX1 TNFAIP3 CXCL1 ADRA1D AREG CX3CL1 ROBO1 RND1 SPRED1 DNMT3L LMCD1 MBL2 DUSP4 VAV3 FGFBP1 SERPINB2 UGT1A1 PLA2G4B HUNK PLA2G4A PROX1 FOS WNT9A TEX15 SSTR2 DUSP6 ALDH1A3 IL1B IRF1 TNNT3 AHRR NCOA7 SOS1 BIRC3 PTAFR GNAI1 RELB GBX2 PALMD TIFA RGS22 DISC1 UGT1A9 NOS2 CCL20 WNT7B LGI3 CNBP SAMD4A CABLES1 FOXJ1 GRHL3 APLN SAA1 SAA2*
42127	regulation of cell proliferation	8.72E-10	9.41E-08	29/164 17.6%	848/17782 4.7%	*CXCL8 CSF1 SERPINE1 TGFA CXCL1 ADRA1D TNFRSF11A ETS1 ROBO1 FGF9 PLAU DISC1 ICOSLG STAT5A FGFBP1 NOS2 TGFB3 TNFRSF9 WNT5A PLA2G4A FOXJ1 PROX1 WNT9A SSTR2 EREG FOSL1 NFKBIA CXCL10 IL1B*
50794	regulation of cellular process	1.49E-09	1.54E-07	95/164 57.9%	6222/17782 34.9%	*CD83 CXCL8 CSF1 SERPINE1 STON1 SIX1 TNFAIP3 TBC1D3C CXCL1 ADRA1D AREG ETS1 CX3CL1 ROBO1 RND1 SPRED1 FGF9 PLAU DPF1 LMCD1 ROS1 PHLDA1 ICOSLG IER3 MBL2 DUSP4 VAV3 IGFBP1 MEF2C FGFBP1 SERPINB2 ARL14 PRKCE PLA2G4B WNT5A HUNK PLA2G4A PROX1 FOS WNT9A TEX15 SSTR2 DUSP6 EREG ALDH1A3 IL1B IRF1 TNNT3 AHRR NCOA7 SOS1 BIRC3 PTAFR ITPR1 TGFA TNFRSF11A GNAI1 RELB RASGRP3 GBX2 PALMD IRAK2 UBD TIFA MBP SRGAP3 RGS22 DISC1 UGT1A9 WNT4 STAT5A EGR1 NOS2 CCL20 TGFB3 WNT7B TNFRSF9 LGI3 CNBP SAMD4A CABLES1 FOXJ1 GRHL3*

						APLN FOSL1 NFKBIA CXCL10 RCAN1 MAFF CYP1A1 SAA1 SAA2 LHX4 SIM2 NFKBID
48513	organ development	2.89E-09	2.80E-07	43/164 26.2%	1793/17782 10.0%	*DYRK3 CXCL8 CSF1 SERPINE1 SIX1 TNFRSF11A CX3CL1 ROBO1 RELB WNT6 GBX2 FGF9 PLAU GJA5 UBD ZC3H12A WNT4 STAT5A VAV3 IGFBP1 EGR1 MEF2C UGT1A1 NOS2 TIPARP TGFB3 WNT7B WNT5A PLA2G4A GRHL3 PROX1 WNT9A TEX15 EREG APLN CXCL10 RCAN1 ALDH1A3 DHRS9 IRF1 CYP1A1 AHRR LHX4*
23046	signaling process	2.99E-09	2.80E-07	48/164 29.2%	2156/17782 12.1%	*CD83 CXCL8 PTAFR ITPR1 TGFA CXCL1 ADRA1D TNFRSF11A CX3CL1 GNAI1 RASGRP3 RND1 SPRED1 FGF9 PLAU IRAK2 TIFA MBP NPTX1 SRGAP3 ROS1 ICOSLG STAT5A DUSP4 VAV3 IGFBP1 FGFBP1 ARL14 NOS2 CCL20 TGFB3 PRKCE GAD1 PLA2G4B WNT5A HUNK FOS WNT9A SSTR2 DUSP6 EREG APLN NFKBIA CXCL10 RCAN1 SNPH IL1B SOS1*
23060	signal transmission	2.99E-09	2.80E-07	48/164 29.2%	2156/17782 12.1%	*CD83 CXCL8 PTAFR ITPR1 TGFA CXCL1 ADRA1D TNFRSF11A CX3CL1 GNAI1 RASGRP3 RND1 SPRED1 FGF9 PLAU IRAK2 TIFA MBP NPTX1 SRGAP3 ROS1 ICOSLG STAT5A DUSP4 VAV3 IGFBP1 FGFBP1 ARL14 NOS2 CCL20 TGFB3 PRKCE GAD1 PLA2G4B WNT5A HUNK FOS WNT9A SSTR2 DUSP6 EREG APLN NFKBIA CXCL10 RCAN1 SNPH IL1B SOS1*
51094	positive regulation of developmental process	3.58E-09	3.16E-07	18/164 10.9%	347/17782 1.9%	*STAT5A CD83 CSF1 TGFB3 WNT7B SERPINE1 WNT5A SIX1 PLA2G4A PROX1 ETS1 CX3CL1 ROBO1 WNT6 FGF9 IL1B DISC1 WNT4*
7165	signal transduction	3.60E-09	3.16E-07	44/164 26.8%	1877/17782 10.5%	*CD83 CXCL8 PTAFR ITPR1 TGFA CXCL1 ADRA1D TNFRSF11A CX3CL1 GNAI1 RASGRP3 RND1 SPRED1 FGF9 PLAU IRAK2 TIFA SRGAP3 ROS1 ICOSLG STAT5A DUSP4 VAV3 IGFBP1 FGFBP1 ARL14 NOS2 CCL20 TGFB3 PRKCE PLA2G4B WNT5A HUNK FOS WNT9A SSTR2 DUSP6 EREG APLN NFKBIA CXCL10 RCAN1 IL1B SOS1*

50793	regulation of developmental process	3.76E-09	3.20E-07	27/164 16.4%	791/17782 4.4%	*CD83 CSF1 SERPINE1 SIX1 ETS1 CX3CL1 ROBO1 WNT6 FGF9 PALMD MBP DISC1 WNT4 STAT5A TGFB3 WNT7B WNT5A PLA2G4A FOXJ1 PROX1 WNT9A EREG NFKBIA CXCL10 IL1B MAFF NFKBID*
42981	regulation of apoptosis	4.27E-09	3.53E-07	28/164 17.0%	852/17782 4.7%	*SERPINE1 SIX1 TNFAIP3 ETS1 CX3CL1 ROBO1 UBD DPF1 PHLDA1 IER3 STAT5A VAV3 MEF2C SERPINB2 TGFB3 PRKCE TNFRSF9 WNT5A PLA2G4A DUSP6 FOSL1 NFKBIA ALDH1A3 IL1B LHX4 SOS1 NFKBID BIRC3*
7626	locomotory behavior	4.70E-09	3.77E-07	16/164 9.7%	272/17782 1.5%	*CXCL8 CCL20 PTAFR CXCL1 TNFRSF11A CXCL3 CXCL2 CX3CL1 ROBO1 FOSL1 CXCL10 PLAU IL1B SAA1 SAA2 CCR10*
43067	regulation of programmed cell death	5.24E-09	4.09E-07	28/164 17.0%	860/17782 4.8%	*SERPINE1 SIX1 TNFAIP3 ETS1 CX3CL1 ROBO1 UBD DPF1 PHLDA1 IER3 STAT5A VAV3 MEF2C SERPINB2 TGFB3 PRKCE TNFRSF9 WNT5A PLA2G4A DUSP6 FOSL1 NFKBIA ALDH1A3 IL1B LHX4 SOS1 NFKBID BIRC3*
51707	response to other organism	5.83E-09	4.42E-07	18/164 10.9%	358/17782 2.0%	*UGT1A1 NOS2 CCL20 SERPINE1 PTAFR WNT5A PLA2G4A FOS TNFRSF11A FOSL1 NFKBIA CXCL10 IRAK2 IL1B CYP1A1 AHRR MBP MBL2*
10941	regulation of cell death	6.25E-09	4.62E-07	28/164 17.0%	867/17782 4.8%	*SERPINE1 SIX1 TNFAIP3 ETS1 CX3CL1 ROBO1 UBD DPF1 PHLDA1 IER3 STAT5A VAV3 MEF2C SERPINB2 TGFB3 PRKCE TNFRSF9 WNT5A PLA2G4A DUSP6 FOSL1 NFKBIA ALDH1A3 IL1B LHX4 SOS1 NFKBID BIRC3*
51240	positive regulation of multicellular organismal process	6.75E-09	4.86E-07	16/164 9.7%	279/17782 1.5%	*STAT5A CD83 CSF1 TGFB3 SERPINE1 WNT5A PLA2G4A TNFRSF11A CX3CL1 EREG WNT6 FGF9 IL1B SAA1 SAA2 WNT4*
42573	retinoic acid metabolic process	9.51E-09	6.67E-07	6/164 3.6%	18/17782 0.1%	*ALDH1A3 DHRS9 UGT1A1 CYP1A1 AHRR UGT1A9*
48519	negative regulation of biological process	1.11E-08	7.63E-07	45/164 27.4%	2021/17782 11.3%	*CD83 CXCL8 SERPINE1 SIX1 TNFAIP3 CXCL1 ETS1 CX3CL1 ROBO1 RND1 FGF9 PLAU UBD MBP RGS22 UGT1A9 LMCD1 ROS1 IER3 WNT4 MBL2 STAT5A EGR1*

						MEF2C FGFBP1 SERPINB2 UGT1A1 TGFB3 TNFRSF9 WNT5A FOXJ1 PROX1 WNT9A SSTR2 DUSP6 EREG FOSL1 NFKBIA CXCL10 IL1B SAA1 SAA2 LHX4 NFKBID BIRC3
23052	signaling	1.28E-08	8.36E-07	59/164 35.9%	3127/17782 17.5%	*CD83 CXCL8 SIX1 CXCL1 ADRA1D AREG CX3CL1 ROBO1 RND1 SPRED1 FGF9 PLAU GJA5 ROS1 ICOSLG DUSP4 VAV3 IGFBP1 FGFBP1 ARL14 PRKCE PLA2G4B WNT5A HUNK FOS WNT9A SSTR2 DUSP6 EREG SNPH IL1B SOS1 BIRC3 NXPH3 PTAFR ITPR1 TGFA TNFRSF11A GNAI1 RASGRP3 WNT6 IRAK2 TIFA MBP NPTX1 SRGAP3 CCR10 WNT4 STAT5A NOS2 TIPARP CCL20 TGFB3 GAD1 WNT7B APLN NFKBIA CXCL10 RCAN1*
30593	neutrophil chemotaxis	1.38E-08	8.80E-07	6/164 3.6%	19/17782 0.1%	*CXCL8 IL1B SAA1 SAA2 CXCL3 CX3CL1*
48583	regulation of response to stimulus	1.72E-08	1.07E-06	21/164 12.8%	526/17782 2.9%	*STAT5A CXCL8 NOS2 SERPINE1 WNT5A PLA2G4A FOXJ1 TEX15 TNFRSF11A CX3CL1 EREG ROBO1 NFKBIA CXCL10 PLAU IRAK2 IL1B SAA1 SAA2 MBP MBL2*
6950	response to stress	2.21E-08	1.35E-06	41/164 25.0%	1775/17782 9.9%	*SERPINA3 CD83 CXCL8 SERPINE1 PTAFR ITPR1 CXCL1 CXCL3 CXCL2 CX3CL1 MMP25 PLAU IRAK2 UBD TIFA OLR1 MBP ICOSLG MBL2 IGFBP1 SERPINB2 UGT1A1 NOS2 CCL20 TGFB3 PLA2G4B WNT5A PLA2G4A GRHL3 FOS DUSP6 EREG FOSL1 CXCL10 IL1B MAFF CYP1A1 SAA1 AHRR SAA2 NFKBID*
32101	regulation of response to external stimulus	2.66E-08	1.59E-06	13/164 7.9%	192/17782 1.0%	*STAT5A CXCL8 SERPINE1 WNT5A PLA2G4A TNFRSF11A CX3CL1 ROBO1 CXCL10 PLAU IL1B SAA1 SAA2*
9812	flavonoid metabolic process	2.77E-08	1.62E-06	5/164 3.0%	11/17782 0.0%	*UGT1A10 UGT1A1 CYP1A1 AHRR UGT1A9*
9966	regulation of signal transduction	4.39E-08	2.51E-06	25/164 15.2%	773/17782 4.3%	*CSF1 SERPINE1 TGFA TNFAIP3 TBC1D3C TNFRSF11A ROBO1 RASGRP3 SPRED1 FGF9 PLAU IRAK2 UBD TIFA RGS22 LMCD1 VAV3 TGFB3 WNT5A FOXJ1 DUSP6 EREG IL1B SOS1 NFKBID*

23051	regulation of signaling process	4.97E-08	2.79E-06	25/164 15.2%	778/17782 4.3%	*CSF1 SERPINE1 TGFA TNFAIP3 TBC1D3C TNFRSF11A ROBO1 RASGRP3 SPRED1 FGF9 PLAU IRAK2 UBD TIFA RGS22 LMCD1 VAV3 TGFB3 WNT5A FOXJ1 DUSP6 EREG IL1B SOS1 NFKBID*
7166	cell surface receptor linked signaling pathway	5.53E-08	3.04E-06	33/164 20.1%	1278/17782 7.1%	*CXCL8 NXPH3 PTAFR CXCL1 ADRA1D TNFRSF11A AREG CX3CL1 GNAI1 ROBO1 WNT6 FGF9 IRAK2 ROS1 CCR10 WNT4 STAT5A VAV3 IGFBP1 NOS2 TIPARP TGFB3 WNT7B WNT5A FOS WNT9A SSTR2 EREG NFKBIA CXCL10 IL1B SOS1 BIRC3*
10646	regulation of cell communication	6.25E-08	3.37E-06	31/164 18.9%	1154/17782 6.4%	*CSF1 SERPINE1 TGFA TNFAIP3 TBC1D3C TNFRSF11A ROBO1 RASGRP3 SPRED1 FGF9 PLAU IRAK2 UBD TIFA RGS22 DISC1 LMCD1 WNT4 VAV3 EGR1 FGFBP1 NOS2 TGFB3 WNT5A FOXJ1 DUSP6 EREG NFKBIA IL1B SOS1 NFKBID*
31349	positive regulation of defense response	7.10E-08	3.76E-06	9/164 5.4%	83/17782 0.4%	*STAT5A NFKBIA IRAK2 IL1B SERPINE1 PLA2G4A TNFRSF11A CX3CL1 EREG*
9698	phenylpropanoid metabolic process	7.61E-08	3.96E-06	5/164 3.0%	13/17782 0.0%	*UGT1A10 UGT1A1 CYP1A1 AHRR UGT1A9*
19748	secondary metabolic process	9.77E-08	4.99E-06	8/164 4.8%	62/17782 0.3%	*UGT1A10 ALDH1A3 DHRS9 UGT1A1 CYP1A1 CYP1B1 AHRR UGT1A9*
23033	signaling pathway	1.00E-07	5.02E-06	44/164 26.8%	2098/17782 11.7%	*CXCL8 NXPH3 PTAFR TGFA CXCL1 ADRA1D TNFRSF11A AREG CX3CL1 GNAI1 ROBO1 RASGRP3 RND1 WNT6 SPRED1 FGF9 IRAK2 TIFA ROS1 CCR10 WNT4 STAT5A DUSP4 VAV3 IGFBP1 ARL14 NOS2 TIPARP TGFB3 PRKCE WNT7B PLA2G4B WNT5A FOS WNT9A SSTR2 DUSP6 EREG NFKBIA CXCL10 RCAN1 IL1B SOS1 BIRC3*
30879	mammary gland development	1.07E-07	5.12E-06	9/164 5.4%	87/17782 0.4%	*STAT5A CSF1 TGFB3 WNT7B WNT5A TNFRSF11A ROBO1 WNT4 APLN*
32103	positive regulation of response to external stimulus	1.07E-07	5.12E-06	9/164 5.4%	87/17782 0.4%	*STAT5A CXCL10 CXCL8 IL1B SERPINE1 WNT5A PLA2G4A TNFRSF11A CX3CL1*

10033	response to organic substance	1.10E-07	5.12E-06	26/164 15.8%	870/17782 4.8%	*CD83 SERPINE1 PTAFR TNFRSF11A IRAK2 UBD CYP1B1 WNT4 STAT5A DUSP4 IGFBP1 EGR1 UGT1A1 TGFB3 WNT5A PLA2G4A FOS DUSP6 EREG FOSL1 NFKBIA CXCL10 IL1B CYP1A1 AHRR AGXT*
50900	leukocyte migration	1.11E-07	5.12E-06	8/164 4.8%	63/17782 0.3%	*CXCL8 IL1B SAA1 SAA2 FOXJ1 TNFRSF11A CXCL3 CX3CL1*
7267	cell-cell signaling	1.11E-07	5.12E-06	21/164 12.8%	587/17782 3.3%	*FGFBP1 CCL20 GAD1 WNT7B SIX1 ADRA1D WNT9A TNFRSF11A SSTR2 AREG EREG WNT6 CXCL10 FGF9 SNPH GJA5 IL1B TIFA MBP NPTX1 WNT4*
30595	leukocyte chemotaxis	1.22E-07	5.51E-06	7/164 4.2%	43/17782 0.2%	*CXCL8 IL1B SAA1 SAA2 TNFRSF11A CXCL3 CX3CL1*
48514	blood vessel morphogenesis	1.32E-07	5.88E-06	13/164 7.9%	220/17782 1.2%	*VAV3 CXCL8 NOS2 TIPARP WNT7B PROX1 CX3CL1 EREG ROBO1 GBX2 FGF9 PLAU ZC3H12A*
30155	regulation of cell adhesion	1.35E-07	5.93E-06	11/164 6.7%	149/17782 0.8%	*STAT5A VAV3 CXCL8 CSF1 PLAU IL1B SERPINE1 SAA1 SAA2 CX3CL1 RND1*
1568	blood vessel development	1.71E-07	7.25E-06	14/164 8.5%	265/17782 1.4%	*VAV3 CXCL8 NOS2 TIPARP WNT7B PROX1 CX3CL1 EREG ROBO1 GBX2 FGF9 PLAU GJA5 ZC3H12A*
9607	response to biotic stimulus	1.77E-07	7.37E-06	18/164 10.9%	448/17782 2.5%	*UGT1A1 NOS2 CCL20 SERPINE1 PTAFR WNT5A PLA2G4A FOS TNFRSF11A FOSL1 NFKBIA CXCL10 IRAK2 IL1B CYP1A1 AHRR MBP MBL2*
2684	positive regulation of immune system process	1.79E-07	7.37E-06	14/164 8.5%	266/17782 1.4%	*STAT5A CD83 NOS2 SERPINE1 WNT5A FOXJ1 EREG NFKBIA CXCL10 IRAK2 IL1B MBP ICOSLG MBL2*
34754	cellular hormone metabolic process	1.81E-07	7.37E-06	8/164 4.8%	67/17782 0.3%	*ALDH1A3 DHRS9 UGT1A1 CYP1A1 CYP1B1 AHRR UGT1A9 WNT4*
45595	regulation of cell differentiation	1.93E-07	7.72E-06	20/164 12.1%	553/17782 3.1%	*STAT5A CD83 CSF1 TGFB3 WNT7B WNT5A SIX1 FOXJ1 PROX1 WNT9A ETS1 EREG ROBO1 NFKBIA FGF9 MAFF MBP DISC1 NFKBID WNT4*
48584	positive regulation of response	2.05E-07	8.00E-06	14/164	269/17782	*STAT5A CXCL8 SERPINE1 WNT5A PLA2G4A TNFRSF11A*

	to stimulus			8.5%	1.5%	*CX3CL1 EREG NFKBIA CXCL10 IRAK2 IL1B MBP MBL2*
50896	response to stimulus	2.05E-07	8.00E-06	62/164 37.8%	3632/17782 20.4%	*SERPINA3 CD83 CXCL8 SERPINE1 CXCL1 CXCL3 ETS1 CXCL2 CX3CL1 ROBO1 MMP25 PLAU CYP1B1 OLR1 ICOSLG MBL2 DUSP4 IGFBP1 SERPINB2 UGT1A1 PLA2G4B WNT5A PLA2G4A FOS WNT9A SSTR2 DUSP6 EREG ALDH1A3 IL1B AHRR AGXT ABCG2 PTAFR ITPR1 TNFRSF11A RELB WNT6 IRAK2 UBD TIFA MBP UGT1A9 CCR10 WNT4 STAT5A EGR1 NOS2 CCL20 TGFB3 WNT7B FOXJ1 GRHL3 APLN FOSL1 NFKBIA CXCL10 MAFF CYP1A1 SAA1 SAA2 NFKBID*
51674	localization of cell	2.34E-07	8.75E-06	15/164 9.1%	315/17782 1.7%	*VAV3 CXCL8 DNAH5 SIX1 FOXJ1 TNFRSF11A CXCL3 ETS1 CX3CL1 ROBO1 GBX2 IL1B SAA1 SAA2 DISC1*
48870	cell motility	2.34E-07	8.75E-06	15/164 9.1%	315/17782 1.7%	*VAV3 CXCL8 DNAH5 SIX1 FOXJ1 TNFRSF11A CXCL3 ETS1 CX3CL1 ROBO1 GBX2 IL1B SAA1 SAA2 DISC1*
51704	multi-organism process	2.43E-07	8.94E-06	24/164 14.6%	786/17782 4.4%	*STAT5A UGT1A1 NOS2 CCL20 TGFB3 PLA2G4B SERPINE1 PTAFR WNT5A PLA2G4A FOS TNFRSF11A FOSL1 NFKBIA CXCL10 PLAU IRAK2 IL1B MAFF CYP1A1 AHRR MBP WNT4 MBL2*
1944	vasculature development	2.45E-07	8.94E-06	14/164 8.5%	273/17782 1.5%	*VAV3 CXCL8 NOS2 TIPARP WNT7B PROX1 CX3CL1 EREG ROBO1 GBX2 FGF9 PLAU GJA5 ZC3H12A*
6955	immune response	2.55E-07	9.17E-06	21/164 12.8%	617/17782 3.4%	*CD83 CXCL8 NOS2 CCL20 PTAFR WNT5A FOXJ1 CXCL1 TNFRSF11A CXCL3 ETS1 CXCL2 CX3CL1 RELB APLN CXCL10 IL1B UBD MBP CCR10 MBL2*
60326	cell chemotaxis	2.67E-07	9.50E-06	7/164 4.2%	48/17782 0.2%	*CXCL8 IL1B SAA1 SAA2 TNFRSF11A CXCL3 CX3CL1*
1523	retinoid metabolic process	2.78E-07	9.50E-06	6/164 3.6%	30/17782 0.1%	*ALDH1A3 DHRS9 UGT1A1 CYP1A1 AHRR UGT1A9*
6776	vitamin A metabolic process	2.78E-07	9.50E-06	6/164 3.6%	30/17782 0.1%	*ALDH1A3 DHRS9 UGT1A1 CYP1A1 AHRR UGT1A9*

16101	diterpenoid metabolic process	2.78E-07	9.50E-06	6/164 3.6%	30/17782 0.1%	*ALDH1A3 DHRS9 UGT1A1 CYP1A1 AHRR UGT1A9*
45787	positive regulation of cell cycle	2.87E-07	9.69E-06	8/164 4.8%	71/17782 0.3%	*STAT5A FOSL1 IL1B CYP1A1 TGFA AHRR PROX1 EREG*
7610	behavior	3.07E-07	1.02E-05	18/164 10.9%	465/17782 2.6%	*EGR1 CXCL8 CCL20 PTAFR CXCL1 FOS TNFRSF11A CXCL3 CXCL2 CX3CL1 ROBO1 FOSL1 CXCL10 PLAU IL1B SAA1 SAA2 CCR10*
31347	regulation of defense response	3.16E-07	1.04E-05	11/164 6.7%	162/17782 0.9%	*STAT5A NFKBIA IRAK2 IL1B SERPINE1 SAA1 SAA2 PLA2G4A TNFRSF11A CX3CL1 EREG*
42060	wound healing	3.46E-07	1.13E-05	12/164 7.3%	200/17782 1.1%	*IGFBP1 SERPINB2 PLAU TGFB3 IL1B SERPINE1 WNT5A SAA1 SAA2 GRHL3 CX3CL1 EREG*
22414	reproductive process	4.01E-07	1.28E-05	24/164 14.6%	808/17782 4.5%	*STAT5A CSF1 NOS2 TGFB3 PLA2G4B WNT5A PLA2G4A FOXJ1 FOS TEX15 EREG APLN FOSL1 DNMT3L FGF9 PLAU IL1B MAFF CYP1A1 AHRR DISC1 ROS1 WNT4 BIRC3*
2682	regulation of immune system process	4.23E-07	1.33E-05	17/164 10.3%	425/17782 2.3%	*STAT5A CD83 CSF1 NOS2 SERPINE1 WNT5A FOXJ1 ETS1 EREG NFKBIA CXCL10 IRAK2 IL1B MBP NFKBID ICOSLG MBL2*
3	reproduction	4.28E-07	1.34E-05	24/164 14.6%	811/17782 4.5%	*STAT5A CSF1 NOS2 TGFB3 PLA2G4B WNT5A PLA2G4A FOXJ1 FOS TEX15 EREG APLN FOSL1 DNMT3L FGF9 PLAU IL1B MAFF CYP1A1 AHRR DISC1 ROS1 WNT4 BIRC3*
7154	cell communication	4.38E-07	1.35E-05	24/164 14.6%	812/17782 4.5%	*FGFBP1 CCL20 GAD1 WNT7B WNT5A SIX1 ADRA1D FOS WNT9A TNFRSF11A SSTR2 AREG EREG FOSL1 WNT6 CXCL10 FGF9 SNPH GJA5 IL1B TIFA MBP NPTX1 WNT4*
33273	response to vitamin	4.64E-07	1.41E-05	9/164 5.4%	103/17782 0.5%	*WNT6 CXCL10 IL1B WNT7B WNT5A CYP1A1 AHRR PLA2G4A WNT9A*
7584	response to nutrient	4.83E-07	1.44E-05	11/164 6.7%	169/17782 0.9%	*WNT6 CXCL10 UGT1A1 IL1B WNT7B WNT5A CYP1A1 AHRR PLA2G4A WNT9A SSTR2*
35556	intracellular signal transduction	6.92E-07	2.02E-05	24/164	833/17782	*STAT5A DUSP4 VAV3 CXCL8 ARL14 NOS2 TGFB3 PLA2G4B*

				14.6%	4.6%	*PTAFR WNT5A TGFA ADRA1D SSTR2 DUSP6 GNAI1 RASGRP3 RND1 NFKBIA RCAN1 SPRED1 IRAK2 IL1B TIFA SOS1*
9991	response to extracellular stimulus	1.10E-06	3.20E-05	13/164 7.9%	265/17782 1.4%	*UGT1A1 WNT7B WNT5A PLA2G4A FOS WNT9A SSTR2 FOSL1 WNT6 CXCL10 IL1B CYP1A1 AHRR*
7223	Wnt receptor signaling pathway, calcium modulating pathway	1.13E-06	3.25E-05	5/164 3.0%	21/17782 0.1%	*WNT6 WNT7B WNT5A WNT9A WNT4*
6721	terpenoid metabolic process	1.21E-06	3.44E-05	6/164 3.6%	38/17782 0.2%	*ALDH1A3 DHRS9 UGT1A1 CYP1A1 AHRR UGT1A9*
65008	regulation of biological quality	1.41E-06	3.94E-05	34/164 20.7%	1544/17782 8.6%	*SERPINA3 DYRK3 CSF1 SERPINE1 ITPR1 TNFRSF11A ABCA12 PALMD PLAU CYP1B1 MBP UGT1A9 CCR10 WNT4 MBL2 STAT5A IGFBP1 EGR1 UGT1A1 NOS2 TGFB3 GAD1 WNT7B PLA2G4A TEX15 NFKBIA ALDH1A3 DHRS9 SNPH IL1B CYP1A1 SAA1 AHRR SAA2*
35567	non-canonical Wnt receptor signaling pathway	1.46E-06	4.05E-05	5/164 3.0%	22/17782 0.1%	*WNT6 WNT7B WNT5A WNT9A WNT4*
80134	regulation of response to stress	1.64E-06	4.44E-05	14/164 8.5%	320/17782 1.7%	*STAT5A SERPINE1 WNT5A PLA2G4A TEX15 TNFRSF11A CX3CL1 EREG NFKBIA PLAU IRAK2 IL1B SAA1 SAA2*
35466	regulation of signaling pathway	1.65E-06	4.44E-05	26/164 15.8%	1003/17782 5.6%	*CSF1 TGFA TNFAIP3 TBC1D3C TNFRSF11A ROBO1 RASGRP3 SPRED1 FGF9 IRAK2 UBD TIFA DISC1 LMCD1 WNT4 VAV3 FGFBP1 TGFB3 WNT5A FOXJ1 DUSP6 EREG NFKBIA IL1B SOS1 NFKBID*
6775	fat-soluble vitamin metabolic process	1.66E-06	4.44E-05	6/164 3.6%	40/17782 0.2%	*ALDH1A3 DHRS9 UGT1A1 CYP1A1 AHRR UGT1A9*
6953	acute-phase response	1.66E-06	4.44E-05	6/164 3.6%	40/17782 0.2%	*SERPINA3 TIFA SAA1 SAA2 MBP MBL2*
48732	gland development	1.80E-06	4.75E-05	11/164 6.7%	193/17782 1.0%	*STAT5A ALDH1A3 CSF1 TGFB3 WNT7B WNT5A SIX1 TNFRSF11A ROBO1 WNT4 APLN*

**Molecular function**						
5126	cytokine receptor binding	1.58E-07	6.82E-06	12/164 7.3%	186/17782 1.0%	*CXCL10 SPRED1 CXCL8 CSF1 CCL20 TGFB3 IL1B TIFA CXCL1 CXCL3 CXCL2 CX3CL1*
8009	chemokine activity	2.30E-07	8.75E-06	7/164 4.2%	47/17782 0.2%	*CXCL10 CXCL8 CCL20 CXCL1 CXCL3 CXCL2 CX3CL1*
5102	receptor binding	3.52E-07	1.14E-05	26/164 15.8%	924/17782 5.1%	*CXCL8 CSF1 TGFA CXCL1 CXCL3 AREG CXCL2 CX3CL1 SPRED1 FGF9 TIFA APOF MBP ICOSLG MBL2 VAV3 CCL20 TGFB3 WNT5A PROX1 EREG APLN CXCL10 IL1B SAA1 SAA2*
42379	chemokine receptor binding	4.71E-07	1.42E-05	7/164 4.2%	52/17782 0.2%	*CXCL10 CXCL8 CCL20 CXCL1 CXCL3 CXCL2 CX3CL1*
1664	G-protein-coupled receptor binding	5.30E-07	1.57E-05	10/164 6.0%	136/17782 0.7%	*CXCL10 CXCL8 CCL20 WNT5A SAA1 SAA2 CXCL1 CXCL3 CXCL2 CX3CL1*
**Cellular component**						
44421	extracellular region part	3.81E-12	1.34E-09	35/164 21.3%	985/17782 5.5%	*CXCL8 CSF1 SERPINE1 TGFA CXCL1 CXCL3 AREG CXCL2 CX3CL1 WNT6 MMP25 MUC2 FGF9 PLAU TIFA APOF MBP LMCD1 ADAMTS9 WNT4 MBL2 IGFBP1 FGFBP1 SERPINB2 CCL20 TGFB3 WNT7B WNT5A WNT9A OLFM4 EREG CXCL10 IL1B SAA1 SAA2*
5615	extracellular space	4.76E-11	7.81E-09	29/164 17.6%	749/17782 4.2%	*CXCL8 CSF1 SERPINE1 TGFA CXCL1 CXCL3 AREG CXCL2 CX3CL1 FGF9 PLAU TIFA APOF MBP LMCD1 WNT4 MBL2 IGFBP1 FGFBP1 SERPINB2 CCL20 TGFB3 WNT5A OLFM4 EREG CXCL10 IL1B SAA1 SAA2*
5576	extracellular region	1.20E-08	8.04E-07	45/164	2026/17782	*SERPINA3 CXCL8 CSF1 NXPH3 SERPINE1 TGFA CXCL1*

	27.4%	11.3%	*CXCL3 AREG CXCL2 CX3CL1 WNT6 MMP25 MUC2 FGF9 PLAU TIFA APOF OLR1 MBP LMCD1 ADAMTS9 WNT4 MBL2 IGFBP1 FGFBP1 SERPINB2 PSAPL1 CCL20 TGFB3 WNT7B PLA2G4B LGI3 WNT5A WNT9A OLFM4 TUBA4A EREG APLN CXCL10 IL1B SAA1 SAA2 FAM20A UMODL1*

[a] *p*-value in hypergeometric test after correction

[b] The denominator represents the total number of genes with GO annotation and the numerator represents the number of genes belonging to each GO term

[c] The denominator represents the number of reference genes with GO annotation and the numerator represents the number of references genes annotated in the listed GO terms

Table S8. KEGG pathway enrichment analysis of differentially expressed genes between Caco-2 cells stimulated with wild-type spore and control.

Pathway ID	KEGG pathway	Counts	FDR [a]	Genes ID
has04657	IL-17 signaling pathway	4	0.011595132	2919, 3576, 3627, 6364
has04061	Viral protein interaction with cytokine and cytokine receptor	4	0.011595132	2919, 3576, 3627, 6364
has04130	SNARE interactions in vesicular transport	3	0.02446162	10228, 6844, 8676
has04062	Chemokine signaling pathway	4	0.037034227	2919, 3576, 3627, 6364

[a] False discovery rates

Table S9. KEGG pathway enrichment analysis of differentially expressed genes between Caco-2 stimulated with CotG-p75 and control.

Pathway ID	KEGG pathway	Counts	FDR [a]	Genes ID
has04668	TNF signaling pathway	15	7.12546E-13	1435, 2353, 2919, 2920, 2921, 330, 3383, 3553, 3627, 3659, 4792, 602, 6364, 6376, 7128
has04657	IL-17 signaling pathway	13	2.95396E-11	2353, 2919, 2920, 2921, 3553, 3576, 3627, 3934, 4792, 6364, 7128, 8061, 9641
has04064	NF-kappa B signaling pathway	13	5.89931E-11	2919, 2920, 2921, 330, 3383, 3553, 3576, 4791, 4792, 5328, 5971, 7128, 8792
has04062	Chemokine signaling pathway	14	2.49804E-09	10451, 1235, 2770, 2919, 2920, 2921, 3576, 3627, 4792, 6364, 6367, 6373, 6376, 6654
has05323	Rheumatoid arthritis	11	5.90064E-09	1435, 2353, 245972, 2919, 2920, 2921, 3383, 3553, 3576, 6364, 8792
has04061	Viral protein interaction with cytokine and cytokine receptor	11	9.48856E-09	1235, 1435, 2919, 2920, 2921, 3576, 3627, 6364, 6367, 6373, 6376
has04625	C-type lectin receptor signaling pathway	11	1.16039E-08	1959, 22808, 3553, 3659, 3708, 4791, 4792, 5971, 602, 6367, 9641
has05417	Lipid and atherosclerosis	13	5.84093E-08	10451, 1543, 2353, 2919, 2920, 2921, 3383, 3553, 3576, 3708, 4792, 4973, 9641
has05200	Pathways in cancer	18	7.04811E-08	2113, 2254, 2353, 25780, 2770, 330, 3576, 3910, 4791, 4792, 4843, 54361, 6654, 6776, 7039, 7474, 7475, 7477

has04010	MAPK signaling pathway	14	1.33198E-07	1435, 1846, 1848, 2254, 22808, 2353, 25780, 3553, 374, 4208, 4791, 5971, 6654, 7039
has04060	Cytokine-cytokine receptor interaction	14	1.33198E-07	1235, 1435, 2919, 2920, 2921, 3553, 3576, 3604, 3627, 6364, 6367, 6373, 6376, 8792
has04371	Apelin signaling pathway	10	1.25917E-06	100271849, 1958, 22808, 2770, 3708, 4208, 4843, 5054, 5581, 8862
has04621	NOD-like receptor signaling pathway	10	1.22965E-05	2919, 2920, 2921, 330, 3553, 3576, 3708, 4792, 7128, 9641
has05134	Legionellosis	7	1.36989E-05	2919, 2920, 2921, 3553, 3576, 4791, 4792
has05167	Kaposi sarcoma-associated herpesvirus infection	10	1.55592E-05	1827, 2353, 2919, 2920, 2921, 3383, 3576, 3708, 4792, 9641
has05205	Proteoglycans in cancer	10	2.23477E-05	10451, 1839, 22808, 3708, 5328, 54361, 6654, 7474, 7475, 7477
has04620	Toll-like receptor signaling pathway	8	2.23477E-05	2353, 3553, 3576, 3627, 4792, 51284, 6373, 9641
has04928	Parathyroid hormone synthesis, secretion and action	8	2.37996E-05	142680, 1839, 1958, 2353, 2770, 3708, 4208, 64386
has05120	Epithelial cell signaling in Helicobacter pylori infection	7	3.04536E-05	1839, 245972, 2919, 2920, 2921, 3576, 4792
has05166	Human T-cell leukemia virus 1 infection	10	3.04536E-05	1958, 1959, 2113, 2353, 3383, 4791, 4792, 5971, 6776, 8061
has04380	Osteoclast differentiation	8	6.66824E-05	1435, 2353, 3553, 4791, 4792, 5971, 8061,

				8792
has05224	Breast cancer	8	0.000150717	2254, 2353, 4791, 54361, 6654, 7474, 7475, 7477
has04933	AGE-RAGE signaling pathway in diabetic complications	7	0.000169009	1958, 3383, 3553, 3576, 5054, 5581, 6776
has05207	Chemical carcinogenesis - receptor activation	9	0.000169009	1543, 1545, 2254, 2353, 2770, 54658, 6654, 6776, 9641
has05146	Amoebiasis	7	0.000172423	2919, 2920, 2921, 3553, 3576, 3910, 4843
has05142	Chagas disease	7	0.000172423	2353, 2770, 3553, 3576, 4792, 4843, 5054
has05010	Alzheimer disease	11	0.000214316	100532726, 1435, 3553, 3708, 4698, 4843, 54361, 7277, 7474, 7475, 7477
has05171	Coronavirus disease - COVID-19	9	0.000270001	1839, 2353, 3553, 3576, 3627, 4153, 4792, 51284, 9641
has05164	Influenza A	8	0.000301011	100529063, 3383, 3553, 3576, 3627, 4792, 51284, 9641
has04360	Axon guidance	8	0.000410888	27289, 2770, 54361, 56963, 6091, 64221, 6585, 7474
has05133	Pertussis	6	0.000444201	2353, 2770, 3553, 3576, 3659, 4843
has04662	B cell receptor signaling pathway	6	0.000605673	10451, 2353, 25780, 27071, 4792, 6654
has04915	Estrogen signaling pathway	7	0.000667799	1839, 2353, 2770, 3708, 3872, 6654, 7039
has04012	ErbB signaling pathway	6	0.000667799	1839, 374, 6654, 6776, 685, 7039

has05162	Measles	7	0.000667799	2353, 3553, 4792, 51284, 6776, 7128, 9641
has04024	cAMP signaling pathway	8	0.001044766	10451, 2353, 2770, 338442, 4792, 5021, 6752, 8843
has04150	mTOR signaling pathway	7	0.001119884	107080638, 54361, 6654, 7474, 7475, 7477, 8140
has04390	Hippo signaling pathway	7	0.001162887	330, 374, 5054, 54361, 7474, 7475, 7477
has05022	Pathways of neurodegeneration - multiple diseases	11	0.001162887	100532726, 1435, 3553, 3708, 4698, 4843, 54361, 7277, 7474, 7475, 7477
has05161	Hepatitis B	7	0.001304395	1959, 2353, 3576, 4792, 6654, 6776, 9641
has04022	cGMP-PKG signaling pathway	7	0.001490406	100271849, 146, 2770, 3708, 4208, 4879, 5581
has05165	Human papillomavirus infection	9	0.001946628	245972, 3659, 3910, 54361, 6654, 7474, 7475, 7477, 9641
has04935	Growth hormone synthesis, secretion and action	6	0.002364751	2353, 2770, 3708, 6654, 6752, 6776
has04917	Prolactin signaling pathway	5	0.002948813	2353, 3659, 6654, 6776, 8792
has05169	Epstein-Barr virus infection	7	0.003604893	3383, 3627, 4791, 4792, 5971, 7128, 9641
has05226	Gastric cancer	6	0.005880769	2254, 54361, 6654, 7474, 7475, 7477
has05163	Human cytomegalovirus infection	7	0.005935343	2770, 3553, 3576, 3708, 4792, 6376, 6654
has04921	Oxytocin signaling pathway	6	0.006493762	1827, 2353, 2770, 3708, 4208, 5021

has04934	Cushing syndrome	6	0.006497304	2770, 3708, 54361, 7474, 7475, 7477
has04014	Ras signaling pathway	7	0.006497304	1435, 2113, 2254, 22808, 25780, 6654, 7039
has05225	Hepatocellular carcinoma	6	0.008855499	54361, 6654, 7039, 7474, 7475, 7477
has04916	Melanogenesis	5	0.009183812	2770, 54361, 7474, 7475, 7477
has05145	Toxoplasmosis	5	0.01293083	2770, 330, 3910, 4792, 4843
has04623	Cytosolic DNA-sensing pathway	4	0.019659669	3553, 3627, 4792, 9641
has05217	Basal cell carcinoma	4	0.019659669	54361, 7474, 7475, 7477
has04926	Relaxin signaling pathway	5	0.019930829	2353, 2770, 4792, 4843, 6654
has04015	Rap1 signaling pathway	6	0.020077535	10451, 1435, 2254, 22808, 25780, 2770
has04270	Vascular smooth muscle contraction	5	0.021360107	146, 3708, 4879, 5581, 72
has00830	Retinol metabolism	4	0.02227781	10170, 1543, 220, 54658
has04210	Apoptosis	5	0.02227781	2353, 330, 3708, 4792, 7277
has05135	Yersinia infection	5	0.02246513	10451, 2353, 3553, 3576, 4792
has04622	RIG-I-like receptor signaling pathway	4	0.023100838	3576, 3627, 4792, 9641
has05140	Leishmaniasis	4	0.029268427	2353, 3553, 4792, 4843
has00980	Metabolism of xenobiotics by cytochrome P450	4	0.029809529	1543, 1545, 218, 54658
has04932	Non-alcoholic fatty liver disease	5	0.031952468	100532726, 2353, 3553, 3576, 4698

has04218	Cellular senescence	5	0.03215072	2113, 22808, 3576, 3708, 5054
has05131	Shigellosis	6	0.032821821	3553, 3576, 3708, 4792, 5581, 92610
has04217	Necroptosis	5	0.032902646	100526767, 330, 3553, 6776, 7128
has05132	Salmonella infection	6	0.032902646	2353, 330, 3553, 3576, 4792, 7277
has04610	Complement and coagulation cascades	4	0.034164816	4153, 5054, 5055, 5328
has05210	Colorectal cancer	4	0.034731066	2353, 374, 6654, 7039
has04310	Wnt signaling pathway	5	0.035871726	54361, 7474, 7475, 7477, 8061
has04540	Gap junction	4	0.035871726	2770, 3708, 6654, 7277
has05222	Small cell lung cancer	4	0.039732137	330, 3910, 4792, 4843
has04912	GnRH signaling pathway	4	0.040319128	1839, 1958, 3708, 6654
has04130	SNARE interactions in vesicular transport	3	0.041724462	10228, 6844, 8676
has05020	Prion disease	6	0.04223477	100532726, 1958, 3553, 3708, 4698, 7277
has05215	Prostate cancer	4	0.04323765	4792, 5328, 6654, 7039
has05143	African trypanosomiasis	3	0.049332735	3383, 3553, 3910

[a] False discovery rates

Table S10. KEGG pathway enrichment analysis of differentially expressed genes between Caco-2 stimulated with CotG-p75 and wild-type spore.

Pathway ID	KEGG pathway	Counts	FDR [a]	Genes ID
04668	TNF signaling pathway	13	2.50425E-11	1435, 2353, 2919, 2920, 2921, 330, 3553, 3627, 3659, 4792, 6364, 6376, 7128
04010	MAPK signaling pathway	16	2.28966E-10	100137049, 1435, 1846, 1848, 2069, 2254, 2353, 25780, 3553, 374, 4208, 5321, 5971, 6654, 7039, 7043
05323	Rheumatoid arthritis	11	8.31903E-10	1435, 2353, 245972, 2919, 2920, 2921, 3553, 3576, 6364, 7043, 8792
04657	IL-17 signaling pathway	11	8.31903E-10	2353, 2919, 2920, 2921, 3553, 3576, 3627, 4792, 6364, 7128, 8061
04064	NF-kappa B signaling pathway	11	1.67879E-09	2919, 2920, 2921, 330, 3553, 3576, 4792, 5328, 5971, 7128, 8792
05200	Pathways in cancer	18	2.7479E-09	2113, 2254, 2353, 25780, 2770, 330, 3576, 4792, 4843, 54361, 6654, 6776, 7039, 7043, 7474, 7475, 7477, 7483
04062	Chemokine signaling pathway	12	2.19928E-08	10451, 2770, 2826, 2919, 2920, 2921, 3576, 3627, 4792, 6364, 6376, 6654
04060	Cytokine-cytokine receptor interaction	13	1.26664E-07	1435, 2826, 2919, 2920, 2921, 3553, 3576, 3604, 3627, 6364, 6376, 7043, 8792
04061	Viral protein interaction with cytokine and cytokine receptor	9	3.56622E-07	1435, 2826, 2919, 2920, 2921, 3576, 3627, 6364, 6376
05417	Lipid and atherosclerosis	11	6.38522E-07	10451, 1543, 2353, 2919, 2920, 2921, 3553, 3576, 3708, 4792, 4973
05226	Gastric cancer	9	5.12245E-06	2254, 4583, 54361, 6654, 7043, 7474, 7475, 7477, 7483

05207	Chemical carcinogenesis - receptor activation	10	5.12245E-06	1543, 1545, 2254, 2353, 2770, 54575, 54600, 54658, 6654, 6776
05146	Amoebiasis	8	5.12245E-06	2919, 2920, 2921, 3553, 3576, 4583, 4843, 7043
05142	Chagas disease	8	5.12245E-06	2353, 2770, 3553, 3576, 4792, 4843, 5054, 7043
04390	Hippo signaling pathway	9	5.7709E-06	330, 374, 5054, 54361, 7043, 7474, 7475, 7477, 7483
05225	Hepatocellular carcinoma	9	8.8381E-06	54361, 6654, 7039, 7043, 7474, 7475, 7477, 7483, 8193
04621	NOD-like receptor signaling pathway	9	1.66793E-05	2919, 2920, 2921, 330, 3553, 3576, 3708, 4792, 7128
04371	Apelin signaling pathway	8	2.74094E-05	1958, 2770, 3708, 4208, 4843, 5054, 5581, 8862
05205	Proteoglycans in cancer	9	3.11702E-05	10451, 3708, 5328, 54361, 6654, 7474, 7475, 7477, 7483
05224	Breast cancer	8	3.68226E-05	2254, 2353, 54361, 6654, 7474, 7475, 7477, 7483
04933	AGE-RAGE signaling pathway in diabetic complications	7	5.17715E-05	1958, 3553, 3576, 5054, 5581, 6776, 7043
05134	Legionellosis	6	5.60911E-05	2919, 2920, 2921, 3553, 3576, 4792
00830	Retinol metabolism	6	0.000120217	10170, 1543, 220, 54575, 54600, 54658
05120	Epithelial cell signaling in Helicobacter pylori infection	6	0.000131535	245972, 2919, 2920, 2921, 3576, 4792
04380	Osteoclast differentiation	7	0.000161756	1435, 2353, 3553, 4792, 5971, 8061, 8792
05167	Kaposi sarcoma-associated herpesvirus infection	8	0.000161756	1827, 2353, 2919, 2920, 2921, 3576, 3708, 4792
05133	Pertussis	6	0.00017026	2353, 2770, 3553, 3576, 3659, 4843
05010	Alzheimer disease	10	0.000195759	1435, 3553, 3708, 4843, 54361, 7277, 7474, 7475, 7477, 7483

05022	Pathways of neurodegeneration - multiple diseases	11	0.000228264	1435, 1767, 3553, 3708, 4843, 54361, 7277, 7474, 7475, 7477, 7483
05210	Colorectal cancer	6	0.000269353	2069, 2353, 374, 6654, 7039, 7043
05166	Human T-cell leukemia virus 1 infection	8	0.000287238	1958, 2113, 2353, 4792, 5971, 6776, 7043, 8061
04921	Oxytocin signaling pathway	7	0.000344894	100137049, 1827, 2353, 2770, 3708, 4208, 5321
04150	mTOR signaling pathway	7	0.000344894	54361, 6654, 7474, 7475, 7477, 7483, 8140
04934	Cushing syndrome	7	0.000344894	2770, 3708, 54361, 7474, 7475, 7477, 7483
04014	Ras signaling pathway	8	0.000362428	100137049, 1435, 2113, 2254, 25780, 5321, 6654, 7039
04217	Necroptosis	7	0.000373385	100137049, 100526767, 330, 3553, 5321, 6776, 7128
04916	Melanogenesis	6	0.000453592	2770, 54361, 7474, 7475, 7477, 7483
04928	Parathyroid hormone synthesis, secretion and action	6	0.000549687	1958, 2353, 2770, 3708, 4208, 64386
00140	Steroid hormone biosynthesis	5	0.00084235	1543, 1545, 54575, 54600, 54658
04935	Growth hormone synthesis, secretion and action	6	0.000879993	2353, 2770, 3708, 6654, 6752, 6776
05217	Basal cell carcinoma	5	0.000901488	54361, 7474, 7475, 7477, 7483
05204	Chemical carcinogenesis - DNA adducts	5	0.001226583	1543, 1545, 54575, 54600, 54658
04917	Prolactin signaling pathway	5	0.001262591	2353, 3659, 6654, 6776, 8792
05140	Leishmaniasis	5	0.001745783	2353, 3553, 4792, 4843, 7043
00980	Metabolism of xenobiotics by cytochrome P450	5	0.001788964	1543, 1545, 54575, 54600, 54658
05163	Human cytomegalovirus infection	7	0.001822812	2770, 3553, 3576, 3708, 4792, 6376, 6654
04662	B cell receptor signaling pathway	5	0.002053926	10451, 2353, 25780, 4792, 6654

05165	Human papillomavirus infection	8	0.002216921	245972, 3659, 54361, 6654, 7474, 7475, 7477, 7483
04012	ErbB signaling pathway	5	0.002244196	2069, 374, 6654, 6776, 7039
05161	Hepatitis B	6	0.002777254	2353, 3576, 4792, 6654, 6776, 7043
04912	GnRH signaling pathway	5	0.00297208	100137049, 1958, 3708, 5321, 6654
04310	Wnt signaling pathway	6	0.00297208	54361, 7474, 7475, 7477, 7483, 8061
04750	Inflammatory mediator regulation of TRP channels	5	0.003468564	100137049, 3553, 3708, 5321, 5581
04620	Toll-like receptor signaling pathway	5	0.0041226	2353, 3553, 3576, 3627, 4792
04625	C-type lectin receptor signaling pathway	5	0.0041226	3553, 3659, 3708, 4792, 5971
04360	Axon guidance	6	0.0041226	27289, 2770, 54361, 6091, 7474, 9901
01100	Metabolic pathways	16	0.004546879	100137049, 10170, 130367, 1543, 189, 220, 245972, 2571, 4837, 4843, 5321, 54575, 54600, 54658, 8707, 93432
05202	Transcriptional misregulation in cancer	6	0.005016785	1848, 330, 3576, 4208, 5328, 6495
05145	Toxoplasmosis	5	0.005027348	2770, 330, 4792, 4843, 7043
04913	Ovarian steroidogenesis	4	0.005286714	100137049, 1543, 1545, 5321
04926	Relaxin signaling pathway	5	0.007977215	2353, 2770, 4792, 4843, 6654
04730	Long-term depression	4	0.007977215	100137049, 2770, 3708, 5321
04024	cAMP signaling pathway	6	0.008106529	10451, 2353, 2770, 4792, 6752, 8843
04270	Vascular smooth muscle contraction	5	0.008519174	100137049, 146, 3708, 5321, 5581
04210	Apoptosis	5	0.00907171	2353, 330, 3708, 4792, 7277
05135	Yersinia infection	5	0.009166798	10451, 2353, 3553, 3576, 4792

04915	Estrogen signaling pathway	5	0.009263107	2353, 2770, 3708, 6654, 7039
05162	Measles	5	0.00936062	2353, 3553, 4792, 6776, 7128
05171	Coronavirus disease - COVID-19	6	0.009440184	2353, 3553, 3576, 3627, 4153, 4792
04664	Fc epsilon RI signaling pathway	4	0.009899859	100137049, 10451, 5321, 6654
04550	Signaling pathways regulating pluripotency of stem cells	5	0.009899859	54361, 7474, 7475, 7477, 7483
05211	Renal cell carcinoma	4	0.010047825	2113, 6654, 7039, 7043
05131	Shigellosis	6	0.011593391	3553, 3576, 3708, 4792, 5581, 92610
05132	Salmonella infection	6	0.011826377	2353, 330, 3553, 3576, 4792, 7277
05220	Chronic myeloid leukemia	4	0.01251292	4792, 6654, 6776, 7043
04218	Cellular senescence	5	0.01251292	2113, 3576, 3708, 5054, 7043
04022	cGMP-PKG signaling pathway	5	0.015615484	146, 2770, 3708, 4208, 5581
04610	Complement and coagulation cascades	4	0.016284844	4153, 5054, 5055, 5328
04540	Gap junction	4	0.01764484	2770, 3708, 6654, 7277
04976	Bile secretion	4	0.017959082	54575, 54600, 54658, 9429
05152	Tuberculosis	5	0.019168941	245972, 3553, 3656, 4843, 7043
00053	Ascorbate and aldarate metabolism	3	0.02080148	54575, 54600, 54658
05215	Prostate cancer	4	0.021515156	4792, 5328, 6654, 7039
04666	Fc gamma R-mediated phagocytosis	4	0.021515156	100137049, 10451, 5321, 5581
05231	Choline metabolism in cancer	4	0.021849107	100137049, 2353, 5321, 6654
05130	Pathogenic Escherichia coli infection	5	0.024472205	2353, 3553, 3576, 4792, 7277
00040	Pentose and glucuronate interconversions	3	0.024641226	54575, 54600, 54658

04660	T cell receptor signaling pathway	4	0.024700083	10451, 2353, 4792, 6654
04659	Th17 cell differentiation	4	0.026324568	2353, 3553, 4792, 6776
04015	Rap1 signaling pathway	5	0.02892787	10451, 1435, 2254, 25780, 2770
04724	Glutamatergic synapse	4	0.030406361	100137049, 2770, 3708, 5321
04726	Serotonergic synapse	4	0.030770862	100137049, 2770, 3708, 5321
00860	Porphyrin and chlorophyll metabolism	3	0.035375045	54575, 54600, 54658
04611	Platelet activation	4	0.036644255	100137049, 2770, 3708, 5321
04151	PI3K-Akt signaling pathway	6	0.037738474	1435, 2069, 2254, 374, 6654, 7039
04020	Calcium signaling pathway	5	0.041969271	146, 2254, 3708, 4843, 5724
05144	Malaria	3	0.044599339	3553, 3576, 7043

[a] False discovery rates

Table S11. Primers used for RT-qPCR for selected genes.

Accession number	Gene symbols	Forward primer (5’→3’)	Reverse primer (5’→3’)
NM_001657	*AREG*	GCACCTGGAAGCAGTAACATGC	GGCAGCTATGGCTGCTAATGCA
NM_003236	*TGFA*	GGTCCGAAAACACTGTGAGTGG	CAAACTCCTCCTCTGGGCTCTT
NM_001729	*BTC*	CCTCTTCGGAAACGTCGTAAAAG	CTTGCCACCAACCTGGAGGTAA
NM_001945	*HBEGF*	TGTATCCACGGACCAGCTGCTA	TGCTCCTCCTTGTTTGGTGTGG
NM_001165	*BIRC3*	GCTTTTGCTGTGATGGTGGACTC	CTTGACGGATGAACTCCTGTCC
NM_020529	*NFKBIA*	TCCACTCCATCCTGAAGGCTAC	CAAGGACACCAAAAGCTCCACG
NM_006290	*TNFAIP3*	CTCAACTGGTGTCGAGAAGTCC	TTCCTTGAGCGTGCTGAACAGC
NM_002502	*NFKB2*	GGCAGACCAGTGTCATTGAGCA	CAGCAGAAAGCTCACCACACTC
NM_006509	*RELB*	TGTGGTGAGGATCTGCTTCCAG	TCGGCAAATCCGCAGCTCTGAT
NM_001511	*CXCL1*	AGCTTGCCTCAATCCTGCATCC	TCCTTCAGGAACAGCCACCAGT
NM_002089	*CXCL2*	GGCAGAAAGCTTGTCTCAACCC	CTCCTTCAGGAACAGCCACCAA
NM_002090	*CXCL3*	TTCACCTCAAGAACATCCAAAGTG	TTCTTCCCATTCTTGAGTGTGGC
NM_000584	*CXCL8*	GAGAGTGATTGAGAGTGGACCAC	CACAACCCTCTGCACCCAGTTT
NM_001565	*CXCL10*	GGTGAGAAGAGATGTCTGAATCC	GTCCATCCTTGGAAGCACTGCA
NM_005409	*CXCL11*	AAGGACAACGATGCCTAAATCCC	CAGATGCCCTTTTCCAGGACTTC
NM_004591	*CCL20*	AAGTTGTCTGTGTGCGCAAATCC	CCATTCCAGAAAAGCCACAGTTTT
NM_000757	*CSF1*	TGAGACACCTCTCCAGTTGCTG	GCAATCAGGCTTGGTCACCACA

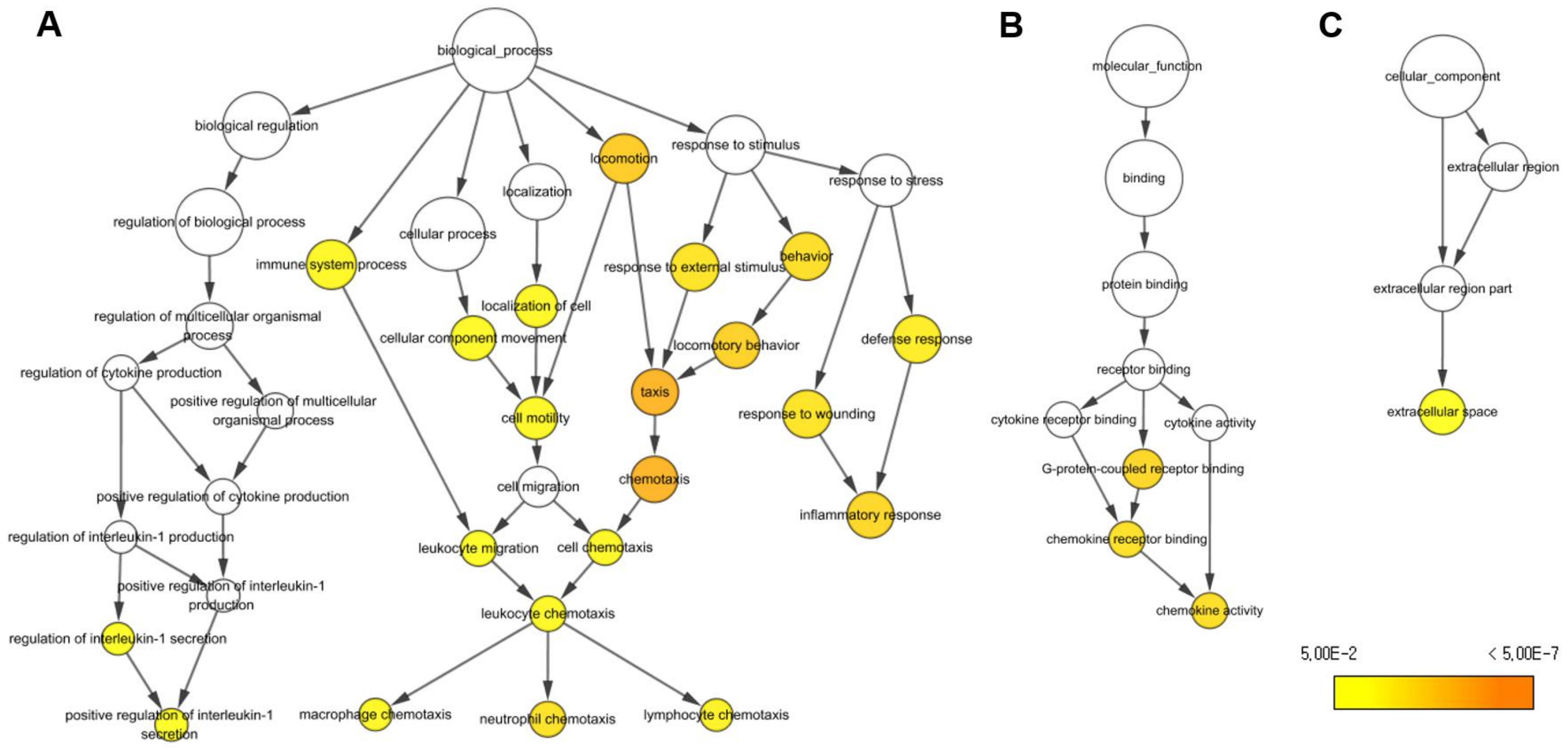

Figure S1. Illustration of Gene Ontology (GO) term networks of differentially expressed genes between wild-type spore treated Caco-2 and control. The networks were generated by Cytoscape BiNGO. All significantly enriched GO terms in biological process (A), molecular function (B), and cellular component (C) are presented. Circle size represents GO hierarchy. Yellow shade represents enrichment level. The threshold of hypergeometric distribution of the functional annotation was set at corrected *p*-value < 0.05.

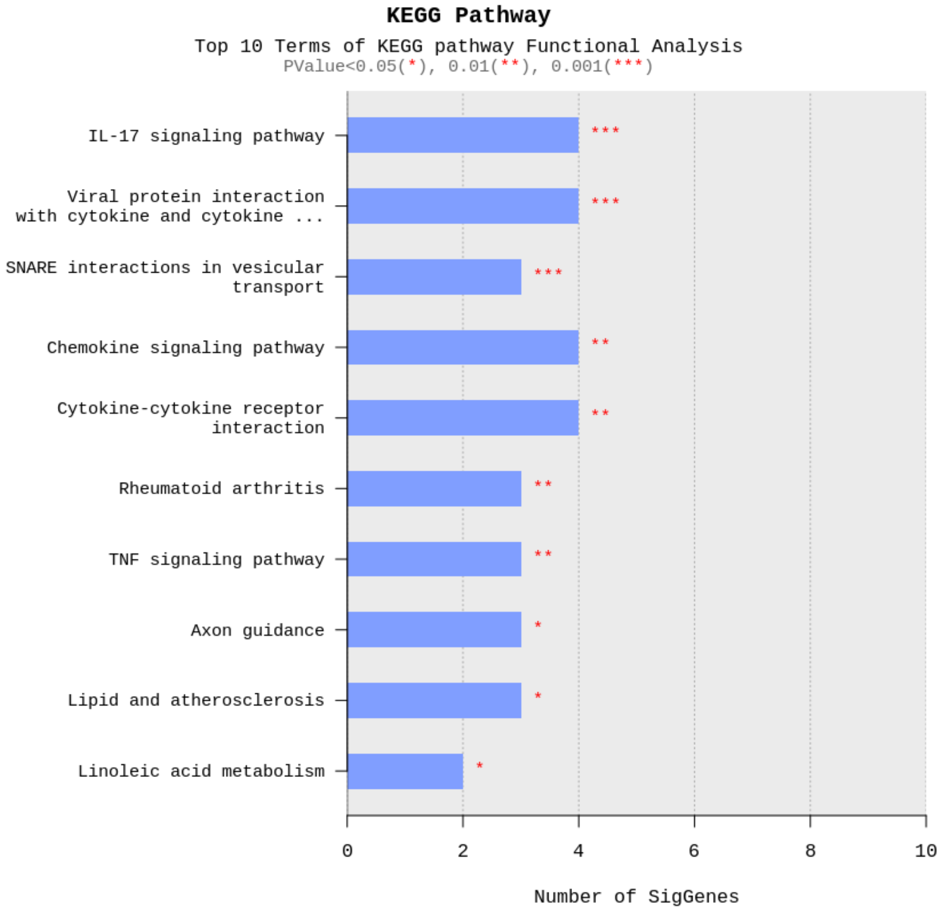

Figure S2. Top 10 KEGG pathways enriched in differentially expressed genes between wild-type spore treated Caco-2 and control. Asterisks (*) respresent a significance difference from the control (* $p < 0.05$, ** $p < 0.01$, *** $p < 0.001$).

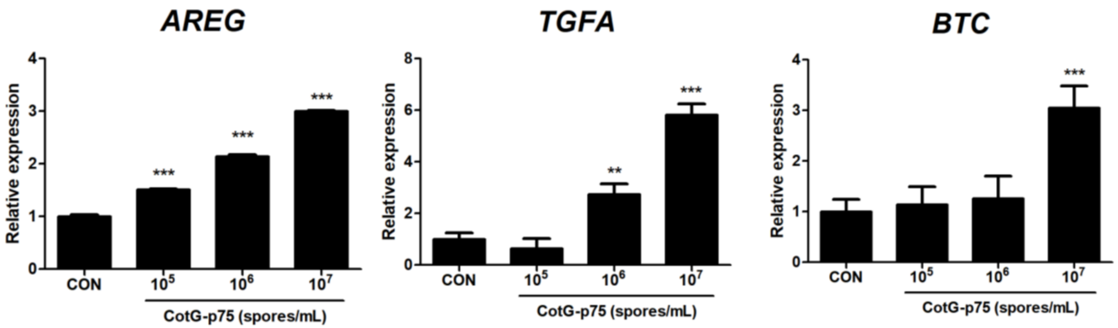

Figure S3. The mRNA expression of the genes involved in EGFR signaling from Caco-2 cells stimulated by different doses of CotG-p75. Caco-2 cells were incubated with 10^5, 10^6, and 10^7 spores/mL of CotG-p75 for 3 h. The relative expression level was determined by real-time quantitative PCR. RT-qPCR data presented as mean ± standard deviation (n = 3). Statistical analysis was performed by a one-way ANOVA and Tukey-Kramer post-hoc analysis. Asterisks (*) indicate a significance difference from the control (* $p < 0.05$, ** $p < 0.01$, *** $p < 0.001$).

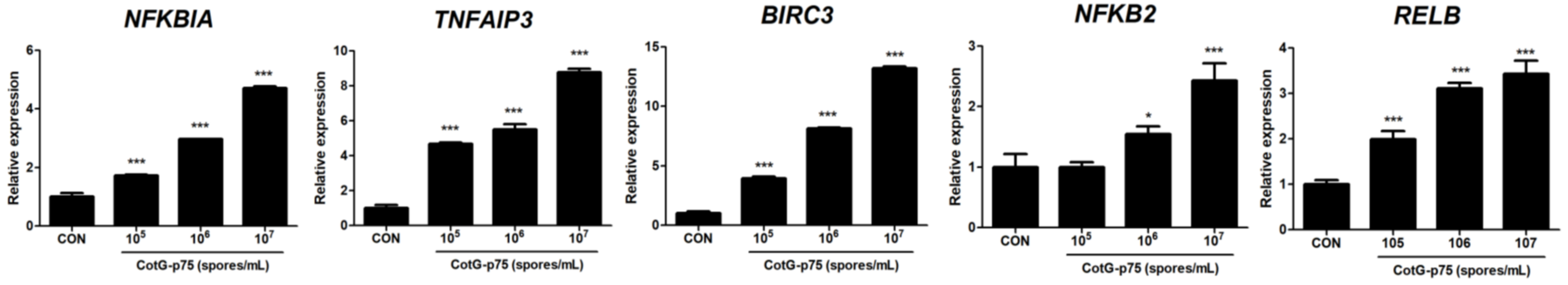

Figure S4. The mRNA expression of the genes involved in NF-κB signaling from Caco-2 cells stimulated by different doses of CotG-p75. Caco-2 cells were incubated with 10^5, 10^6, and 10^7 spores/mL of CotG-p75 for 3 h. The relative expression level was determined by real-time quantitative PCR. RT-qPCR data presented as mean ± standard deviation (n = 3). Statistical analysis was performed by a one-way ANOVA and Tukey-Kramer post-hoc analysis. Asterisks (*) indicate a significance difference from the control (* $p < 0.05$, ** $p < 0.01$, *** $p < 0.001$).

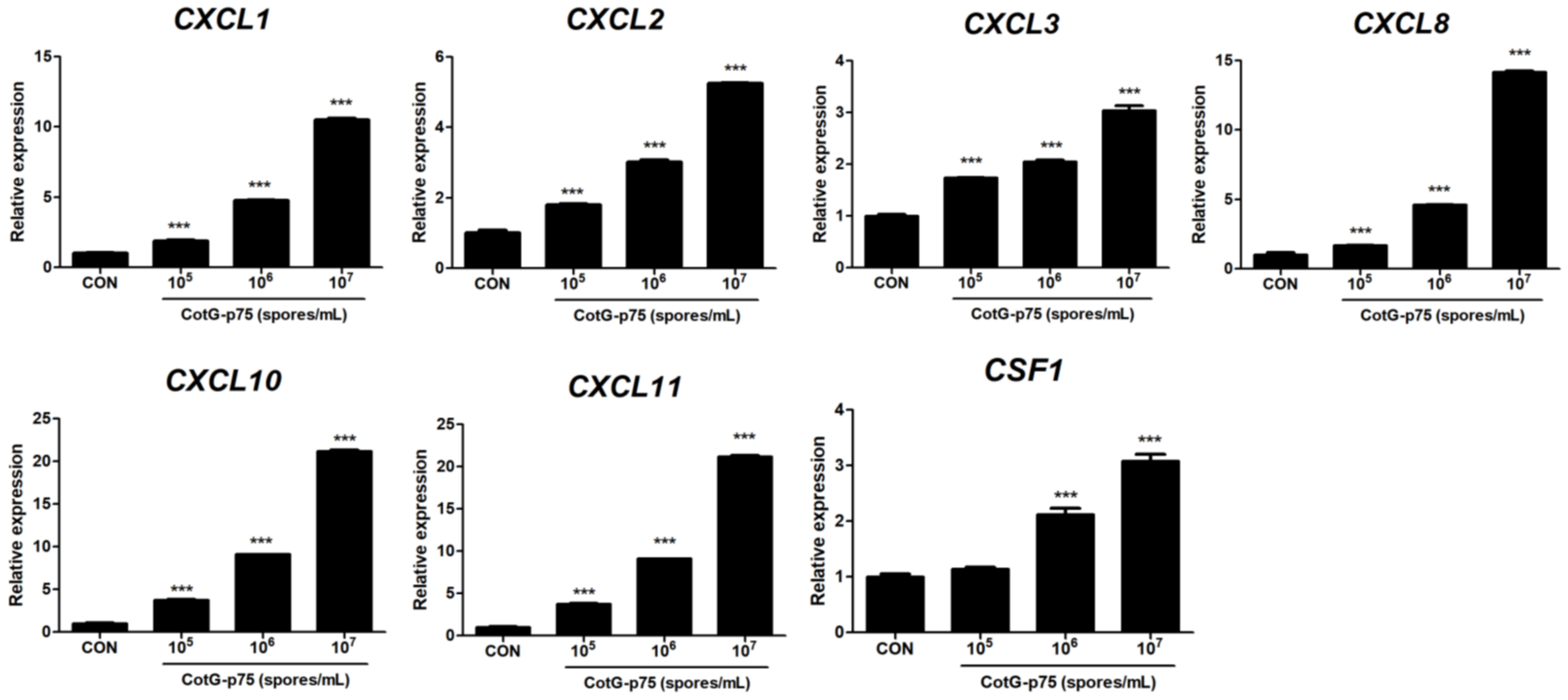

Figure S5. The mRNA expression of the genes involved in immune response from Caco-2 cells stimulated by different doses of CotG-p75. Caco-2 cells were incubated with 10^5, 10^6, and 10^7 spores/mL of CotG-p75 for 3 h. The relative expression level was determined by real-time quantitative PCR. RT-qPCR data presented as mean ± standard deviation (n = 3). Statistical analysis was performed by a one-way ANOVA and Tukey-Kramer post-hoc analysis. Asterisks (*) indicate a significance difference from the control (* $p < 0.05$, ** $p < 0.01$, *** $p < 0.001$).

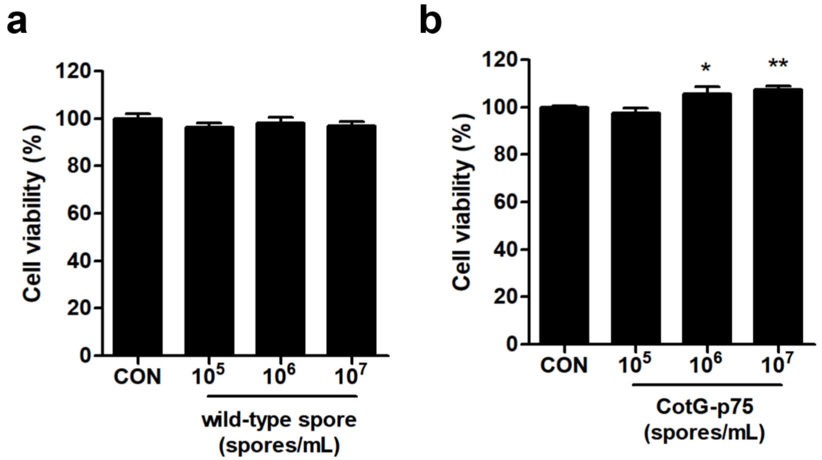

Figure S6. Effect of wild-type spores and CotG-p75 on cell viability of intestinal epithelial Caco-2 cells. Cell viability was determined after 24 h incubation with each spore. Each column represents the mean ± standard deviation with respect to 100% control. All experiments were performed independently in triplicates. Statistical analysis was performed by a one-way ANOVA and Tukey-Kramer post-hoc analysis. Asterisks (*) indicate a significance difference from the control (* $p < 0.05$, ** $p < 0.01$, *** $p < 0.001$).
